# A bioinspired, one-step total synthesis of peshawaraquinone†

**DOI:** 10.1039/d2sc05377b

**Published:** 2022-12-21

**Authors:** Tomás Vieira de Castro, David M. Huang, Christopher J. Sumby, Andrew L. Lawrence, Jonathan H. George

**Affiliations:** a Department of Chemistry, University of Adelaide Adelaide SA 5000 Australia jonathan.george@adelaide.edu.au; b EaStCHEM School of Chemistry, University of Edinburgh Joseph Black Building, David Brewster Road Edinburgh EH9 3FJ UK

## Abstract

A concise synthesis of a stereochemically complex meroterpenoid, peshawaraquinone, *via* the unsymmetrical dimerization of its achiral precursor, dehydro-α-lapachone, is reported. Enabled by reversible oxa-6π-electrocyclizations of 2*H*-pyran intermediates, the base-catalyzed dimerization sets up an intramolecular (3 + 2) cycloaddition, with the formation of six stereocenters during the cascade. Combining the generation and *in situ* dimerization of dehydro-α-lapachone allows a one-step total synthesis of peshawaraquinone from lawsone and prenal.

## Abstract 2

A new proposal for the biosynthesis of peshawaraquinone *via* the unsymmetrical dimerization of dehydro-α-lapachone led to its total synthesis in one step from inexpensive starting materials.

## Graphical abstract

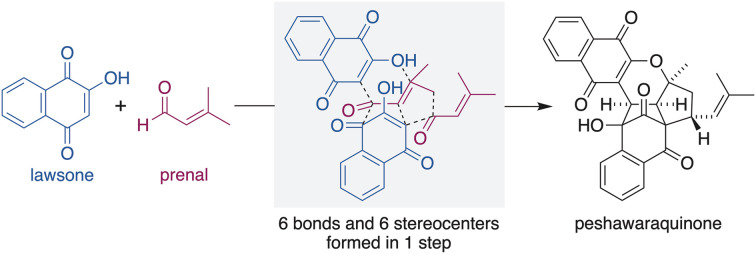

## Introduction

**Fig. 1:**
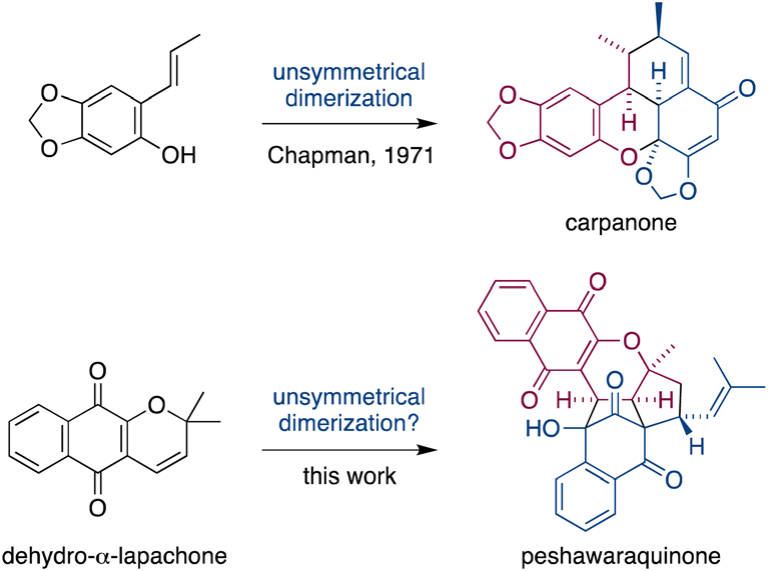
Unsymmetrical dimerizations of achiral intermediates in Chapman's biomimetic synthesis of carpanone, and in our proposed synthesis of peshawaraquinone.

Dimerization occurs frequently in the biosynthesis of complex natural products, and this has often been exploited in the field of biomimetic total synthesis.^1^ However, unsymmetrical dimerizations of *achiral* intermediates that form *stereochemically complex* natural products (*i.e.* those with multiple stereocenters) are rare.^2^ A famous example is Chapman's total synthesis of carpanone *via* an unsymmetrical, oxidative dimerization of a simple phenol that constructs five stereocenters and two rings in a single step (Fig. 1).^3^

**Scheme 1:**
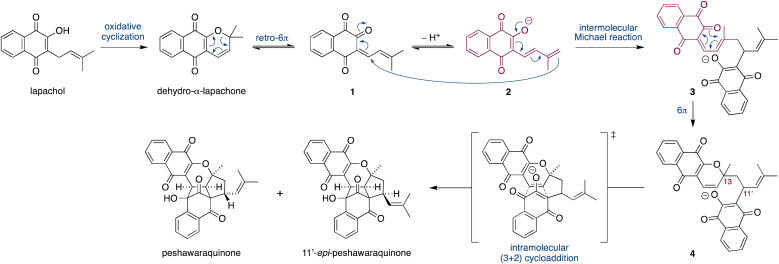
Proposed biosynthesis of peshawaraquinone *via* the dimerization of dehydro-α-lapachone.

Herein, we propose that a complex meroterpenoid, peshawaraquinone, is biosynthesized *via* an unsymmetrical dimerization of dehydro-α-lapachone, a naturally occurring naphthoquinone. Peshawaraquinone was isolated from the heartwood of *Fernandoa adenophylla*,^4^ a flowering tree widely used in traditional medicine, alongside the more common dehydro-α-lapachone.^5^ Its elaborate, polycyclic structure featuring six stereocenters and seven rings was elucidated by NMR and X-ray crystallographic studies. A detailed mechanistic proposal for the biosynthesis of peshawaraquinone is outlined in Scheme 1. First, dehydro-α-lapachone is formed by oxidative cyclization of lapachol,^6^ a historically significant meroterpenoid first isolated by Paternò in 1882 and later revised in structure by Hooker,^7^ who also used lapachol as the original substrate for his eponymous oxidation.^8^ Next, retro-oxa-6π-electrocyclization of dehydro-α-lapachone gives a reactive 1-oxatriene intermediate 1. Similar electrocyclic ring opening of 2*H*-pyrans are known to be thermodynamically accessible,^9^ and dehydro-α-lapachone itself has been shown to isomerize *via* Lewis acid catalyzed retro-6π-electrocyclization.^10^ Deprotonation of 1-oxatriene 1 to give an extended enolate 2 then sets up the key unsymmetrical dimerization *via* an intermolecular bisvinylogous Michael reaction to form the first dimeric intermediate 3,^11^ while regenerating a 1-oxatriene motif that undergoes an oxa-6π-electrocyclization to give 2*H*-pyran 4. We predict that the oxa-6π-electrocyclization of 3 is unlikely to be highly diastereoselective (*i.e.* torqueselective), so that 4 should be formed as a mixture of diastereomers that differ in their relative configurations at C-13 and C-11′. Next, a concerted but asynchronous, intramolecular (3 + 2) cycloaddition between the hydroxynaphthoquinone and the 2*H*-pyran of 4 gives peshawaraquinone.^12^ Our biosynthetic proposal therefore also suggests that the diastereomer 11′-*epi*-peshawaraquinone could be a previously unrecognized natural product.^13^ While mechanistically related intramolecular (3 + 2) cycloadditions of hydroxynaphthoquinones are synthetically known, the functionalization of a 2,2-dimethyl-2*H*-pyran *via* a retro-oxa-6π-electrocyclization, deprotonation, alkylation and oxa-6π-electrocyclization is unprecedented. We further speculated that the unsymmetrical dimerization of dehydro-α-lapachone is unlikely to be enzyme catalyzed in nature, and that 3 and all subsequent chiral compounds in the pathway are therefore formed as racemates.^14^ No optical rotation data for natural peshawaraquinone was reported, but an image of the structure in a biological activity paper^4*b*^ indicates that it crystallized in the centrosymmetric monoclinic space group *C*2/*c* and it is therefore racemic.^15^

## Results and discussion

**Scheme 2:**
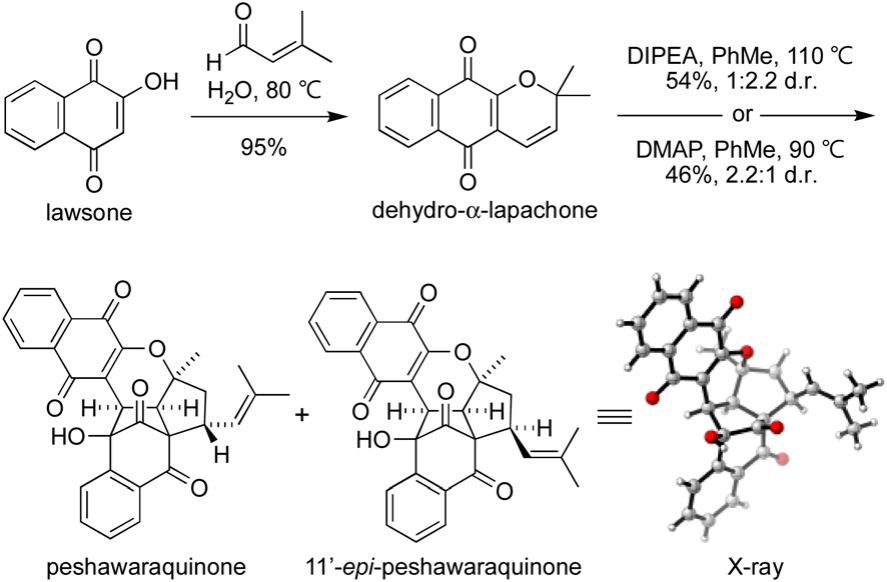
Dimerization of dehydro-α-lapachone.

To investigate the chemical feasibility of our biosynthetic hypothesis, we conducted a biomimetic synthesis of peshawaraquinone (Scheme 2). First, dehydro-α-lapachone was synthesized by Knoevenagel condensation of lawsone with prenal and subsequent oxa-6π-electrocyclization. This reaction was conveniently carried out on a 10 g scale using Lee's “on-water” procedure.^16^ After extensive screening of acid/base catalysts and thermal/photochemical conditions for the dimerization of dehydro-α-lapachone, we found that tertiary amine bases in PhMe at high temperature mediated efficient conversion to a mixture of peshawaraquinone and 11′-*epi*-peshawaraquinone.^17^ For example, heating dehydro-α-lapachone in PhMe at reflux in the presence of one equivalent of *N*,*N*-diisopropylethylamine (DIPEA) gave peshawaraquinone and its C-11′-epimer in 54% combined yield (1 : 2.2 d.r. in favor of the epimer) after flash column chromatography with neat CH_2_Cl_2_ as the eluent. Alternatively, use of 4-dimethylaminopyridine (DMAP) as the base gave a 2.2 : 1 d.r. in favor of the natural product, in 46% combined yield. The nature of the ammonium cation therefore appears to subtly influence the diastereoselectivity of the oxa-6π-electrocyclization of the dimeric intermediate 3. The dimerization did not proceed to full conversion, with dehydro-α-lapachone starting material typically recovered in 10–15% yield. NMR spectra of the crude reaction products are clean, showing product formation and unreacted starting material as the only compounds present in significant amounts.^18^ Both reactions were conducted on multi-gram scale. Analytical samples of peshawaraquinone and the epimer were obtained by preparative thin layer chromatography or by repeated flash column chromatography with hexane-CH_2_Cl_2_. From the DMAP-catalyzed dimerization, pure peshawaraquinone was obtained in 20% isolated yield. NMR spectra for synthetic peshawaraquinone fully matched the isolation data, while the structure of 11′-*epi*-peshawaraquinone was proven by single crystal X-ray crystallography.^19^ A trace of 11′-*epi*-peshawaraquinone can be observed in the NMR spectrum of isolated peshawaraquinone,^4*b*^ which supports our hypothesis that both compounds are natural products derived from dimerization of dehydro-α-lapachone. Although the synthesis of peshawaraquinone was conducted in PhMe at reflux to maximize conversion, some product formation was observed at room temperature (*e.g.* 3% formation of the natural product on treating dehydro-α-lapachone with DMAP in PhMe at rt for 24 h).

**Scheme 3:**
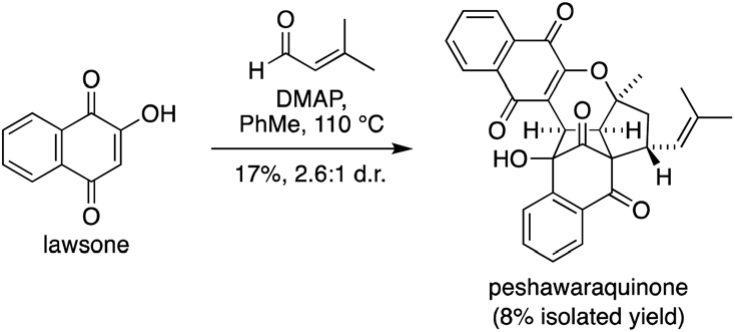
One-step total synthesis of peshawaraquinone.

Given the success of our two-step approach, we also investigated a one-step total synthesis of peshawaraquinone (Scheme 3). Using DMAP in PhMe at 110 °C, lawsone and a slight excess of prenal were converted into a 2.6 : 1 mixture of peshawaraquinone and its C-11′ epimer in 17% combined yield, with an 8% yield of the pure natural product obtained by repeated flash column chromatography. Although low yielding, this reaction meets several requirements of an “ideal synthesis” as stated by Wender,^20^ with a one-step total synthesis of a complex target from inexpensive, readily available starting materials under simple conditions. The four-component cascade reaction generates six bonds and six stereocenters in one step, enabled by the conversion of six trigonal planar carbon atoms in the reactants to six tetrahedral centers in the product.

**Scheme 4:**
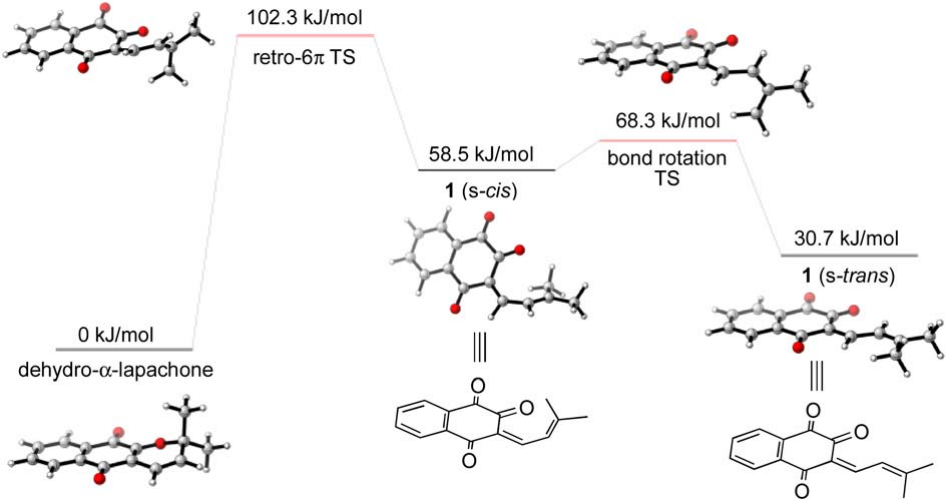
Computational analysis of the thermal retro-oxa-6π-electrocyclization of dehydro-α-lapachone.

To gain further insight into the mechanism of the synthesis and biosynthesis of peshawaraquinone, molecular geometries and energies of proposed intermediates were calculated by density functional theory (DFT) using the ORCA quantum chemistry software package (version 5.0.3).^21^ Geometries were optimized in the gas phase using the ωB97X-D3 functional^22^ and def2-SVP basis set.^23^ Transition states were obtained using a combination of the climbing image nudged elastic band method and an eigenvector-following optimization of the climbing image.^24^ Single-point energy calculations of the optimized geometries were also carried out using the same functional with the def2-TZVPD basis set^25^ and the SMD continuum solvent model^26^ with toluene as the solvent. Firstly, our calculations show that retro-oxa-6π-electrocyclization of dehydro-α-lapachone is thermally accessible but with Δ*G*^‡^ = 102.3 kJ mol^−1^ that is probably rate-limiting for the overall cascade.^27^ Bond rotation required to convert the initially formed s-*cis* conformation of 1-oxatriene 1 to the more stable s-*trans* conformation is much faster than the retro-oxa-6π-electrocyclization, so any subsequent dimerization reactions probably involve 1 (s-*trans*) (Scheme 4).

**Scheme 5:**
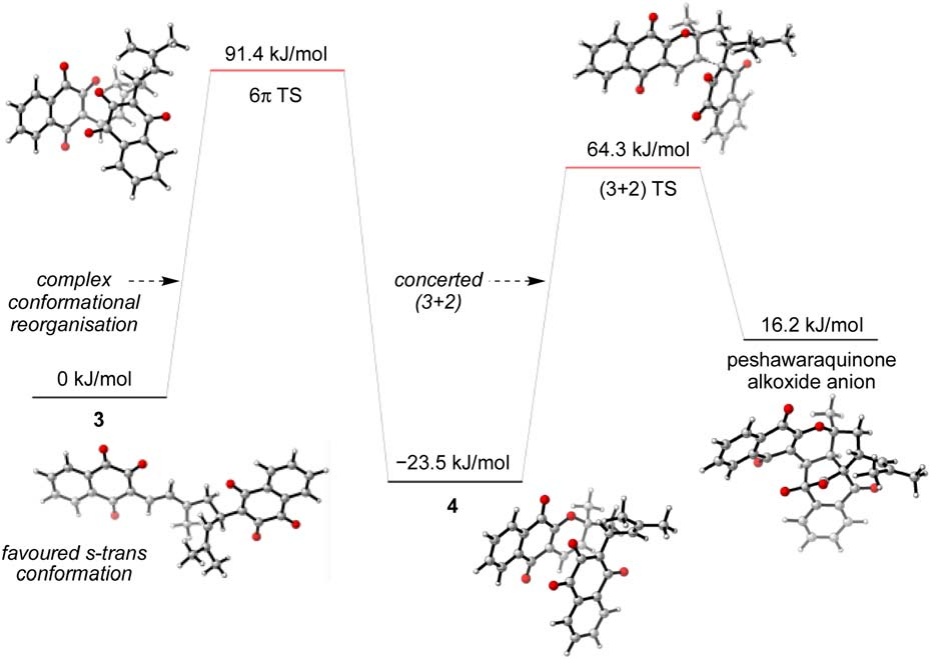
Computational analysis of the oxa-6π-electrocyclization and concerted (3 + 2) cycloaddition steps.

Given the number of possible alkene configurations and the conformational flexibility of both 1-oxatriene 1 and extended enolate 2, the precise mechanism of the dimerization was not modelled. Instead, we calculated a complex series of bond rotations that convert the favored s-*trans* conformation of the unsymmetrical dimer 3 to the sterically disfavored s-*cis* conformation required to set up the exergonic oxa-6π-electrocyclization leading to 4, with an overall Δ*G*^‡^ = 91.4 kJ mol^−1^.^28^ Finally, the intramolecular (3 + 2) cycloaddition of 4 was calculated to be a concerted but asynchronous process, to give the alkoxide anion of peshawaraquinone which is then favorably protonated to complete the cascade (Scheme 5). Although one bond is more fully formed, there is only a single transition state in the conversion of 4 into the alkoxide anion of peshawaraquinone, so the mechanism does not appear to be a stepwise sequence of intramolecular Michael and aldol reactions. The calculated reaction profile leading to 11′-*epi*-peshawaraquinone *via* a stereodivergent oxa-6π-electrocyclization of 3 is very similar in energy, thus rationalizing the formation of both diastereomers in our biomimetic synthesis.

## Conclusions

In summary, we have discovered a remarkably simple synthesis of a complex meroterpenoid, peshawaraquinone, *via* the unsymmetrical dimerization of a 2*H*-pyran monomer. Our work highlights the power of biomimetic synthesis to interrogate biosynthetic pathways, while also assisting in the discovery of new natural products. In addition to the rapid generation of stereochemical complexity, this biomimetic cascade features a novel alkylation of seemingly inert methyl group of the 2,2-dimethyl-2*H*-pyran of dehydro-α-lapachone that is facilitated by reversible oxa-6π-electrocyclic reactions.^29^

## Data availability

All experimental procedures, spectral data and computational calculations are available in the ESI.†

## Author contributions

The manuscript was written through contributions of all authors. All authors have given approval to the final version of the manuscript.

## Conflicts of interest

There are no conflicts to declare.

## Supplementary Material

SC-014-D2SC05377B-s001

SC-014-D2SC05377B-s002
