# Supplementary material for: A bioinspired, one-step total synthesis of peshawaraquinone
Source: Chem Sci. 2022 Dec 21;14(4):950–4. doi: 10.1039/d2sc05377b (PMC9890946; doi:10.1039/d2sc05377b)
Supplement: SC-014-D2SC05377B-s001 [file SC-014-D2SC05377B-s001.pdf]

Electronic Supplementary Material (ESI) for Chemical Science.
This journal is © The Royal Society of Chemistry 2022

A Bioinspired, One-Step Total Synthesis of Peshawaraquinone

Tomás Vieira de Castro,[1,2] David M. Huang,[1] Christopher J. Sumby,[1] Andrew L. Lawrence,[2] and Jonathan H. George[1,*]

[1]Department of Chemistry, University of Adelaide, Adelaide, SA 5005, Australia.

[2]EaStCHEM School of Chemistry, University of Edinburgh, Joseph Black Building, David Brewster Road, Edinburgh EH9 3FJ, United Kingdom.

Supporting Information

Table of Contents

1. General Methods 2
2. Experimental Procedures 3
3. Dimerization Screening 6
4. NMR Spectra 13
5. NMR Assignments 21
6. Single Crystal X-ray Crystallography 23
7. Computational Methods 26
8. References 62

1. General Methods

All chemicals were purchased from commercial suppliers and used as received. All organic extracts were dried over anhydrous sodium sulfate. Thin layer chromatography was performed using aluminium sheets coated with silica gel F254. Visualization was aided by viewing under a UV lamp and staining with *p*-anisaldehyde stain followed by heating. All R_f values were measured to the nearest 0.05. Flash column chromatography was performed using 40-63-micron grade silica gel. Infrared spectra were recorded using an FT-IR spectrometer as the neat compounds. High field NMR spectra were recorded using either a 500 MHz spectrometer (^1H at 500 MHz, ^{13}C at 126 MHz) or 600 MHz spectrometer (^1H at 600 MHz, ^{13}C at 151 MHz). The solvent used for NMR spectra was $CDCl_3$ unless otherwise specified. ^1H chemical shifts are reported in ppm on the δ-scale relative to TMS (δ 0.0) or residual $CHCl_3$ (δ 7.26) and ^{13}C NMR chemical shifts are reported in ppm relative to $CDCl_3$ (δ 77.16). Multiplicities are reported as (br) broad, (s) singlet, (d) doublet, (t) triplet, (q) quartet, (quin) quintet, (sext) sextet, (hept) heptet and (m) multiplet. All *J*-values were rounded to the nearest 0.1 Hz. ESI high resolution mass spectra were recorded on an Agilent 6230 TOF LC/MS mass spectrometer.

2. Experimental Procedures

Synthesis of dehydro-α-lapachone:

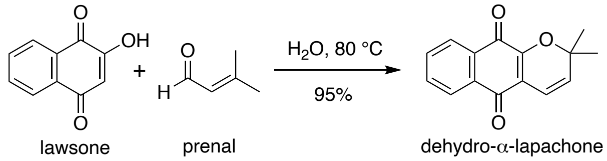

Prepared according to a modified literature procedure.[1]

To a suspension of lawsone (10.0 g, 57.4 mmol) in H_2O (500 mL) was added prenal (11.1 mL, 115 mmol) at room temperature. This mixture was stirred at 80 °C for 6 h. Upon completion, the reaction mixture was cooled with ice and the solid, orange product was filtered and washed rigorously with ice-cold water. Following filtration, the orange solid was dried under vacuum for 24 h affording dehydro-α-lapachone (13.1 g, 54.4 mmol, 95%). Spectroscopic data was in agreement with literature values.[1]

Data for dehydro-α-lapachone:

R_f 0.45 (3:1 petroleum spirit 60-80 / ethyl acetate)

IR (neat) ν_{max}: 2965, 1671, 1569, 1326, 1274, 1131, 968, 714 cm^{-1}

^{1}H NMR (500 MHz, $CDCl_3$) δ 8.09 (dd, J = 7.1, 1.9 Hz, 2H), 7.74 – 7.64 (m, 2H), 6.65 (d, J = 9.9 Hz, 1H), 5.72 (d, J = 10.0 Hz, 1H), 1.55 (s, 6H);

^{13}C NMR (126 MHz, $CDCl_3$) δ 182.0, 180.0, 152.6, 134.1, 133.3, 131.7, 131.7, 131.0, 126.4, 118.0, 115.6, 80.6, 28.5.

HRMS (ESI) m/z: [M+H] calculated 241.0859, found 241.0851.

Dimerization of dehydro-α-lapachone with DMAP:

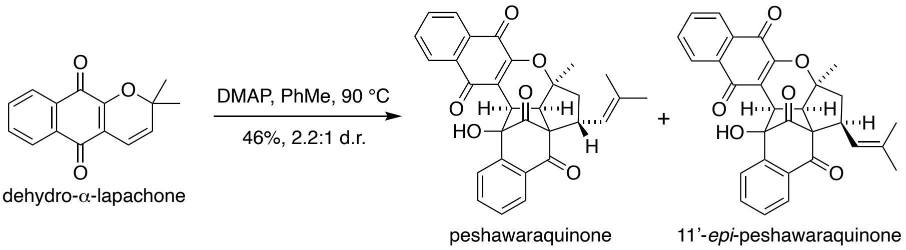

To a solution of dehydro-α-lapachone (3.00 g, 12.5 mmol) in PhMe (125 mL) was added 4-dimethylaminopyridine (DMAP) (1.52 g, 12.5 mmol) and the reaction mixture was heated at 90 °C for 18 h. This mixture was cooled to room temperature and then directly purified by flash chromatography on silica gel with CH_2Cl_2 as the eluent (prior evaporation of the PhMe solvent is unnecessary) to give a 2.2:1 mixture of peshawaraquinone and 11'-*epi*-peshawaraquinone (1.41 g, 46%). Recovered dehydro-α-lapachone starting material was also obtained (301 mg, 10%). Pure peshawaraquinone was obtained (602 mg, 20%) by further flash chromatography on silica gel (1:1 hexanes-CH_2Cl_2 to neat CH_2Cl_2, gradient elution). Spectroscopic data for peshawaraquinone is in agreement with literature values.[2]

Data for peshawaraquinone:

$\mathbf{R_f}$ 0.30 (CH_2Cl_2)

IR (neat) ν_{max}: 3431, 2921, 1768, 1680, 1255, 1180, 908, 724 cm^{-1}

^{1}H NMR (500 MHz, $CDCl_3$) δ 8.18 (d, J = 6.8 Hz, 1H), 8.13 (d, J = 7.7 Hz, 1H), 8.06 (d, J = 7.8 Hz, 1H), 8.00 (d, J = 7.8 Hz, 1H), 7.80 – 7.70 (m, 3H), 7.53 (t, J = 7.5 Hz, 1H), 5.96 (d, J = 9.7 Hz, 1H), 3.87 (d, J = 10.4 Hz, 1H), 3.87 – 3.79 (m, 1H), 3.57 (s, 1H), 2.78 (d, J = 10.2 Hz, 1H), 2.64 (dd, J = 13.6, 6.4 Hz, 1H), 2.18 (t, J = 13.2 Hz, 1H), 1.78 (s, 3H), 1.77 (s, 3H), 1.32 (s, 3H).

^{13}C NMR (126 MHz, $CDCl_3$) δ 203.8, 193.6, 184.5, 179.2, 155.1, 144.8, 135.8, 134.4, 134.0, 133.8, 132.1, 131.3, 129.9, 129.3, 127.8, 126.8, 124.9, 122.5, 120.8, 87.2, 86.0, 73.6, 53.7, 48.9, 37.0, 35.7, 26.4, 21.7, 18.2.

HRMS (ESI) m/z: [M+H] calculated 481.1646, found 481.1645

Data for 11'-*epi*-peshawaraquinone:

$\mathbf{R_f}$ 0.25 (CH_2Cl_2)

IR (neat) ν_{max}: 3447, 2922, 1764, 1680, 977, 726 cm^{-1}

^{1}H NMR (500 MHz, $CDCl_3$) δ 8.18 (d, J = 7.4 Hz, 1H), 8.14 (d, J = 7.4 Hz, 1H), 8.10 (d, J = 7.8 Hz, 1H), 7.99 (d, J = 7.8 Hz, 1H), 7.82 – 7.70 (m, 3H), 7.54 (t, J = 7.7 Hz, 1H), 5.60 (d, J = 10.3 Hz, 1H), 4.35 (td, J = 9.9, 5.1 Hz, 1H), 3.88 (d, J = 10.1 Hz, 1H), 3.48 (s, 1H), 2.65 (d, J = 10.2 Hz, 1H), 2.49 (dd, J = 14.4, 5.1 Hz, 1H), 2.41 (dd, J = 14.4, 9.6 Hz, 1H), 1.76 (s, 3H), 1.73 (s, 3H), 1.34 (s, 3H).

^{13}C NMR (151 MHz, $CDCl_3$) δ 202.6, 193.2, 184.2, 179.2, 154.5, 146.7, 136.1, 134.4, 133.7, 133.4, 132.0, 131.3, 129.2, 128.8, 128.0, 126.8, 126.7, 124.6, 123.2, 121.3, 89.2, 85.3, 53.2, 47.7, 36.0, 35.1, 26.0, 23.7, 18.1.

HRMS (ESI) m/z: [M+H] calculated 481.1646, found 481.1651

Dimerization of dehydro-α-lapachone with DIPEA:

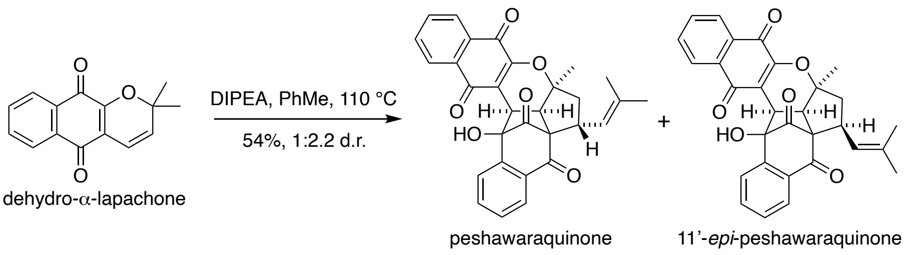

To a solution of dehydro-α-lapachone (2.50 g, 10.4 mmol) in PhMe (100 mL) was added *N*,*N*-diisopropylethylamine (DIPEA) (1.82 mL, 10.4 mmol) and the reaction mixture was heated at 110 °C for 18 h. This mixture was cooled to room temperature and then directly purified by flash chromatography on silica gel with CH_2Cl_2 as the eluent (prior evaporation of the PhMe solvent is unnecessary) to give a 1:2.2 mixture of peshawaraquinone and 11'-*epi*-peshawaraquinone (1.34 g, 54%). Recovered dehydro-α-lapachone starting material was also obtained (290 mg, 12%). Analytical samples of 11'-*epi*-peshawaraquinone were obtained by further flash chromatography on silica gel (1:1 hexanes-CH_2Cl_2 to neat CH_2Cl_2, gradient elution) or by preparative TLC with neat CH_2Cl_2 as the eluent. Single crystals of 11'-*epi*-peshawaraquinone were obtained by crystallization from PhMe.

One-step total synthesis of peshawaraquinone:

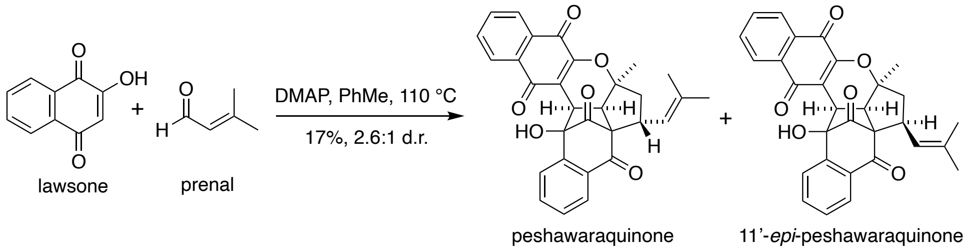

To a solution of lawsone (1.00 g, 5.74 mmol) and 4-dimethylaminopyridine (DMAP) (350 mg, 2.87 mmol) in PhMe (50 mL) was added prenal (0.66 mL, 6.89 mmol) and the reaction mixture was heated at 110 °C for 18 h. This mixture was cooled to room temperature and then directly purified by flash chromatography on silica gel with CH_2Cl_2 as the eluent (prior evaporation of the PhMe solvent is unnecessary) to give a 2.6:1 mixture of peshawaraquinone and 11'-*epi*-peshawaraquinone (230 mg, 17%). Pure peshawaraquinone was obtained (114 mg, 8%) by further flash chromatography on silica gel (1:1 hexanes-CH_2Cl_2 to neat CH_2Cl_2, gradient elution).

3. Dimerization Screening

General Methods

To a reaction vial containing a solution of dehydro-α-lapachone (1.0 eq.) in solvent was added base. The reaction was heated and stirred for the times and temperatures listed in the appropriate results tables for each variable screened (*vide infra*). Individual reactions were monitored by TLC using either pure CH_2Cl_2 or petroleum spirit 60-80 / ethyl acetate (3:1) as eluents. Visualization was aided by viewing under a UV lamp and staining with *p*-anisaldehyde stain followed by heating. Reactions which did not display product spots were discarded. Successful reactions had their solvents removed under reduced pressure or by work-up (see Solvent Screening for more detail). To the resulting crude mixtures, a known mass (approx. 44 mg or approx. 1.0 eq.) of benzyl benzoate for use as an internal NMR standard was added. These mixtures were then dissolved in $CDCl_3$ until homogenous after which, a small aliquot was submitted for ^{1}H NMR. Normalized integrals at H-12' (see NMR Assignments) were measured against the -CH_2 singlet of benzyl benzoate (5.37 ppm) to calculate crude NMR yields.

Base Screening. To a reaction vial containing a solution of dehydro-α-lapachone (50 mg, 0.21 mmol, 1.0 eq.) in PhMe (2 mL) was added base (0.21 mmol, 1.0 eq.). The reactions were heated at 110 °C and stirred between 1-5 h. PhMe was removed under reduced pressure.

Temperature Screening. To a reaction vial containing a solution of dehydro-α-lapachone (50 mg, 0.21 mmol, 1.0 eq.) in PhMe (2 mL) was added Et_3N, DIPEA or DMAP (0.21 mmol, 1.0 eq.). The reactions were stirred at room temperature, 50, 70, 90 and 110 °C between 4-25 h. PhMe was removed under reduced pressure.

Solvent Screening. To a reaction vial containing a solution of dehydro-α-lapachone (50 mg, 0.21 mmol, 1.0 eq) in solvent (2 mL) was added Et_3N, DIPEA or DMAP (0.21 mmol, 1.0 eq.). The reactions were stirred at either 80 or 100 °C between 4-21 h. Reactions in DMF or DMSO were diluted with water and extracted with EtOAc (3 x 10 mL). The organic layers were washed with H_2O (3 x 10 mL) and brine (1 x 10 mL), dried over anhydrous sodium sulfate, filtered and concentrated under reduced pressure. Reactions in H_2O were extracted with EtOAc (3 x 10 mL). The organic layer was dried over anhydrous sodium sulfate, filtered and concentrated under reduced pressure. All other solvents tested were removed under reduced pressure on a rotary evaporator, increasing the temperature of the water bath for higher boiling point solvents e.g., xylenes, 1,4-dioxane.

Base Equivalents Screening. To a reaction vial containing a solution of dehydro-α-lapachone (50 mg, 0.21 mmol, 1.0 eq.) in PhMe (2 mL) was added DIPEA or DMAP (0.2-1.5 eq.). The reactions were stirred at 110 °C between 4-25 h. PhMe was removed under reduced pressure.

Concentration Screening. To a reaction vial containing, dehydro-α-lapachone (1.0 eq.) was added PhMe (0.5-2 mL) to make up a solution of known concentration (0.1 M – 2.0 M). To these solutions was added DIPEA or DMAP (1.0 eq.). The reactions were stirred at 110 °C for 4 h. PhMe was removed under reduced pressure.

Reaction Time Screening. To a reaction vial containing a solution of dehydro-α -lapachone (50 mg, 0.21 mmol, 1.0 eq.) in PhMe (2 mL) was added DIPEA or DMAP (0.10 mmol, 0.5 eq.). The reactions were stirred at 70 °C between 6-96 h. PhMe was removed under reduced pressure.

Base Screening

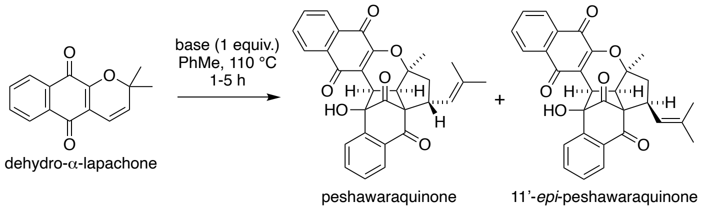

Entry	Base	Solvent	Temp. (°C)	Time (h)	% Dehydro-α-lapachone	% Peshawaraquinone	% 11′-Peshawaraquinone
1	-	PhMe	110	23	54.1	2.2	19.6
2	Et_3N	PhMe	110	4	7.7	13.6	22.8
3	*i*-$PrNH_2$	PhMe	110	4	9.2	9.6	23.2
4	DIPA	PhMe	110	4	9.9	14.2	20.8
5	Piperidine	PhMe	110	4		Decomposition	
6	DBU	PhMe	110	4		Decomposition	
7	DABCO	PhMe	110	4	3.5	10.4	15.0
8	DIPEA	PhMe	110	5	29.2	12.6	33.8
9	*t*-BuOK	1,4-dioxane	100	1		Decomposition	
10	NaOMe	MeOH	65	5		Decomposition	
11	DMAP	PhMe	110	4	5.1	24.8	23.8
12	Pyridine	PhMe	110	4	73.5	1.4	9.6
13	MTBD	PhMe	110	1		Decomposition	

Temperature Screening

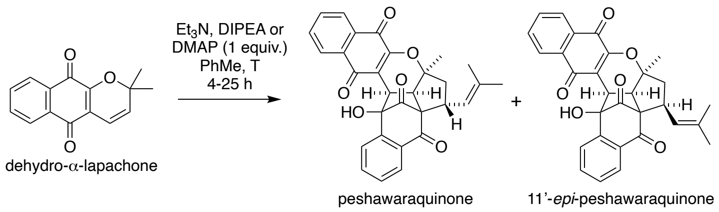

Entry	Base	Temp. (°C)	Time (h)	% Dehydro-α-lapachone	% Peshawaraquinone	% 11′-Peshawaraquinone
1	Et_3N	rt	25	74.2	1.8	3.4
2		50	19	22.0	13.6	24.2
3		70	19	7.1	16.6	32.2
4		90	4	18.0	16.6	32.8
5		110	4	10.5	14.4	29.0
6	DIPEA	rt	25	87.5	0	0
7		50	19	74.6	5.2	9.6
8		70	19	46.7	9.2	22.6
9		90	4	51.6	8.4	19.8
10		110	4	36.8	10.0	26.4
11	DMAP	rt	25	74.6	2.6	0
12		50	19	9.8	20.6	13.6
13		70	19	5.0	28.4	14.6
14		90	4	8.9	32.4	20.6
15		110	4	6.1	31.4	20.8

Solvent Screening

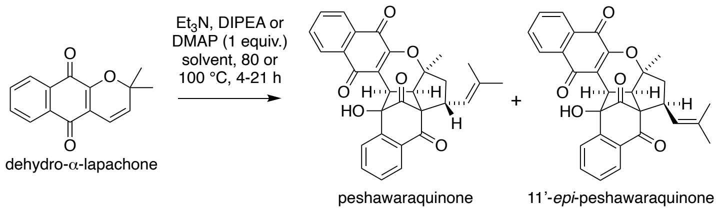

Entry	Base	Solvent	Temp. (°C)	Time (h)	% Dehydro-α-lapachone	% Peshawaraquinone	% 11′-Peshawarquinone
1	DIPEA	Xylenes	100	4	44.4	8.4	22.8
2		DMF	100	4	0	5.6	4.2
3		DMSO	100	4	1.2	5.0	4.6
4		H_2O	100	4	2.2	18.8	7.8
5		1,4-dioxane	100	4	14.3	6.8	45.2
6		1,2-dichloroethane	80	18	4.4	33.8	18.2
7		MeCN	80	18	2.5	23.6	16.6
8		Benzene	80	18	19.1	21.2	32.6
9		Cyclohexane	80	18	69.0	5.0	11.0
10		EtOH	80	18	1.8	20.8	10.8
11	DMAP	Xylenes	100	4	7.4	23.8	12.8
12		DMF	100	4	1.5	13.6	9.0
13		DMSO	100	4	0	9.6	6.8
14		H_2O	100	4	2.3	9.8	4.4
15		1,4-dioxane	100	4	5.5	15.2	22.8
16		1,2-dichloroethane	80	21	6.6	24.8	16.0
17		MeCN	80	21	0	16.8	14.2
18		Benzene	80	21	3.6	30.4	17.6
19		Cyclohexane	80	21	0	0	0
20		EtOH	80	21	0	0	0
21	Et_3N	H_2O	100	4	2.7	16.0	8.0

Base Equivalents Screening

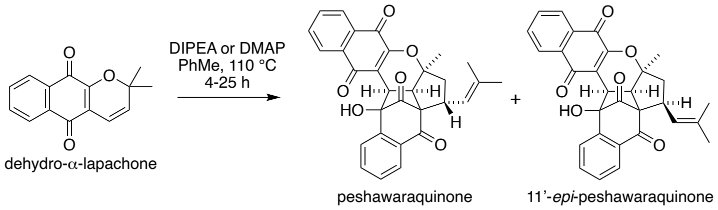

Entry	Base	Equivalents	Time (h)	% Dehydro-α-lapachone	% Peshawaraquinone	% 11′-Peshawaraquinone
1	DIPEA	0.2	23	20.5	13.8	35.6
2		0.5	23	9.8	14.0	31.4
3		0.8	23	10.3	19.2	32.8
4		1.2	4	33.3	13.2	25.8
5		1.5	4	37.0	7.8	18.6
6	DMAP	0.2	25	7.6	17.6	16.4
7		0.5	25	6.4	26.8	18.6
8		0.8	25	3.8	24.0	15.6
9		1.2	4	4.5	18.4	12.0
10		1.5	4	5.8	17.4	10.8

Concentration Screening

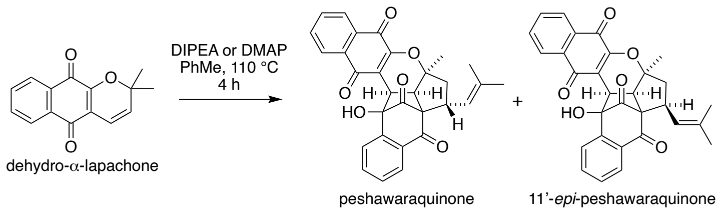

Entry	Base	Concentration (M)	Time (h)	% Dehydro-α-lapachone	% Peshawaraquinone	% 11′-Peshawaraquinone
1	DIPEA	0.1	4	50.1	16.0	30.8
2		0.5	4	3.6	20.0	22.8
3		1.0	4	2.4	10.4	12.0
4		1.5	4	1.0	4.6	5.2
5		2.0	4	1.7	6.0	7.0
6	DMAP	0.1	4	6.7	19.8	14.0
7		0.5	4	0	8.4	8.2
8		1.0	4	0	0	0
9		1.5	4	0	0	0
10		2.0	4	0	0	0

Reaction Time Screening

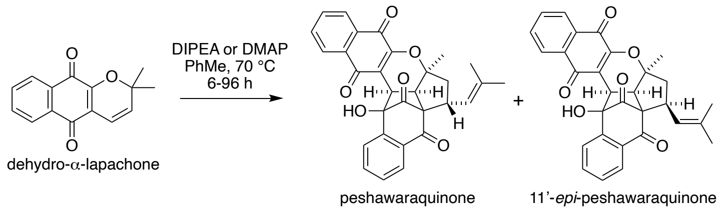

Entry	Base	Time (h)	% Dehydro-α-lapachone	% Peshawaraquinone	% 11′-Peshawaraquinone
1	DIPEA	6	80.1	0	0
2		24	56.6	9.8	21.2
3		48	29.0	12.0	33.2
4		72	27.9	15.6	40.0
5		96	13.6	16.6	41.2
6	DMAP	6	39.6	18.0	0
7		24	10.1	28.8	15.0
8		48	3.1	29.4	14.6
9		72	4.8	32.0	16.6
10		96	2.9	32.0	15.0

4. NMR Spectra

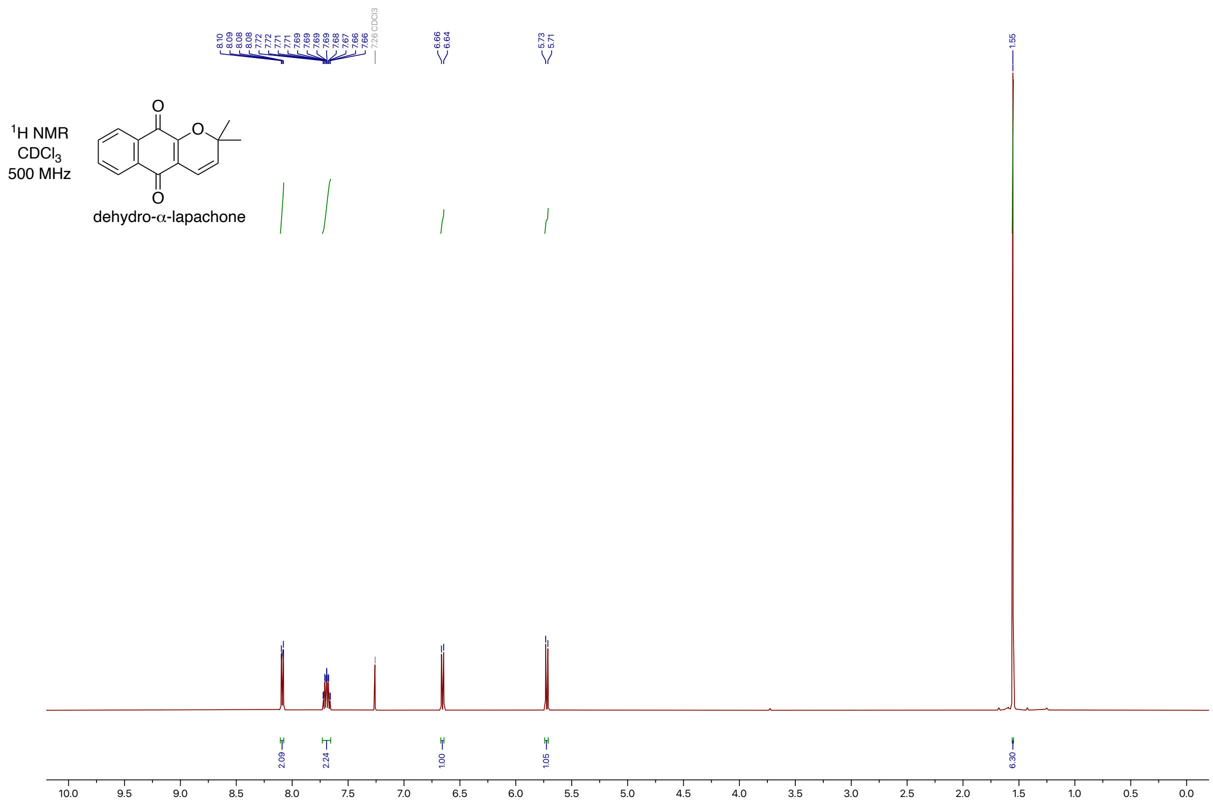

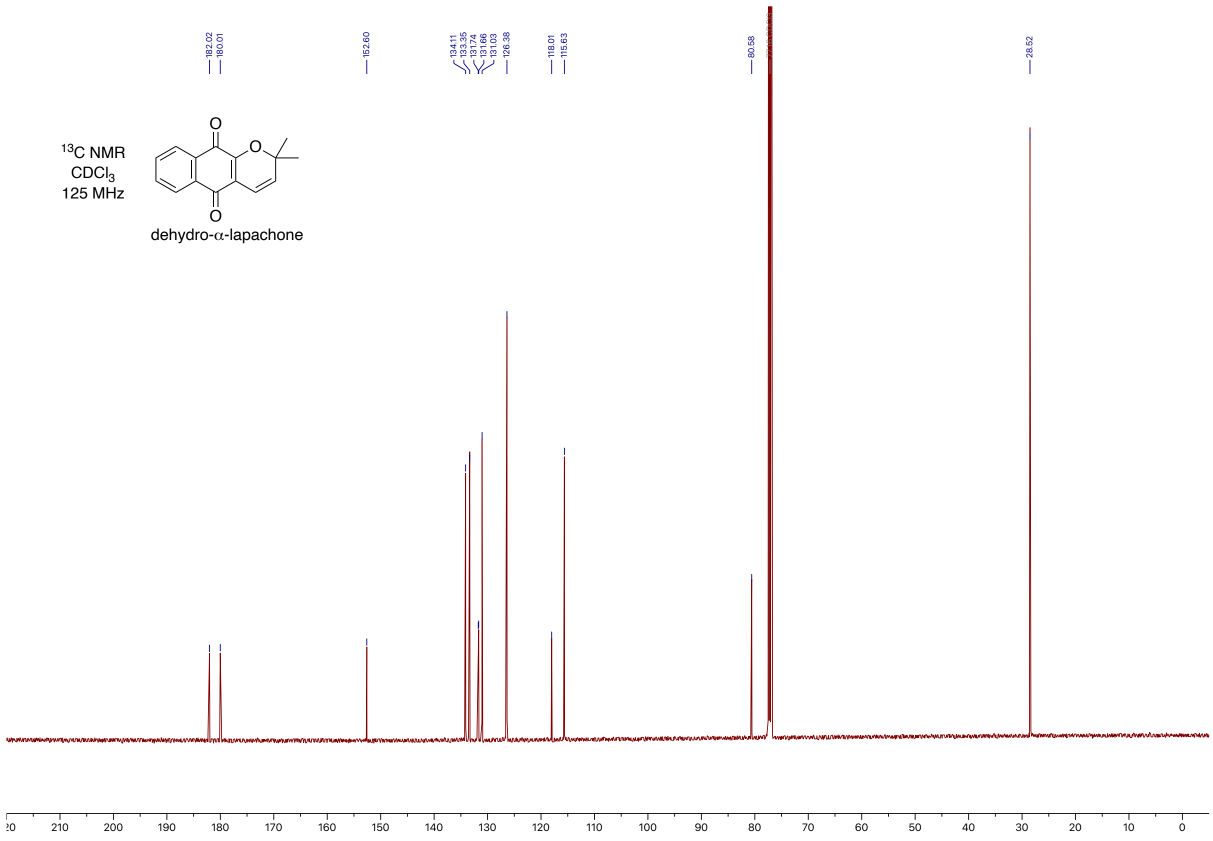

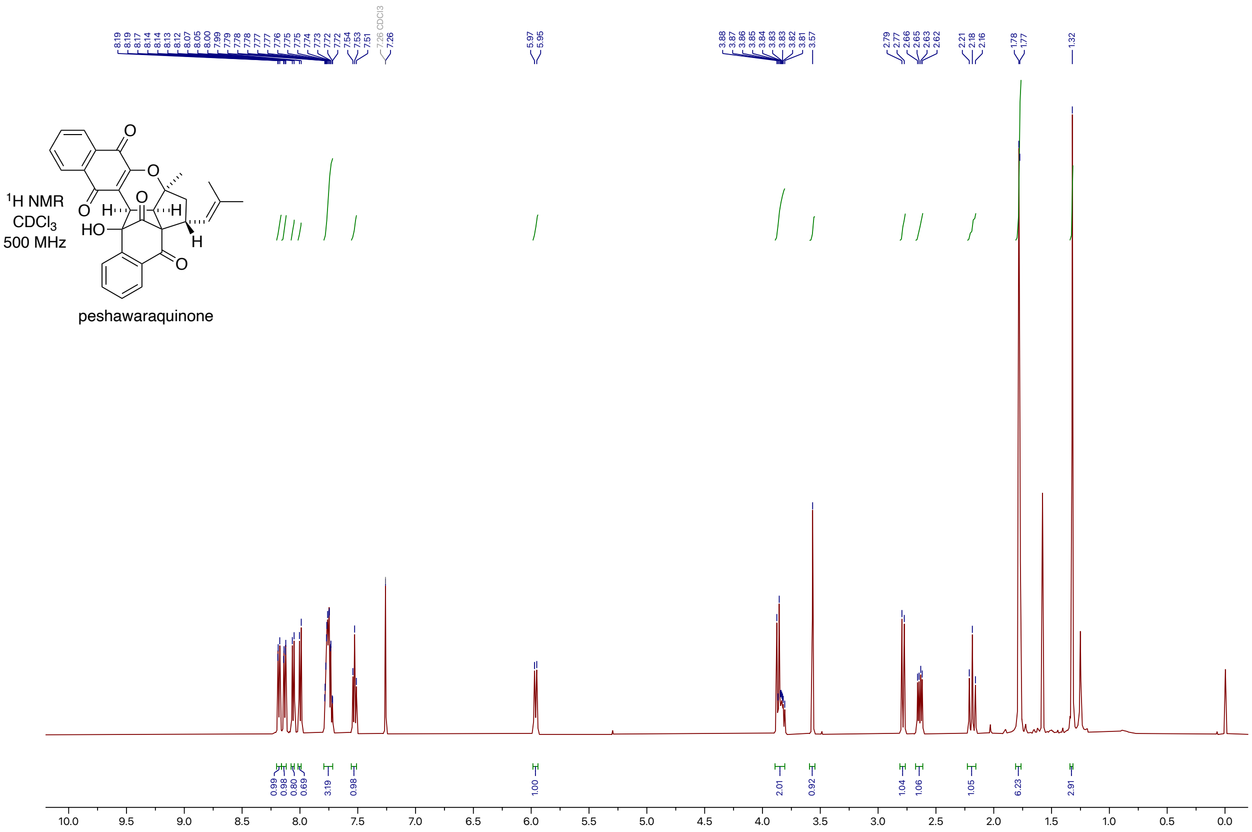

1H NMR
CDCl3
500 MHz
peshawaraquinone
8.19 8.19 8.17 8.14 8.14 8.13 8.12 8.07 8.05 8.00 7.99 7.79 7.78 7.78 7.77 7.77 7.76 7.75 7.75 7.74 7.73 7.72 7.72 7.54 7.53 7.51 7.26 CDCl3 7.26
5.97 5.95
3.88 3.87 3.86 3.85 3.84 3.83 3.83 3.82 3.81 3.57
2.79 2.77 2.66 2.65 2.63 2.62 2.21 2.18 2.16 1.78 1.77 1.32
0.99 0.98 0.80 0.69 3.19 0.98 1.00 2.01 0.92 1.04 1.06 1.05 6.23 2.91
10.0 9.5 9.0 8.5 8.0 7.5 7.0 6.5 6.0 5.5 5.0 4.5 4.0 3.5 3.0 2.5 2.0 1.5 1.0 0.5 0.0

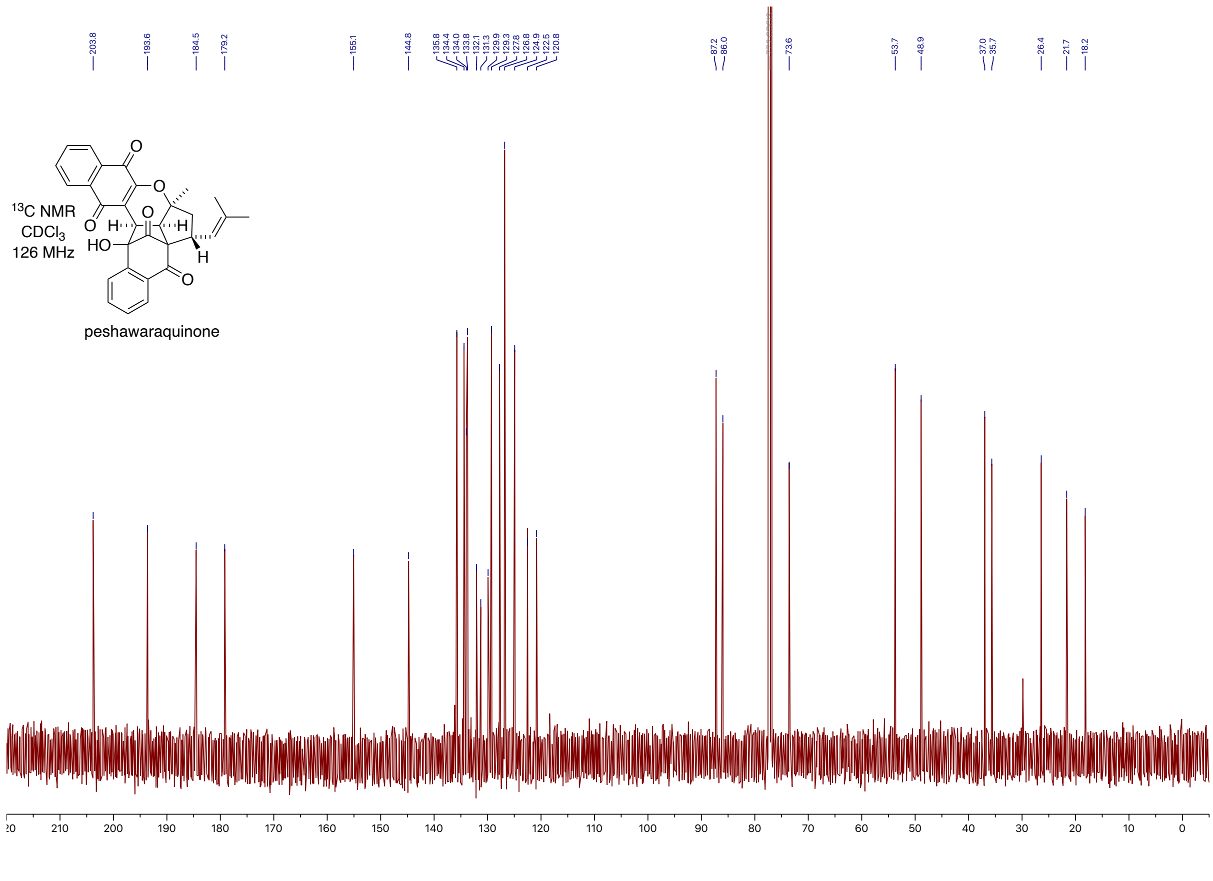

13C NMR
CDCl3
126 MHz
peshawaraquinone
203.8 193.6 184.5 179.2 155.1 144.8
135.8 134.4 134.0 133.8 132.1 131.3 129.9 129.3 127.8 126.8 124.9 122.5 120.8
87.2 86.0 73.6 53.7 48.9 37.0 35.7 26.4 21.7 18.2
210 200 190 180 170 160 150 140 130 120 110 100 90 80 70 60 50 40 30 20 10 0

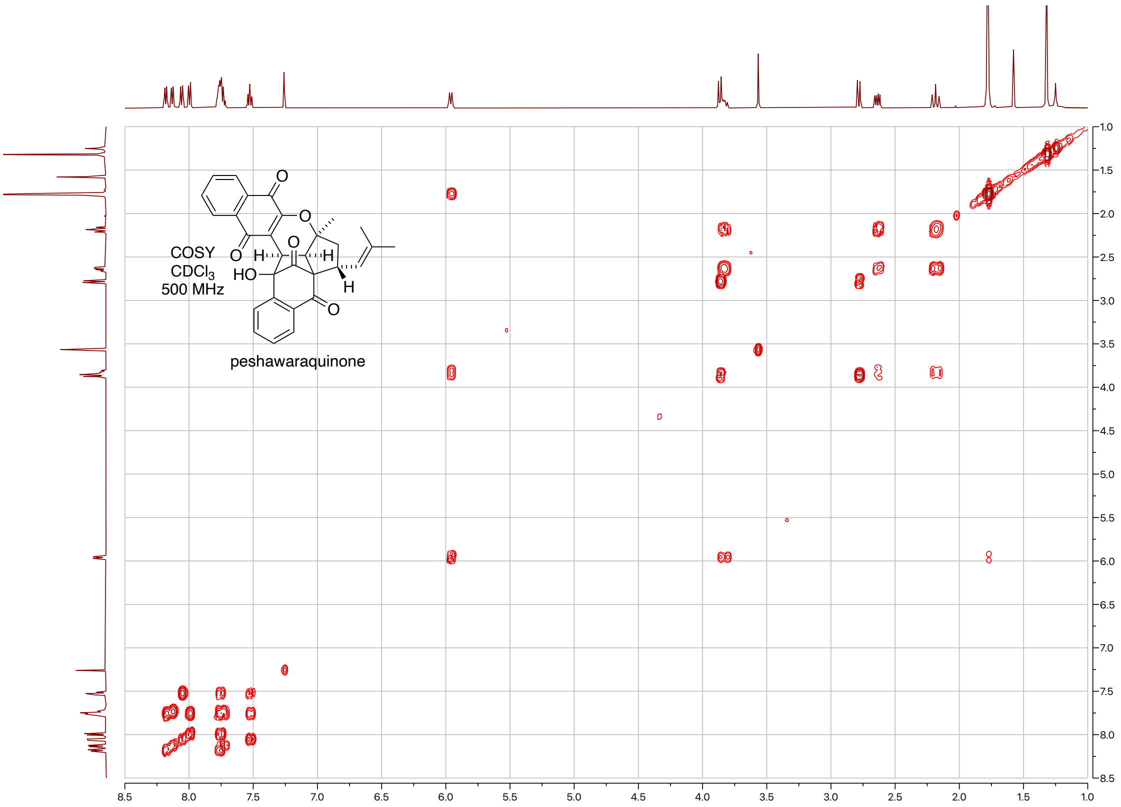
COSY
CDCl3
500 MHz
peshawaraquinone

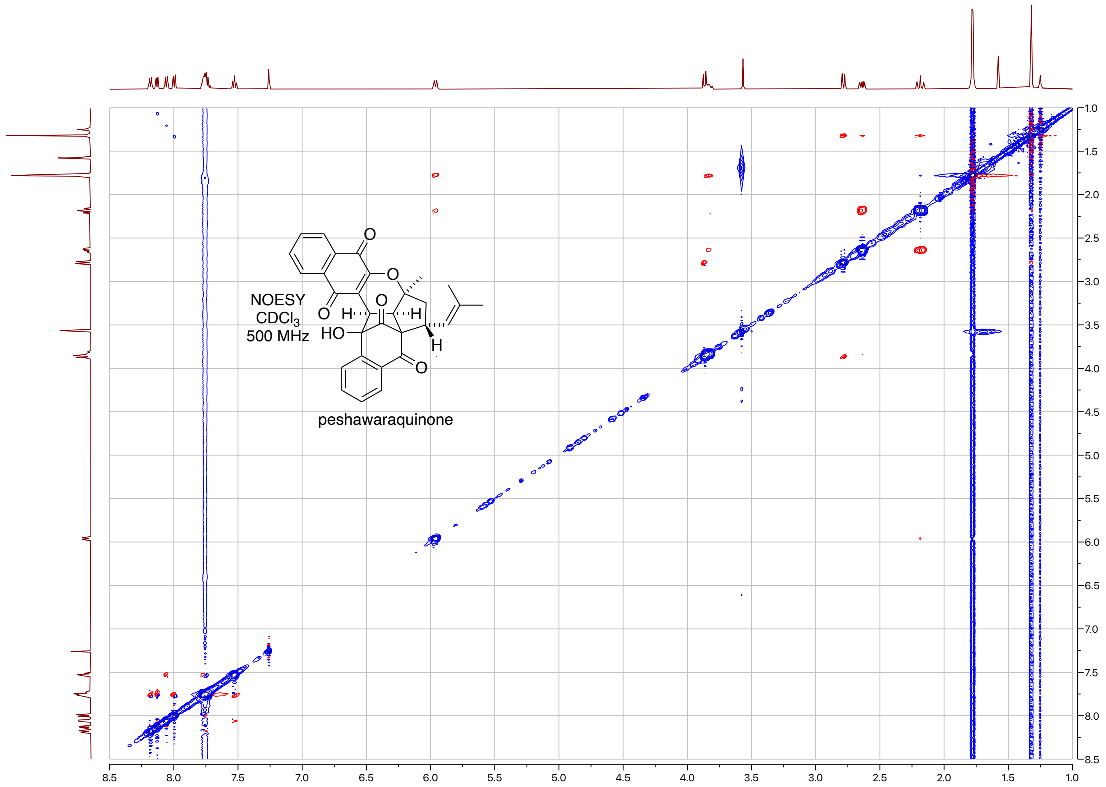
NOESY
CDCl3
500 MHz
peshawaraquinone

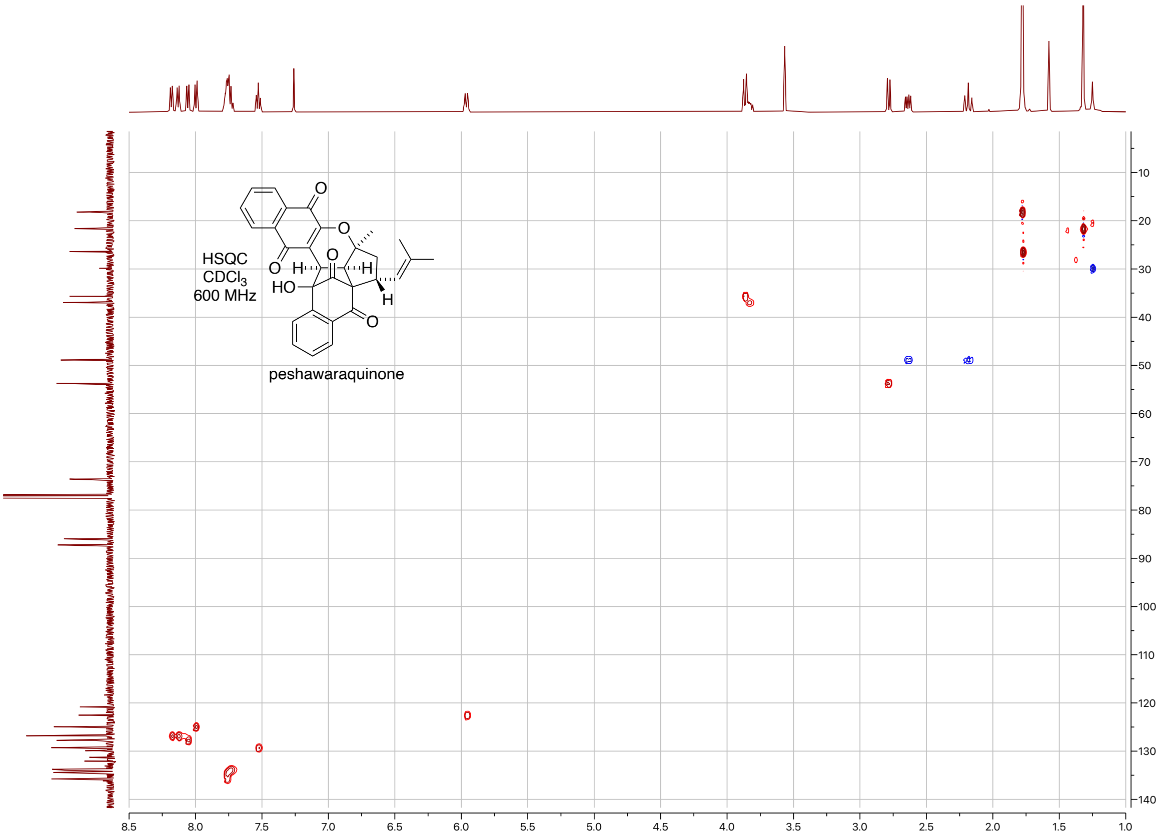
HSQC
CDCl3
600 MHz
peshawaraquinone

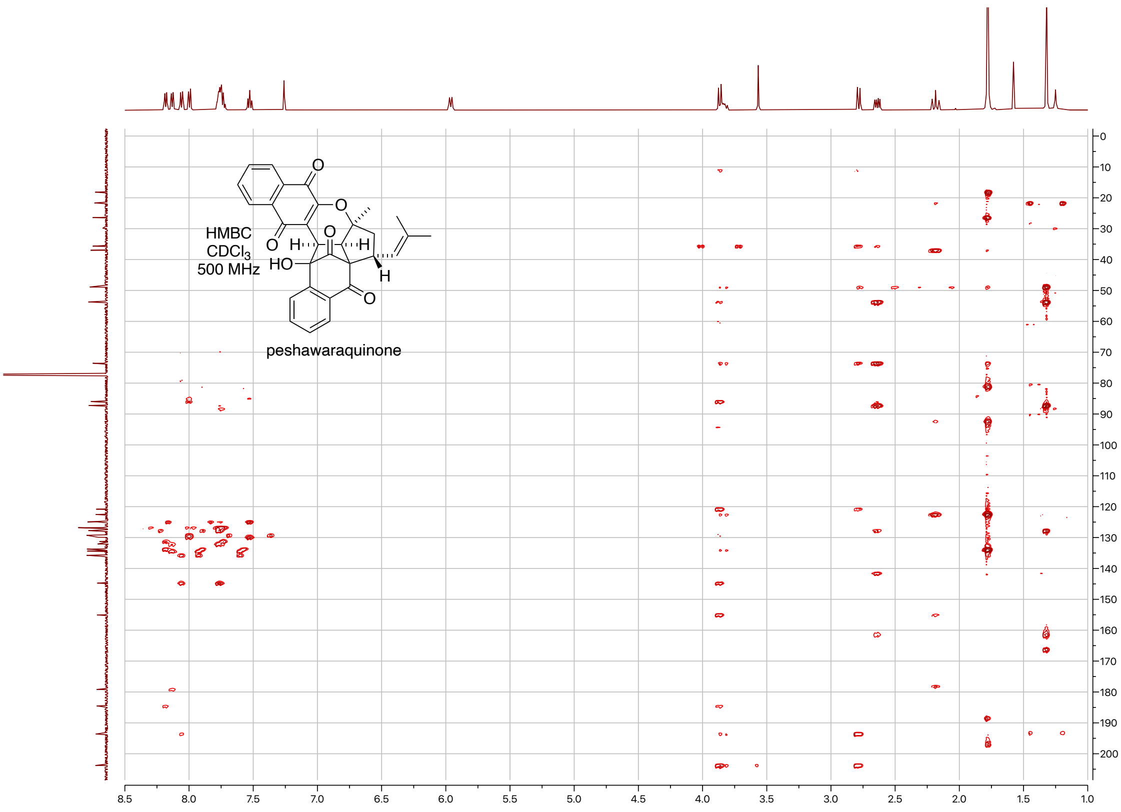
HMBC
CDCl3
500 MHz
peshawaraquinone

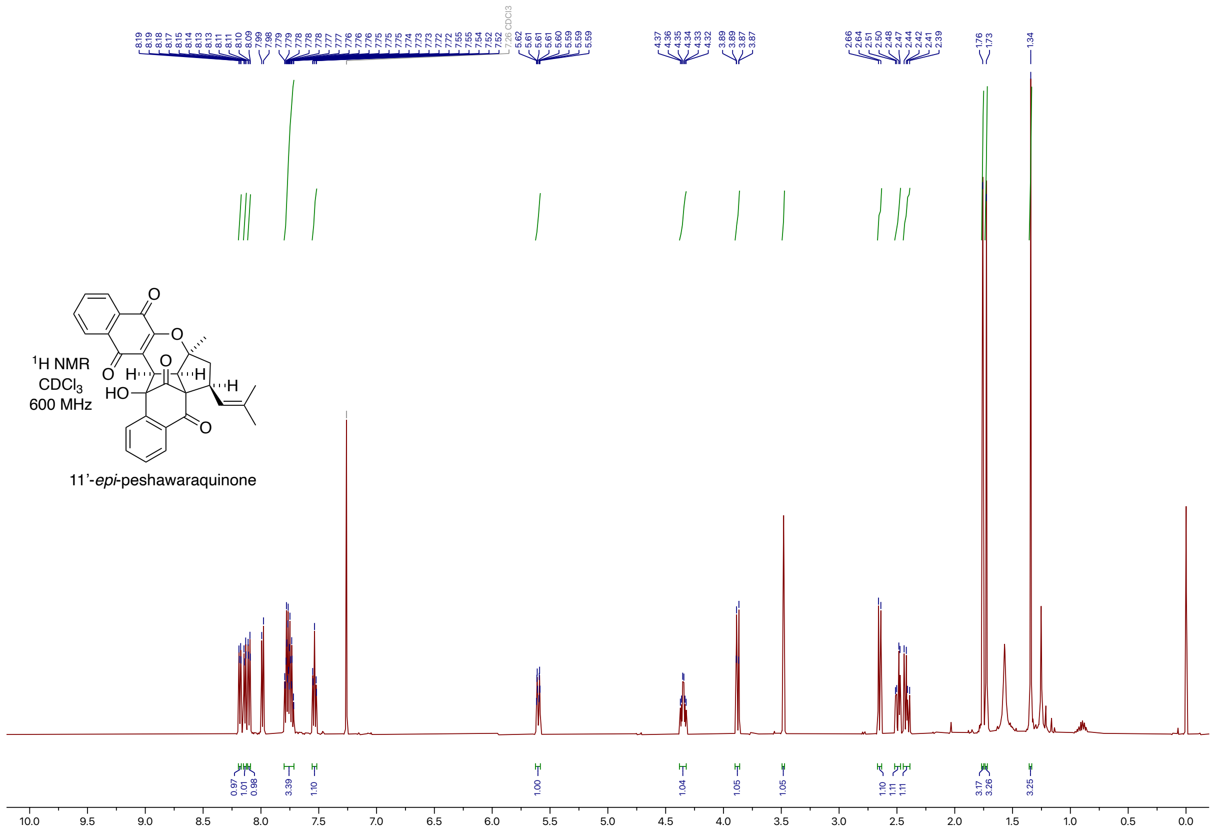

^{1}H NMR
CDCl$_3$
600 MHz
11'-*epi*-peshawaraquinone

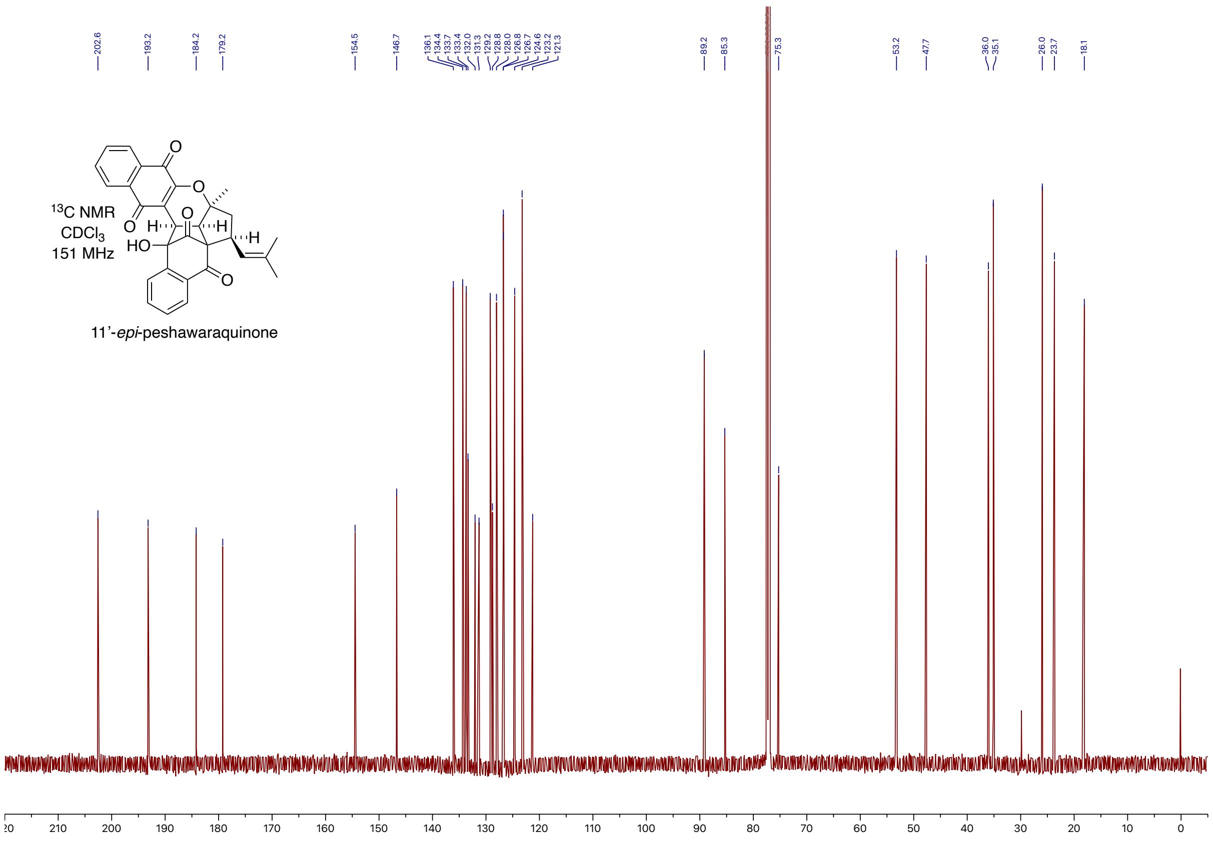

^{13}C NMR
CDCl$_3$
151 MHz
11'-*epi*-peshawaraquinone

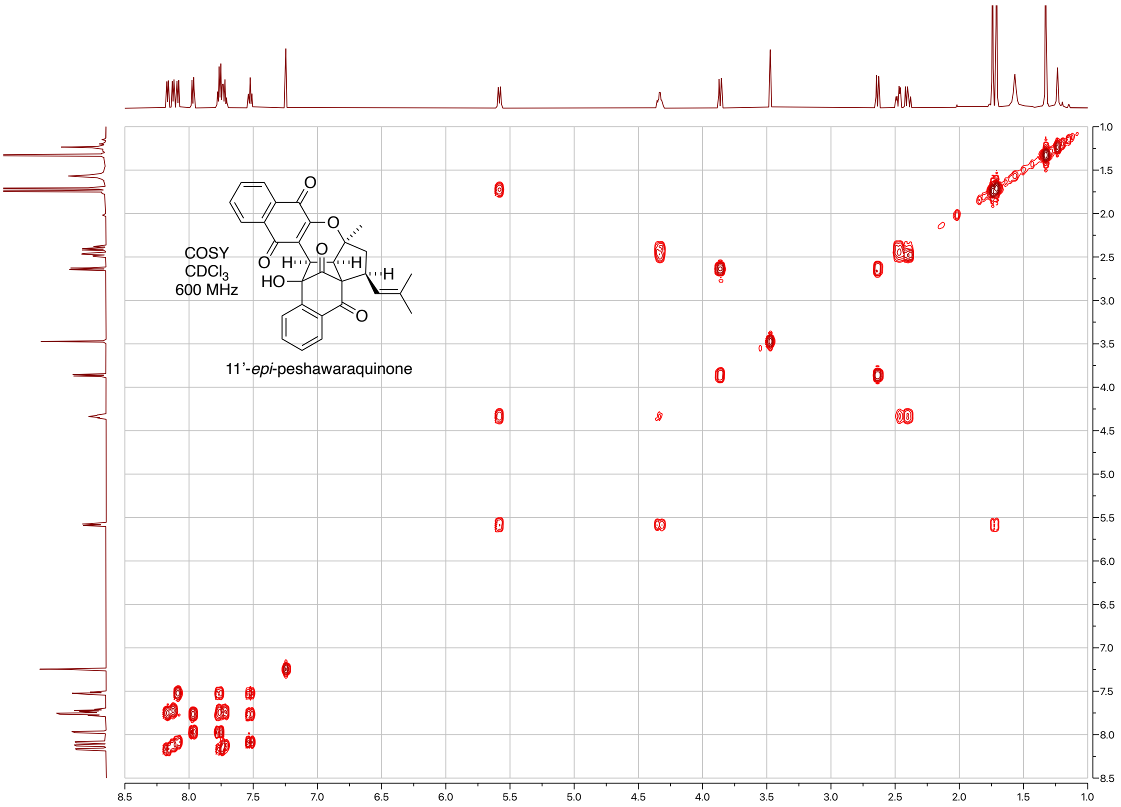
COSY
$CDCl_3$
600 MHz
11'-*epi*-peshawaraquinone

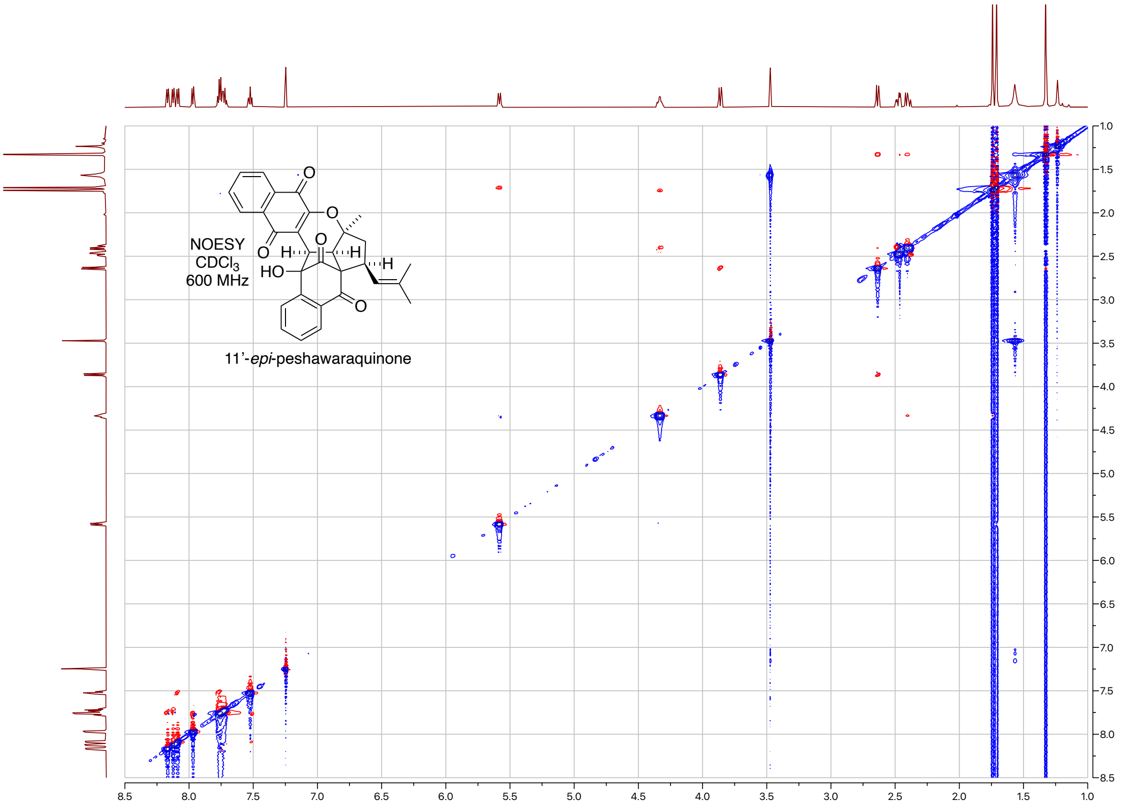
NOESY
$CDCl_3$
600 MHz
11'-*epi*-peshawaraquinone

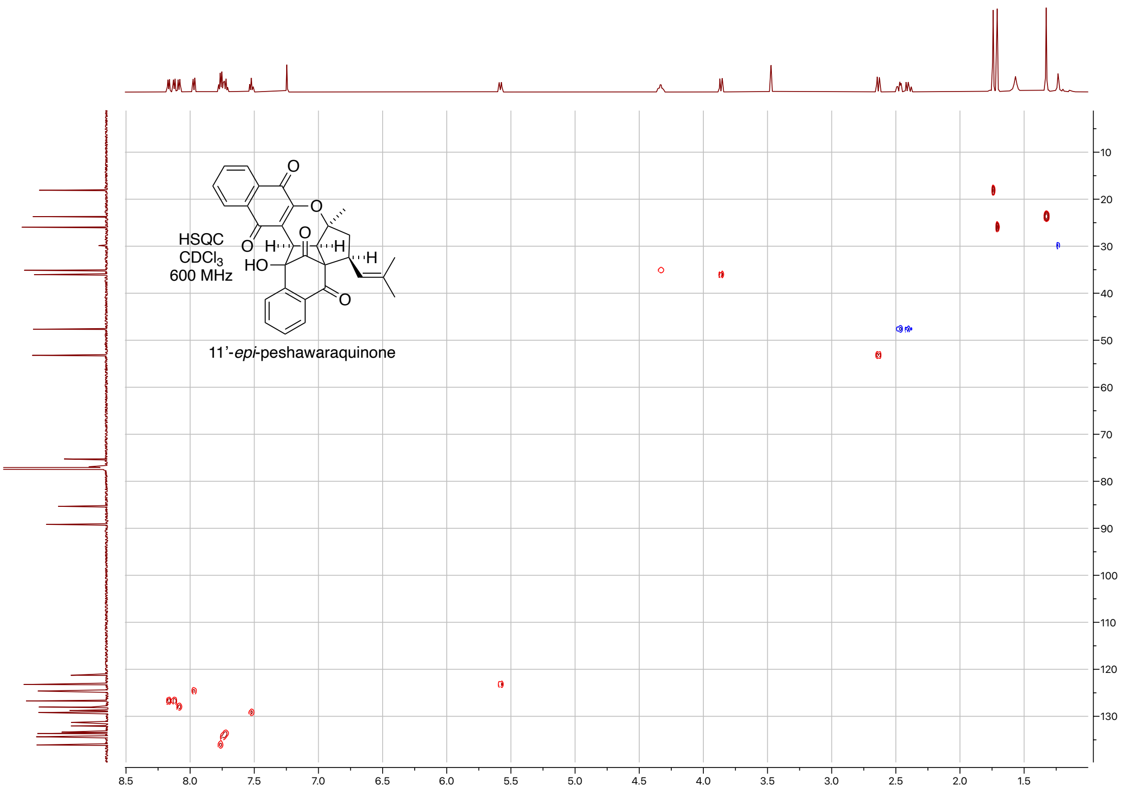
HSQC
CDCl3
600 MHz
11'-epi-peshawaraquinone

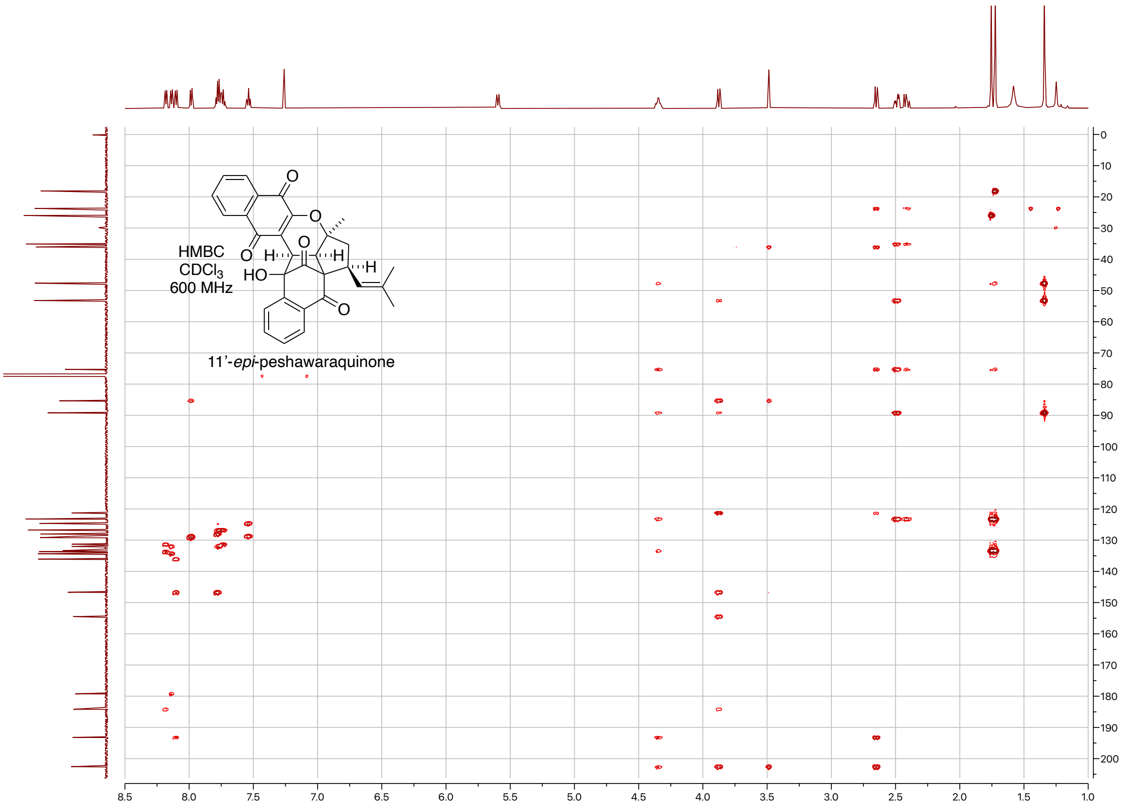
HMBC
CDCl3
600 MHz
11'-epi-peshawaraquinone

Crude ^{1}H NMR of the dimerization of dehydro-α-lapachone (DHL) with DIPEA in PhMe at 110 °C after 18 h

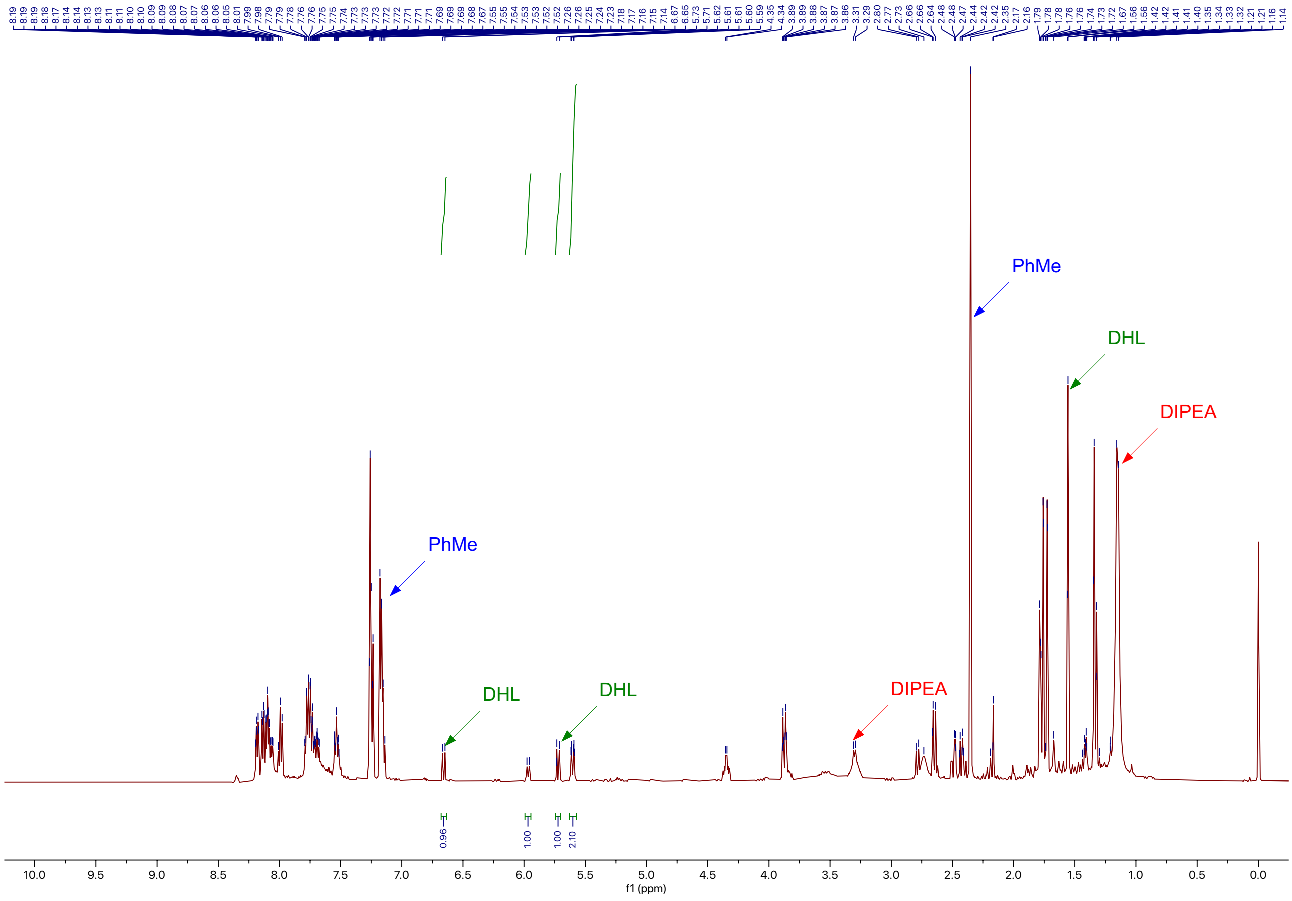

Crude NMR shows a 2.1:1:1 mixture of 11'-*epi*-peshawaraquinone : peshawaraquinone : dehydro-α-lapahone. This indicates 76% conversion of dehydro-α-lapahone to the dimeric products. There are no significant by-products observed during the reaction.

5. NMR Assignments

NMR assignment for peshawaraquinone:

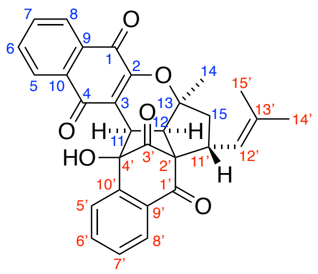

Assignment	Isolated sample, $CDCl_3$		Synthetic sample, $CDCl_3$	
	^{1}H, 400 MHz (δ)	^{13}C, 100 MHz (δ)	^{1}H, 500 MHz (δ)	^{13}C, 126 MHz (δ)
1	-	184.4	-	184.5
2	-	120.7	-	120.8
3	-	154.9	-	155.1
4	-	179.3	-	179.2
5	8.12 (dd, 8.4, 1.6)	126.7	8.13 (d, 7.7)	126.8
6	7.74 (m)	133.6	7.73 (m, overlapped)	133.8
7	7.74 (m)	134.3	7.75 (m, overlapped)	134.4
8	8.16 (dd, 8.4, 1.6)	126.7	8.18 (d, 6.8)	126.8
9	-	131.9	-	132.1
10	-	131.1	-	131.1
11	3.82 (d, 10.4)	35.5	3.87 (d, 10.4)	35.7
12	2.76 (d, 10.4)	53.6	2.78 (d, 10.2)	53.7
13	-	87.1	-	87.2
14	1.30 (s)	21.5	1.32 (s)	21.7
15	2.62 (dd, 13.2, 6.4)<br>2.17 (t, 13.2)	48.7	2.64 (dd, 13.6, 6.4)<br>2.18 (t, 13.2)	48.9
1’	-	193.5	-	193.6
2’	-	73.4	-	73.6
3’	-	203.6	-	203.8
4’	-	85.8	-	86.0
5’	7.92 (dd, 8.0, 1.5)	124.8	8.00 (d, 7.8)	124.9
6’	7.74 (m, overlapped)	135.5	7.75 (m, overlapped)	135.8
7’	7.51 (m, overlapped)	129.1	7.53 (t, 7.5)	129.3
8’	8.04 (dd, 7.6, 1.5)	127.6	8.06 (d, 7.8)	127.8
9’	-	129.7	-	129.9
10’	-	144.6	-	144.8
11’	3.78 (m)	36.8	3.83 (m)	37.0
12’	5.94 (d, 9.6)	122.4	5.96 (d, 9.7)	122.5
13’	-	133.9	-	134.0
14’	1.76 (s)	18.1	1.78 (s)	18.2
15’	1.75 (s)	26.3	1.77 (s)	26.4
4’-OH	3.54 (s)	-	3.57 (s)	-

NMR assignment for 11'-*epi*-peshawaraquinone:

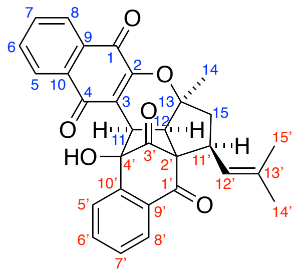

11'-*epi*-peshawaraquinone

Assignment	Synthetic sample, $CDCl_3$	
	^{1}H, 600 MHz (δ)	^{13}C, 151 MHz (δ)
1	-	184.2
2	-	121.3
3	-	154.5
4	-	179.2
5	8.14 (d, 7.4)	126.8
6	7.73 (m, overlapped)	133.7
7	7.74 (m, overlapped)	134.4
8	8.18 (d, 7.4)	126.7
9	-	132.0
10	-	131.3
11	3.88 (d, 10.1)	36.0
12	2.65 (d, 10.2)	53.2
13	-	89.2
14	1.34 (s)	23.7
15	2.49 (dd, 14.4, 5.1)<br>2.41 (dd, 14.4, 9.6)	47.7
1'	-	193.2
2'	-	75.3
3'	-	202.6
4'	-	85.3
5'	7.99 (d, 7.8)	124.6
6'	7.76 (m, overlapped)	136.1
7'	7.54 (t, 7.7)	129.2
8'	8.10 (d, 7.8)	128.0
9'	-	128.8
10'	-	146.7
11'	4.35 (td, 9.9, 5.1)	35.1
12'	5.60 (d, 10.3)	123.2
13'	-	133.4
14'	1.76 (s)	18.1
15'	1.73 (s)	26.0
4'-OH	3.48 (s)	-

6. Single Crystal X-ray Crystallography

A single crystal of 11'-*epi*-peshawaraquinone (obtained by slow evaporation of a solution in toluene) was mounted in Paratone-N oil on a MiTeGen micromount. X-ray diffraction data were collected at 100(2) K on a Rigaku-Oxford Diffraction Synergy single crystal diffractometer using Cu Kα radiation.[3] The data set was corrected for absorption using a multi-scan method, and the structure solved by intrinsic phasing (SHELXT)[4] and refined by full-matrix least squares on F2 by SHELXL,[5] interfaced through the programs X-Seed (version 4)[6] and Olex2.3.[7] All non-hydrogen atoms were refined anisotropically and hydrogen atoms were included as invariants at geometrically estimated positions. Disordered solvent (toluene) was modelled over two positions using EXYZ, EADP, SIMU and RIGU restraints.

Table S1 lists the X-ray experimental data and refinement parameters for the crystal structures. Perspective views of the structure of 11'-*epi*-peshawaraquinone are shown in Figure S1. Full details of the structure determination have been deposited with the Cambridge Crystallographic Data Centre as CSD 2179170. Copies of this information may be obtained free of charge from The Director, CCDC, 12 Union Street, Cambridge CB2 1EZ, U.K. (fax, +44-1223-336-033; e-mail, deposit@ccdc.cam.ac.uk).

Table S1. X-ray experimental data for 11'-*epi*-peshawaraquinone.

Compound	11'-*epi*-peshawaraquinone
CCDC number	2179170
cif	**1jg1r_auto**
Empirical formula	$C_{33.5}H_{28}O_6$
Formula weight	526.56
Crystal system	monoclinic
Space group	$P2_1/n$
a (Å)	17.6590(2)
b (Å)	8.38590(10)
c (Å)	17.8047(2)
α (°)	90
β (°)	92.6800(10)
γ (°)	90
Volume (Å^3)	2633.75(5)
Z	4
Density (calc.) (Mg/m^3)	1.328
Absorption coefficient (mm^{-1})	0.737
F(000)	1108.0
Crystal size (mm^3)	0.216 × 0.05 × 0.026
2θ range for data collection (°)	6.89 to 154.332
Reflections collected	25450
Independent reflections	5234 [R_{int} = 0.0345, R_{sigma} = 0.0276]
Data/restraints/parameters	5234/57/366
Goodness-of-fit on F^2	1.008
Final R indexes [I>=2σ (I)]	R_1 = 0.0572, wR_2 = 0.1490
Final R indexes [all data]	R_1 = 0.0626, wR_2 = 0.1535
Largest diff. peak/hole / e Å^{-3}	0.88/-0.38

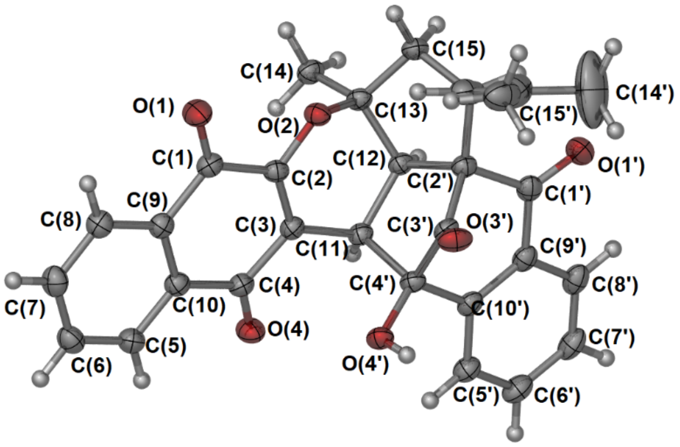

(a)

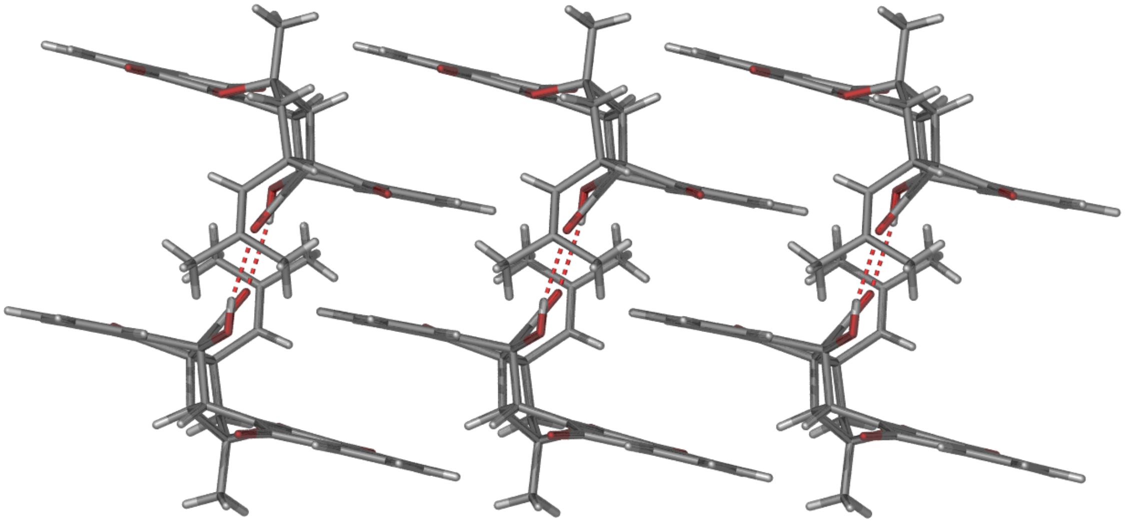

(b)

Figure S1. Perspective views of (a) the partially labelled asymmetric unit, without the toluene solvate molecule, with the ellipsoids shown at 50% probability level, and (b) the interdigitated hydrogen bonded dimers, shown as rods, which are directed along the crystallographic *b* axis, in the structure of 11'-*epi*-peshawaraquinone. Carbon – grey, hydrogen – white, oxygen – red. The intermolecular hydrogen bonding parameters are: $D_{O1\text{-}H1\cdots O1AA}$ = 2.87 Å, $angle_{O1\text{-}H1\cdots O1AA}$ = 168.7° and the centroid-centroid distance from the offset stacked phenyl and phenyl rings (central interaction) and quinone and phenyl rings (outer interactions) between two dimers are 3.73 and 3.96 Å respectively.

7. Computational Methods

Molecular geometries and energies were calculated by density function theory (DFT) using the ORCA quantum chemistry software package (version 5.0.3.).[8] Geometries were optimized in the gas phase using the ωB97X-D3 functional[9] and def2-SVP basis set.[10] Optimized molecular geometries are given below. Single-point energy calculations ($E_{\omega B97X\text{-}D3/def2\text{-}TZVPD,toluene}$) of the optimized geometries were also carried out using the same functional with the def2-TZVPD basis set[11] and the SMD continuum solvent model[12] with toluene as the solvent. The experimental X-ray crystal structure of 11'-*epi*-peshawaraquinone was used as the starting point for the geometry optimization of this molecule, while the same X-ray structure with stereochemistry inverted at the appropriate position was used as the starting point for the geometry optimization of peshawaraquinone. The global minimum-energy conformer from a conformer search with a metadynamics-based algorithm[13] and semiempirical tight binding at the GFN2-xTB level,[14] conducted using xtb[15] and the Conformer–Rotamer Ensemble Sampling Tool (CREST)[16] with default parameters, was used as a starting point for the DFT geometry optimization of structures **dehydro-α-lapachone (0)**, **1**, **3**, and **3'**. The transition state connecting each pair of stable states was obtained using the NEB-TS method,[17] which combines the climbing image nudged elastic band (CI-NEB) method with an eigenvector following optimization starting from the climbing image, with the default parameters in ORCA. Stable states and transition states were identified by the number of imaginary vibrational frequencies (0 and 1, respectively) obtained from a harmonic frequency calculation at the ωB97X-D3/def2-SVP level. The thermal correction to the Gibbs free energy ($G_{thermal,\omega B97X\text{-}D3/def2\text{-}SVP}$) was calculated at 298.15 K and 1 atm using unscaled harmonic frequencies. The total Gibbs free energy in toluene solution was calculated as $G_{soln} = E_{\omega B97X\text{-}D3/def2\text{-}TZVPD,toluene} + G_{thermal,\omega B97X\text{-}D3/def2\text{-}SVP}$.

A summary of the calculated energies and free energies is given in Table S2, and reaction free-energy profiles for the conversion of **dehydro-α-lapachone (0)** to **1**, **3** to **5**, and **3'** to **5'** are shown in Figures S2, S3, and S4, respectively. The minimum-energy pathway from the global-minimum energy conformer of **3** to **5** was found to involve interconversion of several different conformers of **3**, with the most direct pathway being much higher in energy. Similarly, the minimum-energy pathway from the global-minimum energy conformer of **3'** to **5'**, which consists of an analogous series of steps, was found to involve interconversion of several different conformers of **3'**.

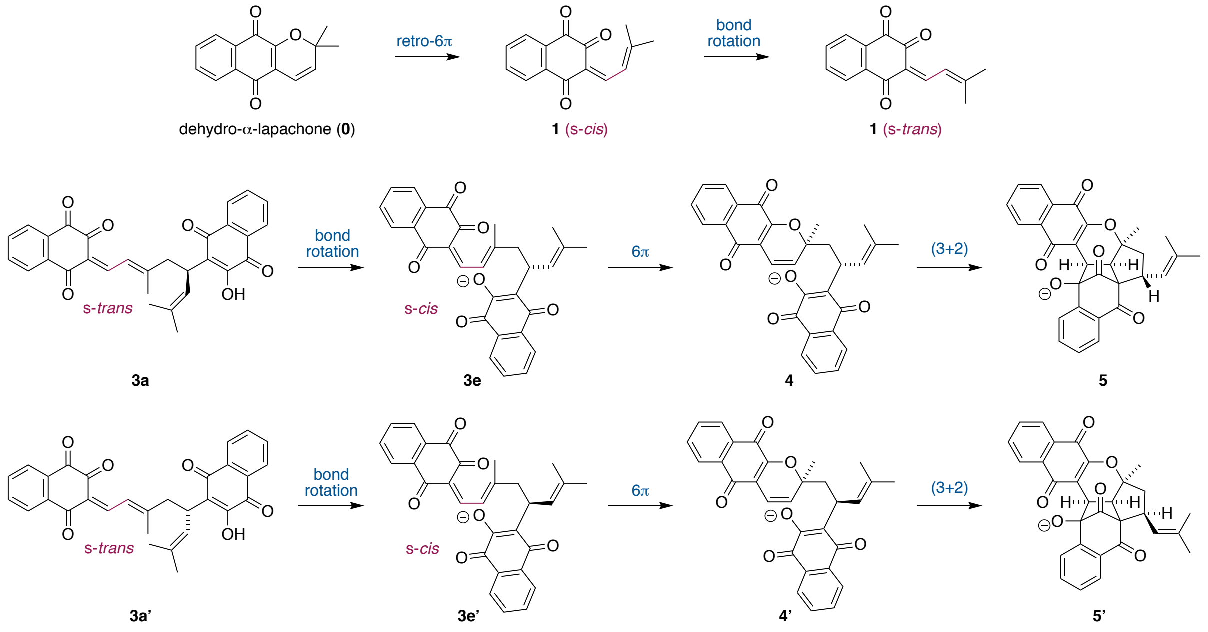

Scheme S1. Summary of the reactions modelled.

Table S2. Calculations summary.

structure	electronic energy [ωB97X-D3/ def2-SVP] (a.u.)	electronic energy + solvation free energy @ 298.15 K [SMD (toluene)/ ωB97X-D3/ def2-TZVPD] (a.u)	Thermal Gibbs free energy correction @ 298.15 K, 1 atm [ωB97X-D3/ def2-SVP] (a.u.)	Total Gibbs free energy (toluene) @ 298.15 K, 1 atm (a.u.)	Relative Gibbs free energy (toluene) @ 298.15 K, 1 mol/L (kJ/mol)
**0**	-803.68633248	-804.57639290	0.19632211	-804.38007079	0.0
**1 (s-*trans*)**	-803.66728503	-804.56073807	0.19234204	-804.36839603	30.7
**1 (s-*cis*)**	-803.65853504	-804.55083959	0.19303693	-804.35780266	58.5
**TS 0 → 1 (s-*cis*)**	-803.64239070	-804.53451856	0.19342264	-804.34109592	102.3
**TS 1 (s-*cis*) → 1 (s-*trans*)**	-803.65266694	-804.54642524	0.19238656	-804.35403868	68.3
**3a**	-1606.84576748	-1608.65034553	0.40402017	-1608.24632536	11.8
**3b**	-1606.84149238	-1608.65171975	0.40091057	-1608.25080918	0.0
**3c**	-1606.83074193	-1608.64119937	0.40210651	-1608.23909286	30.8
**3d**	-1606.82974670	-1608.63834841	0.40250042	-1608.23584799	39.3
**3e**	-1606.82821551	-1608.62986878	0.40312514	-1608.22674364	63.2
**4**	-1606.86659088	-1608.66624170	0.40646439	-1608.25977731	-23.5
**5**	-1606.85731194	-1608.65560262	0.41095209	-1608.24465053	16.2
**TS 3a → 3b**	-1606.83045322	-1608.64105498	0.40213786	-1608.23891712	31.2
**TS 3b → 3c**	-1606.82037319	-1608.63474707	0.39977030	-1608.23497677	41.6
**TS 3c → 3d**	-1606.82438881	-1608.63409986	0.40260332	-1608.23149654	50.7
**TS 3d → 3e**	-1606.82157131	-1608.63333413	0.40176079	-1608.23157334	50.5
**TS 3e → 4**	-1606.81864192	-1608.61951384	0.40350008	-1608.21601376	91.4
**TS 4 → 5**	-1606.83423745	-1608.63331562	0.40701318	-1608.22630244	64.3
**peshawaraquinone – H^+** [a]	-1607.43188850	-1609.19116650	0.43565273	-1608.75551378	-1333.0
**3a’**	-1606.84791975	-1608.65337949	0.40364242	-1608.24973707	0.0
**3b’**	-1606.84062260	-1608.65004339	0.40115170	-1608.24889169	2.2
**3c’**	-1606.83027151	-1608.64131858	0.40085619	-1608.24046239	24.4
**3d’**	-1606.83098796	-1608.64057401	0.40204692	-1608.23852709	29.4
**3e’**	-1606.82612181	-1608.62729640	0.40445549	-1608.22284091	70.6
**4’**	-1606.85835952	-1608.65765172	0.40646385	-1608.25118787	-3.8
**5’**	-1606.85529132	-1608.65388118	0.41075587	-1608.24312531	17.4
**TS 3a’ → 3b’**	-1606.83478775	-1608.64760618	0.40188586	-1608.24572032	10.5
**TS 3b’ → 3c’**	-1606.81874769	-1608.63366152	0.39966471	-1608.23399681	41.3
**TS 3c’ → 3d’**	-1606.82536435	-1608.63481636	0.40162644	-1608.23318992	43.4
**TS 3d’ → 3e’**	-1606.81961875	-1608.62834211	0.40161077	-1608.22673134	60.4
**TS 3e’ → 4’**	-1606.81246810	-1608.61533077	0.40379789	-1608.21153288	100.3
**TS 4’ → 5’**	-1606.82752748	-1608.62540539	0.40615715	-1608.21924824	80.0
**11’-*epi*-peshawaraquinone – H^+** [a]	-1607.43166219	-1609.18919739	0.43549783	-1608.75369956	-1331.1

[a] To enable a direct comparison of the free energies of peshawaraquinone and 11’-*epi*-peshawaraquinone with those of the structures in the table from which they are formed, the energy or free energy of a H^+ ion has been subtracted from each table entry for these compounds. The contribution of H^+ to the electronic energy or solvation free energy is zero, while its contribution to the thermal Gibbs free energy correction and total Gibbs free energy is its translational free energy, which for an ideal gas or solution is -0.009998 a.u. = -26.25 kJ/mol at 1 atm or -18.32 kJ/mol at 1 mol/L. Note that the specified pressure or concentration does not affect the relative free energies of any of the other entries in the table, which only concern single species.

Reaction free-energy profiles

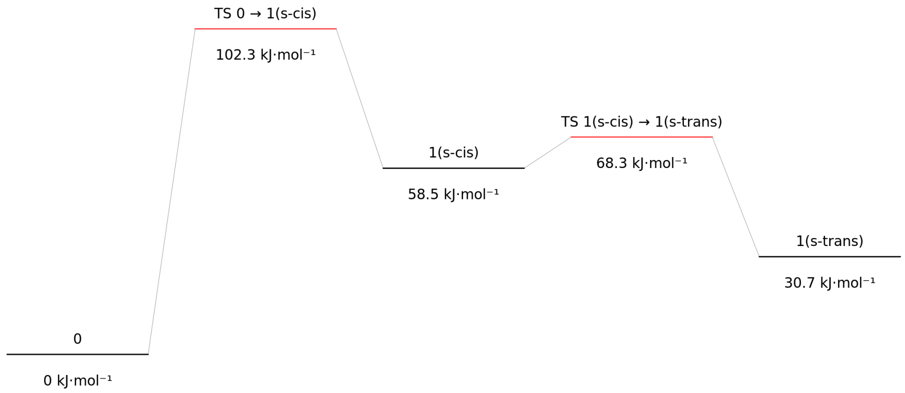

Figure S2. Calculated reaction free-energy profile for conversion of **dehydro-α-lapachone (0)** to **1** (s-*trans*) in toluene at 298.15 K.

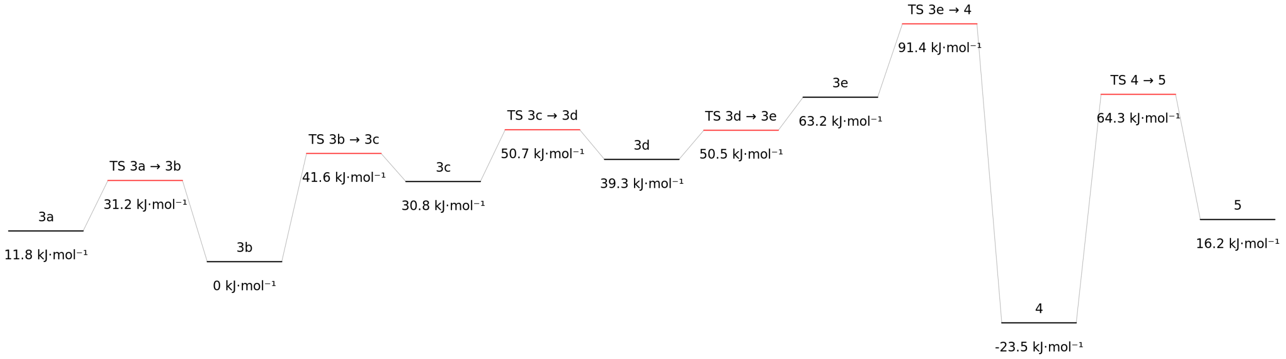

Figure S3. Calculated reaction free-energy profile for conversion of **3a** to **5** (peshawaraquinone anion) in toluene at 298.15 K. In the simplified reaction free-energy profile in the main paper, only the lowest free-energy conformer of **3** (**3b)** and the highest free-energy transition state between **3** and **4** (TS 3e → 4**)** are shown. (TS 3d → 3e has a slightly lower free energy than **3e**, which is unphysical, but it should be noted that it has a higher energy at the level of theory used for geometry optimization.)

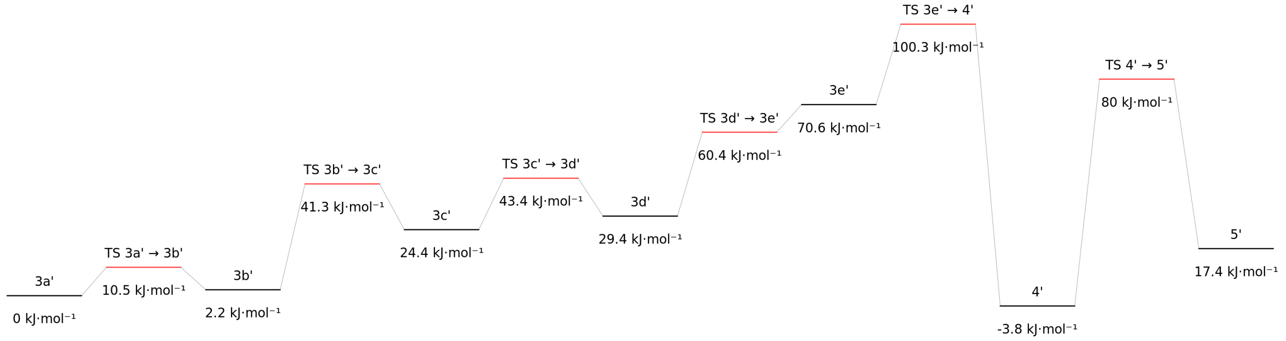

Figure S4. Calculated reaction free-energy profile for conversion of **3a’** to **5’** (11’-*epi*-peshawaraquinone anion) in toluene at 298.15 K. (TS 3d’ → 3e’ has a slightly lower free energy than 3e’, which is unphysical, but it should be noted that it has a higher energy at the level of theory used for geometry optimization.)

Molecular structures
(Cartesian coordinates in Å)

Peshawaraquinone

O	0.58649678306216	1.93823144981205	0.77722916492579
O	0.56143409198221	-1.99541524164155	-1.32252426149554
H	0.38838677383815	-1.50148872095669	-2.13973032280439
O	-1.02927970569198	0.08078294090605	-2.04166949187810
O	2.77730717450389	-2.21820258524705	0.68490818015323
O	2.81464261464536	3.06007158843199	-0.13024102641964
O	-4.07578708575867	-0.37705717671315	1.06464677751281
C	1.59833801066526	1.12728192853910	0.47061372477111
C	-1.12668220659798	-0.33504340553898	-0.91752903563728
C	-0.35624080062921	-1.53477120801204	-0.39709765769719
C	1.54628832311856	-0.21883612571801	0.57479450889253
C	-1.40461979739331	-2.58032782314606	-0.02729576288067
C	3.95937353400128	-0.37633930145976	-0.22167902716352
C	2.81327911291515	1.85670156796133	-0.03646199822625
C	-0.82766407872739	0.04414841055973	1.41416714215836
H	-1.31548773969273	-0.36360470380572	2.31130107833529
C	3.98163070930582	1.01127845693721	-0.42181324038589
C	0.26897299879731	-0.92592102279722	0.90992589105399
H	0.48137681545988	-1.73639901539166	1.61987262036717
C	-0.37825665293923	1.47462128066159	1.74739029364426
C	-2.66558461181690	-2.18810375260085	0.45402740195710
C	-1.88289802586870	0.23141652566384	0.26174425506295
C	-1.63432077855749	2.29858233419454	1.48682729428323
H	-1.41168099691835	3.37450524668433	1.44263759195129
H	-2.35268311647919	2.12587955944928	2.30531417369290
C	2.75659603262183	-1.04783351961121	0.36784235369946
C	5.11081544060079	1.62093106339827	-0.97046507024739
H	5.10240079419994	2.70411482299811	-1.10978339919864
C	-3.01893151131725	-0.73879228220328	0.61073071645866
C	-1.10763196281736	-3.93681206333344	-0.16043973642674
H	-0.12862361193375	-4.22484203248109	-0.54791605894241
C	5.07116106151386	-1.14468100037868	-0.56504390633024
H	5.02982892594737	-2.22207943163384	-0.39187813076904
C	0.22932376591739	1.63212428461823	3.13284532683817
H	-0.50091908934941	1.35632834699592	3.90662300562817
H	0.53301384723953	2.67644218801255	3.28996354284405
H	1.11479446619199	0.99004808594395	3.25269223960775
C	-3.61096869852771	-3.15714432550525	0.80881191972220
H	-4.58108383942013	-2.81787946303132	1.17905585964884
C	6.21350595087713	0.84757901393876	-1.32256053345819
H	7.09647023412646	1.32252777695064	-1.75665182491797
C	6.19309118469575	-0.53395747136199	-1.11977330086072
H	7.06041166809119	-1.13850628255742	-1.39604919575734
C	-2.18545753713208	1.74919844291477	0.16283915623302
H	-1.52089540982210	2.12139262363358	-0.62869807785923
C	-2.05455651337586	-4.89240345178301	0.19746800902159
H	-1.81469325328812	-5.95379423502227	0.09526551768376
C	-3.30752426742579	-4.50611721581155	0.68222953182820
H	-4.04602127824376	-5.26246690455633	0.95755652754429
C	-3.58858376210562	2.18181210390645	-0.15548240020740
H	-4.32110898852831	2.06129630759277	0.64768400397389
C	-4.00056333106173	2.71977284355291	-1.31312773125066
C	-5.42542591727599	3.17979920684131	-1.47589088080944
H	-6.00649290303593	3.05336749775525	-0.55189689063683
H	-5.92945128592361	2.61933747311026	-2.28081116735969
H	-5.46046527141790	4.24428754883115	-1.76323892815581
C	-3.11899408053817	2.93316491925987	-2.51855028305291
H	-2.23068936836539	2.28904109707784	-2.52127226940632
H	-2.78876310026453	3.98427859757874	-2.57831033894518
H	-3.67999414667659	2.72619042818703	-3.44317922151346

11'-*epi*-Peshawaraquinone

O	10.49480687800890	3.56161038344526	13.51369990424160
O	7.12105487417937	4.85343558744013	10.86660088646710
H	7.47926754130961	4.35990606725213	10.11084776520610
O	9.56604016900300	4.41894780278346	9.81005014698374
O	5.88370734025429	4.45569162306350	13.54902873135580
O	9.94168235505667	0.99139656038952	13.89123024215940
O	11.56910026026720	7.94668480375559	10.86645150481950
C	9.21040862410961	3.20647100182076	13.52657283534510
C	9.50070896970087	5.15868289094153	10.75322034448270
C	8.17072208698150	5.61268280551260	11.34806973194060
C	8.18944820113859	4.06480607555207	13.31248537517930
C	8.04823824839191	7.09311242609161	10.98696928915300
C	6.52328615112204	2.18037834767736	13.70437072084350
C	9.00009793679976	1.73641008597731	13.76508970884110
C	9.92702115702910	5.88121278825364	13.00131501162060
H	10.01534827893060	6.88436563241196	13.44452931753980
C	7.58345022207753	1.26992713566485	13.81900271113100
C	8.43946608558292	5.47734858057121	12.87935376261820
H	7.76821660782242	6.14910517555699	13.43114402083970
C	10.81401657377040	4.93615981955877	13.82015945500680
C	9.19239697097979	7.89975781281939	10.86557101826630
C	10.57131549918430	5.86159030967097	11.56836653902050
C	12.19864477491000	5.17579679817798	13.23153561481390
H	12.90344539448140	4.38462254322078	13.52578766775330
H	12.57754461819170	6.13113587605668	13.62725564210610
C	6.78382663247438	3.64361267087786	13.51337320030660
C	7.32211370435204	-0.08887496104683	14.00127923393260
H	8.16780746727681	-0.77406782091076	14.09198798804020
C	10.55704132188400	7.31826623134976	11.05617558655170
C	6.78710501678583	7.64922980583845	10.77185491297820
H	5.90974653052466	7.00446827243319	10.85236728960760
C	5.20728700226706	1.72563964811495	13.77778601481520
H	4.40134120914291	2.45755752869424	13.69156854963970
C	10.70935544713940	5.12365316096893	15.32618464024690
H	11.37977482955280	4.41840872976993	15.83717244231530
H	9.68253067897277	4.94346631140788	15.67844738116380
H	10.99261276587610	6.14752641053848	15.60851710499660
C	9.06447742977812	9.25502216935569	10.53924774760380
H	9.97458879871201	9.85133953310886	10.44195262226180
C	6.00617931556300	-0.53874147295906	14.06518681097270
H	5.80047492983044	-1.60266852258511	14.20524351384140
C	4.95019825254974	0.36805961721368	13.95321873762380
H	3.91825010010081	0.01221222194825	14.00426191217890
C	12.00198473889360	5.26971788949798	11.70478762778600
H	12.69005223769210	6.02395406261267	11.30089459811980
C	6.66928216968591	9.00018916279458	10.45765903887490
H	5.67788704878635	9.43155996378736	10.29719359490440
C	7.80569221550952	9.80631331485208	10.34088986090570
H	7.70394546660130	10.86430969423910	10.08862698494890
C	12.24935839511290	3.95177934544190	11.00966674594790
H	12.09211291864770	3.05432098564862	11.61796702841250
C	12.61493656568800	3.79856676237698	9.73101986617537
C	12.81309634811050	2.42767924694399	9.14456451073175
H	12.70095040536540	1.63893518181601	9.90150469772833
H	13.81179829285840	2.33091900419536	8.68713530405167
H	12.07582072951050	2.24283745729139	8.34514050970014
C	12.76665849771700	4.93950922923684	8.76301651133839
H	11.94496405222460	4.90572886555639	8.02776701987218
H	13.70914954885420	4.85969942571659	8.19763863324232
H	12.73633311667520	5.92675194020743	9.24371383044734

Dehydro-α-lapachone

O	0.35815623201590	0.71762931119774	3.27403086772251
H	0.04382453981179	-5.24662633965718	1.65342318301307
H	-1.64157452472528	3.36947596173983	3.55068146274717
C	0.92799161335265	-4.69899846016899	1.98647702610272
O	-1.54082693897820	-3.35132615233981	1.85978631190916
O	2.71508459899326	-0.50765135662536	3.47656115508266
H	4.25592624056659	-5.06887540330835	2.61620921100334
C	0.41589415548322	-0.56000204752746	2.93413562109001
H	-2.85599526622320	-1.21829454024598	2.40243829091682
H	0.57940268021161	2.66317380337941	1.50826760634484
C	-0.66529502513697	-1.27329530721181	2.52321559756715
C	-0.86850434651461	2.64761292917290	3.85159780508769
C	0.78627514618773	-3.34974796059761	2.30797561563465
C	1.77958859132715	-1.16882059321040	3.09439561444035
C	-2.01334652360315	0.71816246339847	2.71845850422060
H	-2.96515246335961	1.25443037261445	2.76586874575051
C	1.89997435257600	-2.61608030780805	2.74344859757495
C	-1.96217180998079	-0.60421489872419	2.52659543565239
H	0.08893280977777	3.17783470949806	3.95322752690114
C	-0.74604612868945	1.54230521669039	2.81277368880353
H	-1.14062919033406	2.22507716206492	4.82898898761145
C	2.17263082201276	-5.31426530981760	2.09778016339052
C	-0.35776090707651	2.09080567998868	1.44012772193002
H	-1.15036756474560	2.74944731905964	1.05526345679413
H	-0.21999424746870	1.26846118361011	0.72231631755286
C	-0.56581612866117	-2.70923175694573	2.19408748659790
C	3.14527133607532	-3.23617434182763	2.85337834853393
H	3.99488438799763	-2.64034862940755	3.19389220652522
H	2.28130411131293	-6.37201075196559	1.84596279297517
C	3.28075294779500	-4.58366783502527	2.53041196052344

1 (s-*trans*)

O	0.85246837109672	0.70276735859673	1.84411937277191
H	0.55184830660209	-5.16001854245833	3.71827156426296
H	-4.55340206399065	0.70411887806105	4.60119190150015
C	1.28000610833696	-4.72529030593736	3.03083176831675
O	-0.72696528786352	-3.15014820798322	4.10814056389415
O	2.78541757957319	-0.80020820647862	0.74573730636940
H	4.00929214986052	-5.49519332951202	1.14341446758933
C	0.88391041883614	-0.47034513638111	2.13654099148258
H	-1.73032105584997	-1.14364205316800	4.27269029467754
H	-2.92298964612109	3.38729226150439	4.97802858497077
C	-0.07649388175625	-1.13586407727607	3.04388998602025
C	-3.54239871085036	0.66636218772656	5.03842002507283
C	1.16679140011746	-3.36523480525288	2.72758514623455
C	2.01020896145620	-1.32643787796550	1.50269268436089
C	-1.50407134468845	0.89502314072492	3.54065419774253
H	-0.88038899860281	1.54059515030848	2.92165858600783
C	2.08737585643510	-2.77315654253050	1.85036821987694
C	-1.12154644776027	-0.48854359736339	3.64070015531915
H	-3.61133082191559	1.14720867239358	6.02722477865087
C	-2.58580802139411	1.42547536142313	4.16612534170276
H	-3.27638161713633	-0.38601612715810	5.18735748287409
C	2.29694293978607	-5.48546211651100	2.46542650311263
C	-2.90488186139343	2.88215754751382	3.99830901377486
H	-3.90988580562588	3.00730490533200	3.56244108228843
H	-2.17783415892952	3.39252841992434	3.35371009996087
C	0.04990754104065	-2.59018028129070	3.35962022725585
C	3.10758178185918	-3.54395322092703	1.28282105053134
H	3.80745804369964	-3.05379840209223	0.60273432734037
H	2.38045340298718	-6.54778907394770	2.70730450350549
C	3.21173686219115	-4.89535197927493	1.58828977253134

1 (s-*cis*)

O	0.41267003826450	0.73938731349779	2.81418551716372
H	0.02252525442060	-5.39168835482907	2.32422836693630
H	-1.77272159749787	1.45824422596802	5.93796899197831
C	0.93260956343559	-4.79003190040456	2.36369772906493
O	-1.54605917778712	-3.55720391913668	2.42985305527640
O	2.82665707508583	-0.40631787121919	2.65928177873923
H	4.32998474028444	-5.02798576027233	2.31816785254942
C	0.45295837458055	-0.46596193218833	2.74312232249606
H	-2.74946028752644	-1.67156229572120	2.43192773624379
H	-2.42826233948204	3.45202102364083	3.96748533653427
C	-0.73754905583010	-1.34571869627883	2.70951850414229
C	-1.36365006396019	1.09982119099306	4.98101500521431
C	0.80044585550209	-3.40226249871427	2.47112045108422
C	1.85503374529656	-1.11807070623617	2.63534333854625
C	-2.59939792578958	0.42457378823580	2.88894287098146
H	-3.44427075830304	0.64394103563960	2.22507997734421
C	1.94957311752733	-2.59990757903520	2.52403400910186
C	-2.02357093526491	-0.88706296414320	2.68566526402632
H	-0.44842982446286	1.67462575847867	4.76507654558885
C	-2.35004499213381	1.31320860777391	3.87824403460204
H	-1.07265781586580	0.04659234829651	5.09014749036463
C	2.19473847922313	-5.36993311770648	2.31107277991287
C	-3.12464963058190	2.59808219095742	3.94413264741094
H	-3.72011140724763	2.64960247767162	4.87025791219954
H	-3.79888159991402	2.72409106322879	3.08649582142442
C	-0.58207685755241	-2.82530924224049	2.52430648843940
C	3.21720241915093	-3.18901577396269	2.46782218381052
H	4.09296856250776	-2.53781590572068	2.50745779245702
H	2.29164507098695	-6.45521999331021	2.22842191651632
C	3.33948197293346	-4.56892251326240	2.36222627985003

TS dehydro-α-lapachone → 1 (s-*cis*)

O	0.48051408190421	1.82249869529119	-0.13009646476337
H	-0.47031644424604	-4.22643703199820	0.37576645673841
H	-2.19518674944206	3.23901569804039	1.82599614941158
C	0.48894024437834	-3.71288137713672	0.28373440004402
O	-1.87750557525556	-2.26704698871975	-0.09010270382928
O	2.71073963979676	0.41903184020207	-0.64142486977256
H	3.85471094956801	-4.24387858248577	0.31326372924004
C	0.38690496032452	0.58828708501133	-0.16612925191828
H	-2.74139113529012	-0.21976196101180	-1.10537586446763
H	-0.10609169815128	4.39129054020293	-0.44983859056681
C	-0.83536225319068	-0.15882853012585	-0.17120708485004
C	-1.33985970163281	2.66914521016948	1.42406727990717
C	0.47825097034233	-2.33585072336089	0.05571946135396
C	1.70857024401064	-0.17937436600488	-0.34252242836805
C	-2.14457849519243	1.82746613982930	-0.82078086461246
H	-2.75500646136976	2.19399120911450	-1.65289792230343
C	1.69031575500955	-1.64604408756203	-0.08239283162207
C	-2.00457826894606	0.45793070480822	-0.65963570353561
H	-0.41532064256394	3.11879736041170	1.81028650639871
C	-1.40937843815487	2.76377353448822	-0.07119449928848
H	-1.42705728220321	1.63555500902885	1.78033890314834
C	1.69863722835645	-4.39371476001439	0.38035408617004
C	-1.15656824892413	4.12603513067111	-0.63815246573355
H	-1.78042970041739	4.87668777789187	-0.12681943474156
H	-1.34365637996689	4.16930537103260	-1.71838767905341
C	-0.84055473248631	-1.63015921227404	-0.07213978937715
C	2.90331216355851	-2.33492172655184	0.00532678094443
H	3.83172675462106	-1.77307089949188	-0.11785792996955
H	1.70349862039044	-5.47148380482688	0.56098190284550
C	2.90672159517274	-3.70535625462881	0.24112172257110

TS (s-*cis*) → 1 (s-*trans*)

O	0.49695410400792	1.94288429922197	-1.00080364448523
H	0.24941028155109	-4.18454217772457	-0.49490137551457
H	-2.38247859995247	2.22504074308799	2.97348192719443
C	1.14530143688590	-3.56705912000059	-0.58396599898263
O	-1.35396443651009	-2.36649671781593	-0.41552272221243
O	2.96736730255201	0.85513513178480	-0.84219000270345
H	4.53708683024226	-3.75273136033233	-0.82788751528614
C	0.59227864757052	0.76171046244207	-0.78734587913307
H	-2.57830453191688	-0.43451433668127	-0.15648355351245
H	-1.89933470162262	4.65455919486010	1.02578173272323
C	-0.57113297512300	-0.13299420533988	-0.52983172714882
C	-1.64608974397712	2.08725353604748	2.16516537328153
C	0.98703834265322	-2.17825218863935	-0.62064425588971
C	2.00629961682853	0.13152101940919	-0.78638164562779
C	-2.25327709013344	1.74885051319695	-0.23611037728993
H	-2.67474176215052	2.14071386291034	-1.17064687732855
C	2.11888077167356	-1.35442418590467	-0.73326657183204
C	-1.81390058722841	0.34355610632114	-0.29819534863597
H	-0.76614703099560	2.69765528125351	2.42649247585338
C	-2.21500661919430	2.53487193578473	0.84649196111026
H	-1.33553357210190	1.03334268943213	2.15859155930282
C	2.41434371548468	-4.12886876463889	-0.65895865408323
C	-2.71680197821443	3.95091469271130	0.79829589112789
H	-3.50212472930760	4.11743971436874	1.55389018764532
H	-3.12444998445600	4.21037286355687	-0.18813787055754
C	-0.40132514847775	-1.62388492031192	-0.51864352195760
C	3.39325585640226	-1.92724310803677	-0.80622943011153
H	4.25470099559578	-1.26163712349301	-0.89259006240016
H	2.53097152133834	-5.21489445928595	-0.62987951868050
C	3.54072406857606	-3.30827937818423	-0.76957455486550

3a

O	1.46143745230158	0.97605254176209	-1.94704748373984
O	-1.35562768685257	-2.03380519067658	-3.31088598715364
H	-6.93303165921312	-0.30920726762597	-0.60342486269877
O	-3.17949922121419	-0.05036787901232	-2.93377042926610
O	2.70620016319293	1.76397419586297	2.55431993714910
O	3.45857165373763	-0.81682280561245	-2.11871471800445
O	-1.78916258476456	0.23175833920694	1.56133952904571
C	2.09986822393275	0.75459461941893	-0.94223659375355
C	-2.46171689932844	-0.37079996569797	-1.98308817653594
C	-1.42008797805945	-1.48270121279966	-2.23657559925734
C	1.88314161349643	1.40753031345837	0.35860567378994
C	-0.49207499154158	-1.84948055425088	-1.12044382369047
C	3.82629809148331	0.07446113291373	1.36366498353211
C	3.23543582398891	-0.29262266277977	-1.05656484378121
C	-0.33962017401581	2.46206743445691	-0.20628124166701
H	-0.29741733820460	2.14063537331605	-1.24631683799985
C	4.03089428181605	-0.61623836106034	0.16403395545428
C	0.78128093052552	2.18875996251213	0.62790933419774
H	-5.25921434258448	-0.39258345843629	-1.22508909563209
C	-1.53782292649190	2.87039803082067	0.30786823380231
C	-0.65683421379459	-1.27257286080910	0.14335540995488
C	-2.52479006612096	0.19721358711153	-0.67731378299010
C	-2.74789939037910	2.74667935881404	-0.56808689022905
H	-2.43601184858350	2.86544523369304	-1.61637811487521
H	-3.47967407087555	3.53929352595570	-0.33562862084902
C	2.78800246008807	1.15615800467400	1.50055147018409
C	4.99117075105575	-1.63028277431521	0.09271773999780
H	5.12028306228867	-2.14670895050181	-0.86099466978589
C	-1.72145552993195	-0.23162682717125	0.40981118037278
C	0.54267405776701	-2.75906898217346	-1.35068972964942
H	0.64621454604560	-3.17655935544345	-2.35456129174250
C	4.58980510021521	-0.25763781718192	2.48691729337194
H	4.40814750814209	0.29953604306450	3.40817196778869
C	-1.74190074571535	3.30104916183877	1.72940364751011
H	-2.55454824441692	4.03852997071107	1.80125459034844
H	-0.83885776547241	3.72727573842532	2.18630461778123
H	-2.02885068210827	2.40387191568826	2.30203863051890
C	0.22237823683482	-1.61664984602212	1.17245909784213
H	0.07215322300091	-1.13591482292446	2.14202137123118
C	5.73890372588962	-1.96209687640737	1.21531849347648
H	6.48299433293252	-2.76098290224151	1.16044185894575
C	5.53730687798792	-1.27205328985540	2.41516994709866
H	6.12552148308559	-1.53174087140579	3.29939021116588
C	-3.47438646779296	1.35699751426955	-0.48686223343648
H	-4.10419360769957	1.34878349775014	-1.38942402526553
C	1.41896660716420	-3.09447994605579	-0.32295114281572
H	2.24024768814750	-3.79253807502958	-0.50770687256027
C	1.25662801753952	-2.52042745950941	0.94146344822885
H	1.95514622924983	-2.76894217658758	1.74634213642817
C	-4.38374694775762	1.23493692347201	0.71390498269657
H	-4.13942720737069	1.83424338134972	1.59746465084425
C	-5.45486638582210	0.43339726399403	0.78890099436771
C	-6.29202916798357	0.36571782288944	2.04001173163487
H	-7.35420823234816	0.58090421102824	1.82591813195262
H	-5.94355524706625	1.07977917987861	2.80093283800743
H	-6.25858951644465	-0.64441884306283	2.48492603433757
C	-5.87984330256070	-0.48968602576206	-0.32384122515524
H	-5.81744304708473	-1.54091012297465	0.00697480307630
H	0.74785728969038	2.54886948465022	1.66114342560016

3b

O	3.33377965115317	-0.27611200749862	1.25550797128321
O	-5.53381002250247	-0.53826049316330	-4.55077925620381
H	-4.58240278038100	1.14339570713886	1.83659242377088
O	-4.62609641501412	0.83121917537426	-2.39083667894951
O	2.09499396689933	3.61979875323230	3.65824005385038
O	5.51037751823590	-0.47311557099530	2.81610196064292
O	-1.14847993143180	-2.33508132830212	-1.97406650407974
C	3.47599607523395	0.56153142326772	2.11802564116576
C	-3.99949877787832	-0.17477390463053	-2.74272219726142
C	-4.55643862405474	-0.94092729305985	-3.96132177176691
C	2.55645690700238	1.68102532472835	2.37131860854580
C	-3.85810772946399	-2.19323972200362	-4.39337277112350
C	4.05240533096711	2.48716917406680	4.29574859815090
C	4.73628712902522	0.43010610607694	3.01242257835735
C	0.85506696453272	1.12706755972141	0.57005727578934
H	1.39530527217891	0.24222492745574	0.23352641860565
C	4.95259599747950	1.43311432838156	4.09558802581150
C	1.40256156736246	1.88553864603329	1.64352137544438
H	-4.68220361167715	0.74001075920353	0.08891695187089
C	-0.32393581406270	1.44889328538068	-0.04690897149027
C	-2.71782522728162	-2.63019258886788	-3.71044257060951
C	-2.81652706128808	-0.67391240268888	-2.13026591976995
C	-0.83902584219013	0.59085319679094	-1.16270982543310
H	-0.18494134262484	-0.27752167720864	-1.32179220094277
H	-0.82918284431096	1.19693294868281	-2.08726292760952
C	2.82614217815458	2.66931734775471	3.44097532036958
C	6.07901744652065	1.30701172545953	4.91604686393681
H	6.75931069454620	0.47296103895370	4.72997436739407
C	-2.15765480300748	-1.87025022989200	-2.52509536023776
C	-4.34828400193905	-2.91750099335500	-5.48310336489746
H	-5.24046831448613	-2.54023759943935	-5.98919466131170
C	4.28971954040964	3.40856167139193	5.32031734816042
H	3.57117047234677	4.21998362427211	5.45242721523723
C	-1.19307763492402	2.60870181490633	0.33378260226761
H	-1.60151811519391	3.08793358543624	-0.56827705111571
H	-0.69298647515925	3.36779614786319	0.94662417518382
H	-2.05774310066312	2.22224101797319	0.89832102303192
C	-2.07656129603575	-3.79750510021075	-4.12804286074814
H	-1.19028292890409	-4.10691460456125	-3.56911921742916
C	6.30721316273937	2.22585108530232	5.93253259470043
H	7.18686237314694	2.12578179819923	6.57355029323336
C	5.40906819028285	3.27876303245248	6.13413262430419
H	5.58701051697935	4.00227818792652	6.93405050589448
C	-2.29193930473377	0.10406991862070	-0.94305282875107
H	-2.94176434975790	0.98851131816160	-0.89279830850155
C	-3.70508070170885	-4.08092143810844	-5.89498148903197
H	-4.08865532986336	-4.64908595272856	-6.74732787996196
C	-2.56604285574248	-4.51943542252721	-5.21358573376413
H	-2.05744467263777	-5.43370892084003	-5.53412050585533
C	-2.39408736985661	-0.66120020306890	0.35527644190129
H	-1.58341800418920	-1.37734946638975	0.53812852542332
C	-3.37819763720770	-0.56636698475520	1.26159458619683
C	-3.34847161276800	-1.38820001651721	2.52516765996542
H	-3.40438819307661	-0.74319761008938	3.42011418606661
H	-2.43230175921190	-1.99216858502969	2.59599734822371
H	-4.21462496322434	-2.07122742457689	2.57576674531728
C	-4.58953466262725	0.31900847755062	1.10016520825435
H	-5.50757023388348	-0.26115117758900	1.29512643944011
H	0.84837533176721	2.77089118993757	1.97171495825383

3c

O	3.34510364651767	-0.71365624516942	1.90047278128380
O	1.37810873759707	-1.65888214990056	-7.53977813779160
H	3.34382940609708	3.36862247168679	-3.38673898020261
O	2.54055095295355	-0.25040263557054	-5.53197708477877
O	2.15313834462418	3.67963841603807	3.18088255835233
O	5.46739481332216	-0.55637429497667	3.53357627529984
O	-1.54418851700843	-0.44325867633585	-3.16956153300440
C	3.54158088525117	0.34440123548203	2.45559226033542
C	1.36298502085450	-0.59475610946758	-5.38947848094742
C	0.72391324887692	-1.36311157927064	-6.56553608564424
C	2.71979466397338	1.55258384952602	2.28829753944140
C	-0.72483637222380	-1.72979686554228	-6.47043851146055
C	4.04797236258922	2.70633871644871	4.16703650347114
C	4.72766212425139	0.39237660723268	3.45145221618013
C	1.17485018566917	0.83353334061923	0.34871982858755
H	0.09182341798078	0.87463527934279	0.18699589769379
C	4.91925615380781	1.61646767486729	4.28069688035355
C	1.63349403435458	1.63106892061109	1.44003852295850
H	2.83784355462722	2.03811576090825	-4.47933033899524
C	1.89317264114231	0.22458110314609	-0.64100152502689
C	-1.45269799675658	-1.41217942874889	-5.31848939763203
C	0.56078498970265	-0.36016689724878	-4.23652989241283
C	1.16122180622474	-0.43600979036561	-1.77094152200598
H	0.10551145480696	-0.59802484341173	-1.51275107077253
H	1.62462566553199	-1.41889937131969	-1.96209470562180
C	2.89514396143419	2.71494999807064	3.19909685955957
C	5.98276306025604	1.65888047459805	5.18887384520469
H	6.64106546617791	0.78947471074526	5.25046975590601
C	-0.81763838801407	-0.69216712454953	-4.14563396988601
C	-1.34053977610489	-2.39575369137685	-7.53320704432845
H	-0.73418459994128	-2.62705991738315	-8.41258227275790
C	4.24885226268748	3.83385865173776	4.96930573237210
H	3.55408271554264	4.66890056042320	4.85928347364596
C	3.37863705144102	0.25868589074703	-0.74603451075069
H	3.78319860288863	-0.67265938770014	-0.31508568828408
H	3.82671201074546	1.09498511036869	-0.19114520404816
H	3.68583347106906	0.31294079350040	-1.80005601284565
C	-2.80117720532418	-1.76539861157557	-5.24459692011090
H	-3.33671872512780	-1.49952091174078	-4.33026180057880
C	6.17647027655849	2.78288681735361	5.98171332213676
H	7.00725763622289	2.81557998778576	6.69120965659581
C	5.30619162544355	3.87214743236571	5.87077659240087
H	5.45792431532301	4.75730412901534	6.49402041770683
C	1.22579309406347	0.37467716810387	-3.09264302166261
H	2.28119452404125	0.45211227078238	-3.38739843234324
C	-2.68539463565513	-2.74496491870752	-7.45398255452323
H	-3.16859366895897	-3.26506547559628	-8.28612577118440
C	-3.41507843464967	-2.42741468402578	-6.30483001352415
H	-4.47272833018992	-2.70011787224532	-6.23830881476853
C	0.66551011302005	1.76184174914985	-2.89965908076282
H	-0.31418079342300	1.79988742904463	-2.40690233722242
C	1.22521926030281	2.91333624836825	-3.29683052850871
C	0.55184840357652	4.23445330056062	-3.02462815175188
H	1.21433961453565	4.90640269701629	-2.45036239339726
H	-0.38005411919688	4.10596740006173	-2.45479797608351
H	0.30907537401188	4.76038107790699	-3.96484918834607
C	2.53454986563668	3.00024210992046	-4.04293261518775
H	2.45071791268566	3.72305402102471	-4.87201066476759
H	0.98508283415370	2.48577807766883	1.67474131443574

3d

O	-3.52253119332530	3.32819677824893	3.32638380705944
O	2.25073674995084	0.30798437759070	-2.97268200617399
H	-1.17508135210913	2.27607647238182	3.19042686561225
O	0.64851576150187	0.42562063525997	-0.78614934601237
O	-6.57695129253304	-0.21219808064329	3.97448072918512
O	-4.75617156624350	4.68803743611167	5.28583570291125
O	-1.46144595268892	4.21550204976068	-2.66122282899717
C	-4.51586852327977	2.83219923203158	3.81031536880081
C	0.49255268122273	1.28132710670903	-1.65980852080063
C	1.42290646790510	1.18692332145473	-2.88919495021006
C	-5.12389799083251	1.56881639986756	3.37303752859069
C	1.27288079814382	2.20786475344485	-3.97436272597873
C	-6.69253975794529	1.63972109280083	5.41247606438193
C	-5.14981291979985	3.58323470962877	5.00625056279254
C	-3.85881028193730	1.09647170766708	1.16248235806829
H	-3.37616206462329	0.20693919846121	0.74152537631298
C	-6.23185461048503	2.90976307339049	5.77961458197939
C	-4.67829350296894	0.82379911630313	2.29622028130232
H	-0.09554999170432	1.67938081318145	1.89405878882178
C	-3.74802982895134	2.23764809191796	0.41293286390003
C	0.30027306168829	3.20769005034633	-3.86396160304921
C	-0.46854353370190	2.33528696365660	-1.63250773460636
C	-2.85186397960641	2.22110307802803	-0.79300041071039
H	-3.12242778730242	3.02146573234598	-1.49603475515646
H	-2.93980258164417	1.25557238681082	-1.31499338197708
C	-6.14718370470628	0.90072816560400	4.21915622409726
C	-6.77972693123688	3.56302644186191	6.88878115400046
H	-6.39401547010688	4.55243278352735	7.14467362835931
C	-0.62092960376057	3.29903358953068	-2.66327950805581
C	2.10587736917831	2.15337184962379	-5.09487350609349
H	2.85092712940738	1.35507850598034	-5.14044815448503
C	-7.70243586286984	1.03143566128695	6.16423274254401
H	-8.04367198360848	0.04170760257809	5.85439818864802
C	-4.48586093489690	3.49769295408296	0.69616565613666
H	-3.82063543912859	4.15240520910677	1.28658342940588
H	-5.39997323423160	3.33783236836909	1.28651452802761
H	-4.72983599896242	4.01866881689372	-0.23984014795538
C	0.17309365257885	4.15035154141011	-4.88596274013406
H	-0.59680451735963	4.91584527822757	-4.76442970511466
C	-7.78220214425460	2.95170001726649	7.63112438385612
H	-8.20899379495109	3.46138767065286	8.49873953570638
C	-8.24342021033677	1.68260376169707	7.26673034419373
H	-9.03252191535110	1.20075182101652	7.84993072514676
C	-1.35330180454544	2.40457371496702	-0.40917910347766
H	-1.07347571658212	1.53853648301188	0.20498573361008
C	1.97481258610594	3.09641184616827	-6.10987318054671
H	2.62746292104387	3.05541742066110	-6.98674172109036
C	1.00435912582175	4.09666337895266	-6.00185539923890
H	0.89791742622582	4.84063509015591	-6.79729963998242
C	-1.16305346333586	3.66591558667675	0.39461988243573
H	-1.41418102248019	4.59106061363791	-0.13928001924802
C	-0.75803222995119	3.74440087283291	1.67052665150684
C	-0.72512862095767	5.06655246773546	2.39248091389846
H	-1.43478441722846	5.04881521905761	3.23769727692656
H	-1.00385043670497	5.89781866352918	1.72793174683763
H	0.27580489595871	5.27708283766456	2.80828258490796
C	-0.35693300691740	2.55382091165406	2.50490239439617
H	0.51967702012964	2.80013482134410	3.12707639192969
H	-5.04654256071384	-0.20996012352416	2.33803648280427

3e

O	-5.10278453710897	4.06993288875553	2.80253880133712
O	-2.21055416420408	0.27002262512642	1.49424342025115
H	-0.31366198243024	6.51260746054247	3.06324885418494
O	-2.43792442380274	2.88037590908404	2.14681802703033
O	-6.45592420797668	-0.14433465915321	1.40865978696642
O	-3.77117143147402	2.93616905499302	4.88397051777091
O	-1.05759623998127	3.93773173545716	-2.23750360604324
C	-4.91547977355223	2.87870684193480	2.79212508167483
C	-2.01569937229093	2.60372457922437	1.01101796954154
C	-1.81065356655135	1.10919267576884	0.71734331529853
C	-5.42036288261133	1.97167973040990	1.72856143913764
C	-1.12347833588884	0.72473998843316	-0.55185359770673
C	-4.93147570047106	-0.04093264481338	3.21106966324248
C	-4.19899689079616	2.24043435542986	3.99926339352844
C	-4.80380990948310	3.32500542898502	-0.34069397155476
H	-4.15390376413610	2.88609304801597	-1.10835009867858
C	-4.16665589708789	0.74863923050887	4.07778139049740
C	-5.46272673367263	2.26307000089304	0.39749202744031
H	-0.95816775305478	4.95649899833651	2.46557554261259
C	-4.68043536992794	4.66011035595389	-0.17202874422180
C	-0.84374202536155	1.70609476592929	-1.50851088554670
C	-1.78902907647153	3.52875715447579	-0.03394003842987
C	-3.45365776805222	5.32802544072700	-0.73598797926816
H	-3.58447690793221	6.42269777964064	-0.74752720982570
H	-3.23103285142759	5.00863593036477	-1.76621022327284
C	-5.70750849672345	0.54961500076124	2.07001779386847
C	-3.41755763885778	0.14460093739134	5.08983117791269
H	-2.84015576304255	0.79201754288153	5.75314345019491
C	-1.22934949506833	3.15628946330688	-1.29389077615755
C	-0.78977824932193	-0.61090462393671	-0.78762785643118
H	-1.04117600214656	-1.34705736074083	-0.01998956096025
C	-4.94275449136826	-1.42986483338112	3.36767411644511
H	-5.54818338754889	-2.01808776450355	2.67494662512153
C	-5.66353775724093	5.54107085891238	0.53805218335997
H	-5.17300839265776	6.13815436985534	1.31973377498555
H	-6.47913482070341	4.97589968130925	1.00128337360142
H	-6.08207101447355	6.24377445552558	-0.20502802285109
C	-0.22069235090125	1.33730885887931	-2.70151473001478
H	-0.02821778492572	2.12642211573385	-3.43203301563772
C	-3.41600191369686	-1.23757749614723	5.22809574308984
H	-2.81451106420607	-1.70815455846903	6.01064231923908
C	-4.18453381485953	-2.02698210218538	4.36690335530451
H	-4.18441679658782	-3.11499564716667	4.47564102345188
C	-2.22763835966837	4.96604865437750	0.16424515939158
H	-2.57355749399898	5.02634424260907	1.20741898449885
C	-0.15827481467103	-0.97065199466723	-1.97462130803656
H	0.11030108841402	-2.01452471426867	-2.15984062873500
C	0.12552176654887	0.00789521686192	-2.93153484874706
H	0.61769421605786	-0.27276755362593	-3.86754486391517
C	-1.10406310460450	5.95846198756444	-0.02362858271748
H	-1.00461758264218	6.39080651058109	-1.02620654043000
C	-0.20882785437509	6.30463326492400	0.91183116423700
C	0.88482358206215	7.29803499877496	0.61276304251339
H	0.85022780442156	8.15299836344564	1.31148196361053
H	0.80955541217344	7.68972938121154	-0.41239596913343
H	1.88240095977679	6.83862021877150	0.72921847391736
C	-0.18708160181254	5.72230461712149	2.30226432080428
H	0.79151631464908	5.25086687365177	2.49923283789683
H	-5.79732562225252	1.41435269961642	-0.21303666564393

4

O	-3.59688082418966	3.03336513682893	1.86250239806054
O	-3.65231401583945	-2.20003539190690	1.36139199502365
H	1.52755134410706	1.73137943842286	2.81047784450054
O	-2.22484953371219	-0.06631263614691	2.19650598738078
O	-6.82473694228032	-0.26885754352533	0.92512137311763
O	-3.44889841421361	2.09131802118063	4.34868277934172
O	-2.06300557670692	1.44010927785567	-2.27381098784877
C	-4.33194258599461	1.97771901403720	2.15482052992160
C	-2.46346774604416	-0.15818245963690	0.97978160530720
C	-3.29471059891328	-1.37908961417818	0.54580484779998
C	-5.21940216886254	1.40809594860302	1.29775820038482
C	-3.68120042975027	-1.50300407559210	-0.89287116189639
C	-5.71008503863131	-0.39679057135633	3.01159698095681
C	-4.10766360996355	1.46856058241137	3.55082659901752
C	-4.46773679685810	2.91896356835630	-0.42925066482005
H	-4.45459234015479	3.29210546164523	-1.45651414661022
C	-4.79394816461522	0.18670027202882	3.89382301619407
C	-5.31089459641490	1.95539417571156	-0.05228899148894
H	0.04705590531609	0.80765494287083	2.39662471873318
C	-3.46719580066656	3.55643734309324	0.51044349722311
C	-3.24319596863634	-0.54872544720619	-1.81655652026704
C	-2.09881470310483	0.78539966652664	-0.00897455729335
C	-2.00512309225163	3.35966372105227	0.06539884674971
H	-1.40004687296204	4.16646623745815	0.51459704640345
H	-1.96831596511648	3.48616285050448	-1.02810505119965
C	-5.99447266262573	0.21768618338589	1.67272199396801
C	-4.52982336334454	-0.41425640313885	5.12388182955512
H	-3.80751938511639	0.06564152604445	5.78738658095141
C	-2.40221643837140	0.63610612601425	-1.39320981614729
C	-4.48914642804345	-2.56547982575864	-1.30327098803516
H	-4.81930204724983	-3.28028813154388	-0.54590188219399
C	-6.35112386444122	-1.58398362315779	3.36199993312168
H	-7.04835930805316	-2.02062980496570	2.64420369350910
C	-3.77894089729607	5.04898204205801	0.63435376565055
H	-3.13786315439406	5.50026043201652	1.40611746679472
H	-4.83235644676981	5.20077288076514	0.91286150366815
H	-3.58974627695490	5.55574360831278	-0.32419785236411
C	-3.61400367279988	-0.67433572218336	-3.15601854644414
H	-3.25484889501321	0.08989391518207	-3.84903500035458
C	-5.16798188090392	-1.60153860892165	5.46750497137510
H	-4.94908882012886	-2.08105160611786	6.42547387652239
C	-6.08026592185847	-2.18640382484962	4.58610737460292
H	-6.57461006782245	-3.12418491527179	4.85390059583627
C	-1.35124381512920	2.02070750218207	0.45342432868806
H	-1.36126171266509	1.97209711381301	1.55003363684480
C	-4.85787758938817	-2.68452119129026	-2.63965256724029
H	-5.49453011693579	-3.51317662579093	-2.96236004090690
C	-4.41588673982538	-1.73641306092633	-3.56653389576755
H	-4.70556697944510	-1.82622921452766	-4.61792038518005
C	0.08534383025554	2.01143184956074	-0.01554628803134
H	0.24119029786218	2.35142929676867	-1.04748013048199
C	1.15044967553241	1.59156289448103	0.68216292095658
C	2.53316015214381	1.62998103025103	0.08133970389711
H	3.22474063248026	2.21857559335880	0.71060296219979
H	2.52847130419346	2.07077276697333	-0.92655699639384
H	2.96344521650689	0.61538752155859	0.00842707904110
C	1.07531531264765	1.03445371441971	2.08204770092296
H	1.65220681777939	0.09592647704961	2.14634006425267
H	-6.02622221836564	1.50142216521023	-0.74038377750922

5

O	0.62504080206311	2.00454578688818	1.26213024788876
O	1.04185751077522	-1.84228601117308	-0.79404516376326
H	-2.33347111146683	3.94937779274844	-2.44235032434439
O	-0.58902546587151	0.36778069285733	-1.76253184458785
O	2.93782951315743	-2.08811037702817	1.30985232886434
O	2.89476667836405	3.02293359735861	0.20989660406130
O	-3.99997454019804	-0.38039259689253	0.85199205810689
C	1.62544098614173	1.15561655534606	0.92847420141587
C	-0.76938532552650	-0.18544644355967	-0.71235659527498
C	-0.03004667583728	-1.43459635306297	-0.16762596317961
C	1.55208323613514	-0.18060937663285	1.06400888977152
C	-1.16696059239830	-2.47238849761125	-0.03006678298790
C	3.65937448784215	-0.48997026262146	-0.28757676471220
C	2.77524255535339	1.81652027481991	0.25123902185834
C	-0.86124933789130	0.08691661597018	1.68224646992232
H	-1.48272059396006	-0.31050938265553	2.49942008031927
C	3.73133603928625	0.89846905738017	-0.45333655973678
C	0.26930632604466	-0.87105156817614	1.32431328019353
H	-1.90762502330327	2.21621224957737	-2.38828240543859
C	-0.43644245034866	1.50992261479615	2.09026139460015
C	-2.49058426421470	-2.12743920500857	0.29231464348690
C	-1.73104077643979	0.29567826022209	0.39336048494250
C	-1.66490667141471	2.33454516707681	1.71194316497923
H	-1.44854319146503	3.41377088812842	1.71762528513394
H	-2.46822865347706	2.13491210336145	2.44211402343070
C	2.68500460493049	-1.07075073369056	0.69269160740206
C	4.70284950947945	1.44880505586838	-1.29441803268692
H	4.73983108568188	2.53562808990145	-1.39882045803810
C	-2.87790897354532	-0.69874255415153	0.51750545479703
C	-0.84412490265584	-3.81211497764230	-0.25670790998707
H	0.19485887242729	-4.01317125529179	-0.53252312179740
C	4.55924826173731	-1.31938530957507	-0.95378974638634
H	4.48157944423766	-2.39579952969192	-0.78831782798304
C	0.00374488106722	1.62089205417786	3.54349649883034
H	0.28133728357612	2.66024142403461	3.77174442416227
H	0.87863449364198	0.97968987445832	3.73032796863713
H	-0.80330730130248	1.31077790077656	4.22372786082926
C	-3.46907682882059	-3.12576000592743	0.40748379703185
H	-4.48810516587869	-2.82146006262646	0.65944047631797
C	5.58172804296583	0.61732483572568	-1.97989173402259
H	6.33001938517943	1.04676946306106	-2.65178577396807
C	5.50607650870877	-0.76906324414096	-1.81130551399871
H	6.19365134672827	-1.42201693294574	-2.35666238180608
C	-2.05884531799639	1.81096085269082	0.32193881159367
H	-1.32460818461501	2.21892681911746	-0.38617508380173
C	-1.81698787727316	-4.79625389261321	-0.13464263492155
H	-1.55494351889500	-5.84520897958360	-0.30512234427424
C	-3.13540032688929	-4.45651912505553	0.19926782297920
H	-3.89776791820879	-5.23525154613758	0.29146356520955
C	-3.42969671121722	2.23025458921698	-0.12887392256494
H	-4.23359647826431	2.11036449985736	0.60425902983981
C	-3.74164313510408	2.73382000400005	-1.33199415512411
C	-5.15428733259045	3.15330381508257	-1.64649071967736
H	-5.18965005069517	4.20489242396708	-1.98176995577810
H	-5.81810127049288	3.04516963649547	-0.77653091923531
H	-5.56903042276652	2.54682958660299	-2.47023098247309
C	-2.74485215720463	2.92456670774642	-2.45040786587647
H	-3.23198949162601	2.78647303408962	-3.42918842127152
H	0.40191247883004	-1.71386765330602	2.01547228219263

TS 3a → 3b

O	3.08791437830806	0.51041087989160	-1.34779632724410
O	-3.92230056579460	-2.61920135492600	-4.30353523317909
H	-5.97427968913821	2.25340833543705	0.13658615371095
O	-4.45645012030608	-0.26984974467651	-3.05322657666166
O	3.13836457456618	2.24389685190379	3.06288697503603
O	5.61231780802141	-0.33955130431335	-0.97864701787464
O	-0.79083458526176	-1.06327439329063	-0.20045747408081
C	3.58738661043930	0.70531150998361	-0.26222164012054
C	-3.47762196973736	-0.91904416555143	-2.67083614644989
C	-3.22304857726120	-2.26631306473924	-3.38087914200202
C	2.91891389748617	1.35449710350139	0.87442393099440
C	-2.09138067163301	-3.12090291945310	-2.89990023423158
C	5.07856644972415	1.19828415859173	2.24987164418983
C	5.05423296768555	0.23650957262149	-0.07826766961562
C	0.61470399755410	1.55390618435779	-0.16606098739162
H	0.88807371144527	0.98448363235639	-1.05297724909173
C	5.74055371735506	0.52555537234297	1.21555590663346
C	1.59065911113291	1.73067267775711	0.85058198968403
H	-5.52972145023877	0.99539100496011	-1.06267230134457
C	-0.66100950068810	2.03084834357066	-0.05785831122962
C	-1.28098451332101	-2.68215942092882	-1.84705478972661
C	-2.56313189861271	-0.52683420511610	-1.65215856227839
C	-1.64260428316109	1.83615206167402	-1.17732407966000
H	-1.10354048081740	1.55540112052269	-2.09276336770991
H	-2.09795133486796	2.82705659993468	-1.35773469864261
C	3.64740941050731	1.65178397417735	2.12601052644613
C	7.06885955127900	0.11795374511638	1.38104740870145
H	7.55244808508993	-0.40430541072299	0.55247015652556
C	-1.52371017406472	-1.35718262356548	-1.15734618575021
C	-1.84243551103101	-4.35051658925705	-3.51489133551469
H	-2.49763134100421	-4.65586291073548	-4.33457236591633
C	5.75629748603732	1.45834942601809	3.44487513999038
H	5.21383242899107	1.98371123660597	4.23351328398209
C	-1.15126836738158	2.87680954288900	1.08264024226789
H	-1.11123951889655	3.93727442481969	0.77973043311962
H	-0.56990778286879	2.76721022305481	2.00624917043029
H	-2.20399896123465	2.64663673097398	1.29787477003732
C	-0.22107019940376	-3.48280016748143	-1.41918400236022
H	0.39128536267293	-3.10464962456623	-0.59742035633734
C	7.73525158603122	0.37926590414613	2.57153185441507
H	8.77266416908394	0.05960943538151	2.69982288631577
C	7.07573529238444	1.05170621836652	3.60540001873217
H	7.59853126967359	1.25751304858734	4.54324922443439
C	-2.82199495198339	0.81396187348584	-0.99967654488760
H	-3.64366162256585	1.23772775910516	-1.58956953003603
C	-0.78507480074783	-5.14595714961744	-3.08326854206727
H	-0.58865629921283	-6.10808857628861	-3.56508813845556
C	0.02729206789161	-4.70735017166922	-2.03383446528881
H	0.86289876432599	-5.32724080050612	-1.69545252364384
C	-3.29372512725178	0.63801529257532	0.42125631869174
H	-2.52816902083234	0.27755856971743	1.11966693484182
C	-4.54818362364322	0.78954193680057	0.87293555471644
C	-4.87967183322976	0.53580357326035	2.32196367171249
H	-5.35675194592786	1.41961244028855	2.78225171698984
H	-3.98213211908154	0.28729358797631	2.90716519232494
H	-5.59690086963062	-0.29727390988886	2.42579988170155
C	-5.71684521173950	1.18724622692178	0.00333015385238
H	-6.61264789535727	0.61012123561603	0.28701842899180
H	1.27634612024181	2.22186669200289	1.77696722932305

TS 3b → 3c

O	4.00458022030805	0.60472085797209	0.32378734377262
O	-4.23345773073049	0.07072082557730	-5.33022224361656
H	-3.79298071916029	1.77552708458945	1.09444501561157
O	-3.28023642569396	1.19198456490691	-3.05358341030057
O	2.52361406471018	0.67648644406512	4.81114249338547
O	5.85069661546214	2.48495511492950	0.95514665758168
O	-1.22096899617744	-2.92972826760406	-2.01013274181953
C	4.00360536614166	1.05500214592560	1.43981019023832
C	-2.98012254494039	0.01543376559456	-3.28456978713559
C	-3.56527380566174	-0.59800073397979	-4.57408835808492
C	3.00277147833245	0.71075000257350	2.48580829115656
C	-3.29028579553053	-2.04129453689107	-4.86329424825295
C	4.42540243260205	1.93938578955060	4.24170255060150
C	5.12991055852715	2.04980299062735	1.81706136074389
C	1.31049433980023	-0.35517753609978	0.89595363417960
H	1.51079952841018	-1.40552666982663	0.64764976599995
C	5.29973340906885	2.41601758086246	3.25453861398993
C	1.83613983831474	0.08373521378775	2.19815642532316
H	-3.73113052575370	1.32284862061897	-0.64320708879549
C	0.54715826226308	0.37441964800241	0.07044518076972
C	-2.49348688231520	-2.78847096859175	-3.98947565284210
C	-2.14339534555158	-0.80546267701635	-2.47717319927654
C	-0.03302440862603	-0.23487063173791	-1.17945728691662
H	0.28223054645784	-1.28384050960868	-1.27098473877257
H	0.35352047265347	0.30836700941125	-2.05974427434673
C	3.24842198690460	1.06231385169031	3.91770341447246
C	6.36882610729432	3.24615969819641	3.60984611029022
H	7.02991814346528	3.60003694727751	2.81568212611920
C	-1.89673279356334	-2.18144544748195	-2.73461272747019
C	-3.82795454665451	-2.63243717185896	-6.00938519960904
H	-4.44319372893458	-2.01112737998115	-6.66510840233733
C	4.63224711368465	2.29903581423306	5.57735680926792
H	3.93390012005173	1.91588567078607	6.32415433082708
C	0.18605223958453	1.80726903301306	0.35295249104688
H	0.37416836188232	2.43209184145527	-0.53432987730068
H	0.74403052763647	2.23124399644245	1.20064016979605
H	-0.89049470911757	1.88451791252563	0.57508166826616
C	-2.24347283013097	-4.13197304327124	-4.27503103642026
H	-1.61898383564891	-4.68339401524995	-3.56826860236179
C	6.56753881779444	3.59787332246012	4.93873966804804
H	7.40359970865880	4.24595399636635	5.21337035951417
C	5.69682528373300	3.12196455006235	5.92429574193284
H	5.85225107218902	3.39878951340723	6.97015644199978
C	-1.57652470971563	-0.17284357712058	-1.22224996749929
H	-1.86931746755840	0.88444825548929	-1.28867385496634
C	-3.57558521301572	-3.97229282332708	-6.28870313211596
H	-3.99704484758879	-4.43646104821823	-7.18509346024242
C	-2.78000975111973	-4.72138722673897	-5.41694520357711
H	-2.57860146442499	-5.77522421430645	-5.63254924275779
C	-2.17070154373665	-0.76742351424886	0.03274782760192
H	-1.71319914924933	-1.70927827644939	0.36036947576175
C	-3.20001112575011	-0.28137013441033	0.74189746239780
C	-3.68721058979705	-0.98555510261155	1.98319501149755
H	-3.66554387190792	-0.31176675188363	2.85867041575088
H	-3.07414020856031	-1.86851541521041	2.21640704554426
H	-4.73472416833634	-1.31687171240063	1.86948674090569
C	-3.96941523004053	0.96160078181341	0.36732154113800
H	-5.05332783191191	0.75511261835437	0.39054214048554
H	1.21211618097339	-0.12271607644239	3.08061922080020

TS 3c → 3d

O	2.32053346280367	-0.58061216791968	2.13160867698807
O	-0.06792811627421	-3.47982171443743	-5.27726045360683
H	2.08679000360299	2.21557868898895	-2.42573685008268
O	0.97466562395635	-1.64803143789891	-3.56628757210352
O	-0.06512139506022	0.88286958803347	5.94708511692260
O	4.60086462320175	0.09224146076193	3.37014551571338
O	-3.41506061791213	-0.55052415375332	-2.22295780987082
C	2.22635938973276	-0.04654932806907	3.21406805680265
C	-0.25156201752111	-1.78381717118240	-3.58940215879011
C	-0.81191591787305	-2.80759262206501	-4.59986771004611
C	0.96228729210908	0.25548182599343	3.90088846346340
C	-2.29781042807016	-2.94439050541820	-4.72283321133404
C	2.27592828330426	1.05327449636896	5.96353972624180
C	3.55537081523662	0.28713607914224	3.93782451909216
C	-0.75552258459863	-0.41774415263391	2.09459181461518
H	-1.65587734992564	-1.04465967897540	2.10426309087933
C	3.50097520810942	0.85101411570248	5.31765335303611
C	-0.28255172011873	-0.06783623597553	3.39040565081122
H	1.53713404721329	0.69063487068740	-3.19322110341794
C	-0.38898108305304	0.06759405287410	0.86501902360235
C	-3.14009757947742	-2.17876692177765	-3.90930011107767
C	-1.18217805798542	-1.09739742458823	-2.75769395151888
C	-1.15068971523598	-0.43504275624941	-0.33291417915351
H	-2.19268151477936	-0.07217648763788	-0.27893925134938
H	-1.24060952893544	-1.52969357614695	-0.22973109121377
C	0.96052943852660	0.72877565275382	5.30776277025045
C	4.69806334911192	1.17157161719373	5.96699806753860
H	5.63513533044073	0.99758537058612	5.43349794912303
C	-2.59189267632445	-1.19906141464866	-2.89167221350160
C	-2.83434193160386	-3.84366017422730	-5.64746295488679
H	-2.14062974316255	-4.42436243112868	-6.26046928079885
C	2.25953358405302	1.57742938910775	7.25969344833084
H	1.28892709755061	1.72198664598066	7.73831980695766
C	0.64578533577257	1.11672027807384	0.67477763683616
H	1.57734886854304	0.63181472456734	0.33549278917007
H	0.87246045853874	1.65761537603289	1.60281430934630
H	0.33413891910475	1.81937796461208	-0.10987320103334
C	-4.52292989429615	-2.32076607372158	-4.03495363924520
H	-5.14878167768993	-1.70428728405903	-3.38561316025790
C	4.67372944855338	1.69436721174359	7.25374998323552
H	5.60918707418940	1.94544083251587	7.76028782798993
C	3.45056665614937	1.89724770521972	7.90079272362820
H	3.43052092188822	2.30835437417013	8.91347027975250
C	-0.60975067868258	-0.12535502751118	-1.74234263339867
H	0.47431397289386	-0.30795468480508	-1.74960203787263
C	-4.21386259934359	-3.98173191711023	-5.76760954894065
H	-4.63467877568181	-4.68544355362110	-6.49153498707857
C	-5.05752836925851	-3.21627980169693	-4.95785242226341
H	-6.14274389061651	-3.32179800564790	-5.04996572097140
C	-0.88969235190579	1.29811372992618	-2.17141878431295
H	-1.91964065745729	1.63189975770675	-1.99109629646613
C	-0.04740592727339	2.13405758759255	-2.79687130356459
C	-0.48524273250110	3.52078494184426	-3.19429538371667
H	0.17734536732057	4.28792915330353	-2.75487314744670
H	-1.51558868776788	3.73251108249092	-2.87293287464449
H	-0.43446077677488	3.65415806790445	-4.28921819257265
C	1.37578807539982	1.77721513277396	-3.15010233914071
H	1.64044787340595	2.18940597059281	-4.13792559510473
H	-1.05697152355195	-0.07483104233929	4.16908057045640

TS 3d → 3e

O	-0.89437404499074	0.30474429883435	2.25531041571606
O	3.63595996519806	-2.70345892983468	-1.51421693163821
H	1.71738624083212	2.81203661016410	2.12994746658324
O	2.43363609763910	-0.80612291617831	0.00979253689049
O	-4.42790132811751	-2.69360882699558	1.25563858041233
O	-1.51026371323123	0.06452558818754	4.85166151703282
O	1.32489893853890	1.52640278355104	-3.93638401984924
C	-1.84371120280377	-0.39033624455311	2.53481345563468
C	2.40258398976677	-0.66580991260150	-1.21364244026067
C	3.13584247114544	-1.73950403337353	-2.04807531949890
C	-2.70268571694477	-1.09168268696892	1.56440745795751
C	3.21647759070913	-1.55414956760778	-3.53211005884250
C	-3.95927189207677	-2.22490326938057	3.50650190546148
C	-2.14005004981725	-0.57302258024782	4.04533646082608
C	-1.68824823438201	-0.28062473690962	-0.66637207685749
H	-1.32813188917133	-0.80258410421204	-1.55991970906701
C	-3.20960315782505	-1.52650478596195	4.45965868795607
C	-2.48908975719850	-1.08391049962075	0.19944567184057
H	2.31687692449134	1.35166530461901	1.29222275064787
C	-1.41836155528591	1.05714254734308	-0.60806168207794
C	2.59123442945634	-0.45661238200217	-4.13361216602113
C	1.73430727633942	0.37854304325374	-1.91901384767120
C	-0.46584885765530	1.65019413941283	-1.59685670796683
H	-0.65160928768809	2.72885486039206	-1.70691386713009
H	-0.58115692982698	1.20621684037025	-2.59343181170356
C	-3.73939792566133	-2.05045120078489	2.02766188723738
C	-3.45653126162762	-1.71530132107239	5.82378209455311
H	-2.85095620090678	-1.15262327597543	6.53773635861612
C	1.83403777028885	0.57376489122300	-3.32135811014280
C	3.90894906259449	-2.48567107145473	-4.30972927407072
H	4.37984893592450	-3.32952172116690	-3.79913749103471
C	-4.95424422595072	-3.11201776954827	3.92941250883866
H	-5.52371875037869	-3.64218813430503	3.16345003304741
C	-2.00900218470180	1.99792986866496	0.38582143851544
H	-1.22754029695268	2.28720880083034	1.10854995927088
H	-2.84742215257292	1.56782131886181	0.94898979297091
H	-2.34364538765467	2.91222584282441	-0.12801100941078
C	2.66970347923772	-0.30135854483136	-5.51877719723577
H	2.17152004856057	0.56947336334749	-5.95136038876630
C	-4.44482344247522	-2.59982368672552	6.23635467783594
H	-4.63514993044295	-2.74954052438305	7.30226430420099
C	-5.19480187474698	-3.29950443015477	5.28535963070931
H	-5.97265684322779	-3.99624523413054	5.60878306236238
C	1.00023366440375	1.40741256993090	-1.08853861551337
H	0.93599052222486	0.96996417672999	-0.08196702528817
C	3.98424685245114	-2.32557140427733	-5.69024410581351
H	4.52760934438733	-3.05351508792350	-6.29964752917984
C	3.36098533321526	-1.22936340654845	-6.29350126402816
H	3.41739350052106	-1.09975446690508	-7.37866716273701
C	1.75421948506283	2.70905579383753	-0.98833165057145
H	1.78275549499258	3.29671397671615	-1.91508839873543
C	2.39165821328898	3.17396445027284	0.09625794853518
C	3.10285593995461	4.50317756441595	0.06168109859189
H	2.72280213444219	5.17713003793746	0.85039891441306
H	2.98006486421870	5.00749031836077	-0.90829066842619
H	4.18408576213297	4.38024797908654	0.24991881224942
C	2.46580530356064	2.43393326791836	1.41113851436577
H	3.45470387172459	2.58274339049624	1.87582206850591
H	-3.02847541298887	-1.89529687094719	-0.30685948224000

TS 3e → 4

O	-1.99289425785631	1.36906848259165	1.08643459867338
O	0.77372476888931	-2.57869111358361	0.53739885318845
H	2.15743378560727	3.84110104054843	2.35776685065623
O	0.70592515800239	-0.00500236854736	1.34939550072719
O	-3.11830915377211	-3.09984718883946	0.13186681930472
O	-1.36522277671227	0.60813825517908	3.59392532654774
O	1.15131374977055	1.27853468850525	-3.17074134446163
C	-2.06903437797719	0.16773967326268	1.36297199568556
C	0.87372152921893	-0.22096573026617	0.14216650330831
C	1.00493440222910	-1.70073879210693	-0.26344501386709
C	-2.52567829747207	-0.83709018708983	0.45253569508821
C	1.40627024248238	-2.01628616371181	-1.66722745141637
C	-2.07433803331536	-2.64066780850562	2.19780868197796
C	-1.60375127118179	-0.23710441705942	2.76843938318017
C	-2.04743737037888	0.54802468340189	-1.57509297037831
H	-1.46904550948373	0.38395680711348	-2.49165915484656
C	-1.55090741287159	-1.69446122588013	3.08453832104126
C	-2.47989081369243	-0.60417315240490	-0.93882299475248
H	1.75814987115560	2.18859498419671	1.79513469282110
C	-2.01195893773061	1.85809556747121	-1.05145903779446
C	1.49415507179353	-0.98449357222253	-2.60773732314858
C	0.86656909362390	0.75432506891049	-0.89068955282215
C	-0.78690149243977	2.67611422575516	-1.30938894813425
H	-0.96861519607648	3.73096347199859	-1.05008969347545
H	-0.53721730786612	2.61543188703329	-2.38385499730487
C	-2.63464551828368	-2.24716652223459	0.85912518432126
C	-1.01573746845591	-2.10272523961857	4.30795854676067
H	-0.61992982100001	-1.33466388926352	4.97581346294723
C	1.17464913468376	0.45045136308035	-2.24436378888009
C	1.66244209368903	-3.33790384870785	-2.03895360261975
H	1.56446901250755	-4.11412442916746	-1.27600257431627
C	-2.05562381913759	-3.99370966860964	2.54217159128688
H	-2.46791075877514	-4.70713878629082	1.82573736634942
C	-3.27769286623864	2.56850737996976	-0.68983545684844
H	-3.11367301493878	3.28712316416690	0.12396308285457
H	-4.06915398045966	1.86087575284076	-0.41099976954189
H	-3.60858179695177	3.12331134080028	-1.58692841931986
C	1.84614689863304	-1.28972922581069	-3.92349219782266
H	1.89120762747212	-0.46218175499937	-4.63515195156857
C	-0.98676908149939	-3.45197206426881	4.63985732053763
H	-0.55175855931924	-3.77239158291965	5.59056537175753
C	-1.51084561684227	-4.39912483765126	3.75512907080783
H	-1.48680123869039	-5.46122831788482	4.01461864167087
C	0.46823876824034	2.17001265637321	-0.52900505676448
H	0.18507913589095	2.14244305543759	0.53108885056665
C	2.02483777475261	-3.63427458108452	-3.34956217214177
H	2.23108781409452	-4.66761733144162	-3.64239449589193
C	2.11606501305881	-2.60562980232070	-4.29153171814190
H	2.39519428267357	-2.83625372905856	-5.32405169336320
C	1.59566139519391	3.15737381651598	-0.71351769620188
H	1.72591849251316	3.54369571805471	-1.73209938541476
C	2.45910901531028	3.54993952051776	0.23379152181559
C	3.55400518845787	4.53850086702334	-0.07857762979270
H	3.49557538031452	5.41929241126678	0.58561894271869
H	3.50169410209739	4.89038553875365	-1.11951718044573
H	4.55123103471340	4.09189391958943	0.08154266532560
C	2.43943620433702	3.03901335848413	1.65267021282233
H	3.44915405876950	2.70399183279599	1.94616974506915
H	-2.52307435075717	-1.51954320008832	-1.54001152833409

TS 4 → 5

O	-0.15900746452284	1.53171668122253	1.43442754606868
O	-0.18077700249154	-3.30041379568271	-0.70147770957412
H	3.55259060936208	-0.35478151235280	0.97243936946735
O	1.40331303565892	-1.45663412682729	0.50428071279538
O	-3.74756225551592	-1.55412631222229	1.02484854282358
O	-0.55593269380131	1.48178102776712	4.13917237462792
O	1.21335302057963	1.21994554815742	-3.30032016966274
C	-1.14676179537942	0.75519796003393	1.96952304233724
C	0.91545859656807	-1.17811588048851	-0.56939543157397
C	0.11132644283393	-2.28561120722653	-1.28409917144943
C	-1.95153520846065	-0.00896826415655	1.13631033450923
C	-0.21906440783991	-2.06803497456973	-2.72200804512077
C	-3.12649846752131	-0.92209801691302	3.21408601000039
C	-1.24499107379653	0.78133153168581	3.40544893178657
C	-0.84303174862539	0.92179936504600	-0.80700948868298
H	-1.03338367415035	1.27876203035602	-1.82610168882968
C	-2.28367922830988	-0.13558398696682	4.00896981409646
C	-1.82067398457586	0.05734344934744	-0.26125412295330
H	5.09981216274418	-0.92372044911467	0.31933120674083
C	-0.34574607229246	2.03765719157974	0.11641623461898
C	0.18378672525216	-0.89626297994411	-3.37494316193634
C	0.97490905510469	0.15192972548842	-1.20008472917885
C	1.04093208845330	2.45926158139310	-0.35080024772767
H	1.49117834914554	3.15940904263813	0.37006522364513
H	0.95884925131401	2.96055366136650	-1.32863554577991
C	-3.00731468860457	-0.88595517108279	1.71887410548859
C	-2.40800926636312	-0.18483747560282	5.39736565869699
H	-1.73582632008074	0.44336094983523	5.98632121979082
C	0.87965013465790	0.23160481028483	-2.65363763975151
C	-0.89647769264781	-3.06985833681002	-3.42563564705143
H	-1.18928732330888	-3.97040890350361	-2.88119846653595
C	-4.08369117481312	-1.74611946115663	3.80745478197805
H	-4.72320111102628	-2.34537711844201	3.15543674897905
C	-1.34488749557056	3.19407957605924	0.14733732123906
H	-0.98488749045990	3.96727854166983	0.84256480428372
H	-2.32662331044185	2.84068036675505	0.49659958710311
H	-1.46566551762246	3.63771641831555	-0.85241335291288
C	-0.09958456701955	-0.74026421952938	-4.73572505257850
H	0.22609553288618	0.18575155875991	-5.21437488428258
C	-3.36201018797869	-1.00994311278220	5.98847335991702
H	-3.45240200627436	-1.04543367626811	7.07809140422620
C	-4.20249523631527	-1.79274906806372	5.19421069334840
H	-4.95061522506130	-2.44123214130289	5.65862973193620
C	1.85468760871735	1.16938270899600	-0.47416101704569
H	1.96365836307118	0.77806196608829	0.54412852350007
C	-1.17554199034287	-2.90550781614860	-4.77599227120092
H	-1.70710738405034	-3.68781952608608	-5.32440407832169
C	-0.77368836170447	-1.73652024317151	-5.43168268908138
H	-0.99294367718879	-1.60487468461895	-6.49496767899389
C	3.21767385335557	1.36668622924463	-1.08373003535867
H	3.23776406320896	1.97502639286607	-1.99509585718906
C	4.37897017029201	0.86499277150257	-0.63745379711430
C	5.67017361370576	1.16233947467198	-1.35786924447575
H	6.41073766824694	1.61340578169711	-0.67415059182373
H	5.51766395711755	1.85055601780180	-2.20186245237521
H	6.12750635579253	0.23668721446858	-1.74940914302931
C	4.51869788472351	-0.02088992847368	0.57383259579889
H	5.07518186409986	0.49648355646249	1.37496472735515
H	-2.48306530273348	-0.53864074205331	-0.88971119556684

3a'

O	0.94808680911753	-0.19799880629470	-2.39145155211445
O	-1.80688477930634	-3.18730649807647	-1.51508094908646
H	-6.31639022169370	3.36720763588621	1.31979262028367
O	-3.34744936640080	-0.95083351382835	-1.82836104262270
O	3.06437420031022	2.53095171148483	0.85281023473888
O	3.10524280214453	-1.80882059032884	-2.32944636498765
O	-1.33453951813987	0.80637261578232	2.04644926308761
C	1.82166327927753	0.06574102741643	-1.59572585205553
C	-2.58637833813514	-1.01538802514695	-0.85797518375918
C	-1.62462068709471	-2.21897264028182	-0.81423956298576
C	1.82502579564064	1.23352159558374	-0.70056859677771
C	-0.44974666446847	-2.13465357147801	0.10697978282785
C	4.04469319362305	0.46274919724399	0.31873435933607
C	3.03121211999061	-0.90043293139597	-1.54042148205561
C	-0.56059317776296	1.96287993663764	-1.12376832745432
H	-0.74073903540992	1.19856277027005	-1.87960891223225
C	4.07433291494327	-0.67433605625719	-0.49663725893765
C	0.74307088702162	2.07266957098004	-0.55859995931261
H	-6.34866899165568	2.16144373643974	2.61578146799266
C	-1.62606669066460	2.61616239290089	-0.57231742964761
C	-0.39482523122409	-1.11772306298946	1.06500896395834
C	-2.54104194803397	-0.09155289749451	0.22388622617163
C	-3.00628502281204	2.13896512989909	-0.88674649105482
H	-3.04429269138923	1.62061811673182	-1.85627286832549
H	-3.71880531626306	2.98064409444686	-0.89905758762326
C	2.97199749267423	1.51074634036725	0.19217335676854
C	5.08597862446887	-1.62640686349236	-0.33591644506453
H	5.07802293704930	-2.50017154862675	-0.99142250928221
C	-1.47762464823209	-0.06524539063107	1.17035821009616
C	0.58934338485641	-3.06159036799382	-0.00149577649975
H	0.51191512861860	-3.83403277181410	-0.76979837225948
C	5.02945700354550	0.63567745421192	1.29587419341122
H	4.98144547505258	1.53478965836846	1.91347190522001
C	-1.51149151779793	3.64023649345186	0.51919618515872
H	-1.51043244082891	3.09699853133660	1.48033975117495
H	-2.37717245833993	4.31838672371893	0.50725722970118
H	-0.59341223901496	4.23994584907720	0.46128518770780
C	0.71439785173529	-1.03529654458783	1.91044041382764
H	0.73005273125119	-0.22002936962553	2.63730619305351
C	6.05405356394962	-1.45495495801994	0.64578799473211
H	6.83673557721472	-2.20611183199720	0.78056954759370
C	6.02386266275360	-0.32086608406599	1.46427341428441
H	6.78392910091943	-0.18493082120356	2.23834439151399
C	-3.46110594943266	1.10772758888677	0.21323966671746
H	-3.32930374475518	1.59181916603256	1.19124745491607
C	1.69618993539370	-2.96832248070895	0.83677805140351
H	2.52192168751887	-3.67837257113622	0.73653285276198
C	1.75642823058506	-1.95191887197118	1.79518458721860
H	2.63329725923906	-1.86753756501953	2.44439527498939
C	-4.91913811402117	0.77311231022419	0.02212208109071
H	-5.11543207586611	0.00360343596146	-0.73157595340549
C	-5.95694648581473	1.34455861082439	0.65144011164097
C	-7.37241157388860	0.91762613817752	0.35400367344962
H	-7.98612411965500	1.77024768266568	0.01217818810531
H	-7.86633894818709	0.51837449244407	1.25779398343081
H	-7.40370433697897	0.14042198197843	-0.42277871232681
C	-5.83493196384858	2.44043654863180	1.67955962902485
H	-4.79180438916764	2.67731067706761	1.92580913415480
H	0.90824747848912	2.86592607943598	0.17744218492662

3b'

O	3.36261924634009	0.32025900749559	1.06183924940922
O	-5.58244569322342	-1.83671046196126	-3.33848875820868
H	-0.07140717370069	0.93312780842650	2.48716370122985
O	-3.66313797317598	-0.20172352582635	-2.32504982582501
O	2.05941001709758	4.82881534835056	0.38497386636380
O	5.48774253800251	1.20701867992881	2.44426689789788
O	-2.17532371522389	-3.78570353642713	0.35945985599531
C	3.48006994316183	1.51989968831715	1.19058593323382
C	-3.72383951495783	-1.36649129150060	-1.89955000706607
C	-4.81859501728264	-2.26521894787049	-2.50300037642051
C	2.56635629741154	2.52551032584916	0.63548225056633
C	-4.91111647521496	-3.68314241194158	-2.02882844635293
C	3.97551097098892	4.39997435788333	1.67519721362256
C	4.70483829619153	2.01219242623840	2.00487415666293
C	0.93477557991982	0.92030784794689	-0.45849134201124
H	1.45953875874829	0.03313254982721	-0.10333762216503
C	4.87677468859346	3.47833272041669	2.22229552633155
C	1.44779568324455	2.19497947159985	-0.10744475538560
H	-1.12509554899228	-0.14487568433591	3.41853288635294
C	-0.21441215243643	0.76427013301275	-1.18952788726613
C	-4.01607846144582	-4.16095712835359	-1.06533585222582
C	-2.86657557378103	-1.92307745607168	-0.91992639618670
C	-0.76995842546380	-0.59993873203010	-1.43764444679569
H	0.03207279888722	-1.35141910785818	-1.47182143285699
H	-1.29766716665093	-0.59106461267528	-2.40106050022500
C	2.79218535688010	3.97117819409228	0.84733410448465
C	5.96098330582431	3.92546831472825	2.98538805271640
H	6.64402129129974	3.17816616190038	3.39535066950240
C	-2.93483822816695	-3.27882014872401	-0.47266399066070
C	-5.89474218444252	-4.52383594190373	-2.55599935124414
H	-6.57297771057276	-4.11090525006262	-3.30689248911035
C	4.16927653136143	5.76642803998519	1.89837812272584
H	3.45024177819741	6.46082923906065	1.45899813802576
C	-1.01860931413658	1.87781017365697	-1.78743756909630
H	-2.07292763694043	1.57132397504115	-1.85492803987835
H	-0.67541331305415	2.02371849281345	-2.82684889917300
H	-0.93021372087482	2.83560595875918	-1.25946011706132
C	-4.11728659676193	-5.48535645708716	-0.63623753578673
H	-3.40189492956744	-5.82240792340480	0.11745134504980
C	6.14478853435149	5.28468220399253	3.20574322332719
H	6.98822088415949	5.63200414730987	3.80804593883118
C	5.24571917560976	6.20647661632769	2.65994830660962
H	5.38791946364225	7.27635431136401	2.83380738806214
C	-1.80017108656266	-1.03598825866073	-0.32397748360866
H	-1.24506407329159	-1.65986207813947	0.38927009895205
C	-5.99095361199367	-5.84326555882399	-2.12423423037067
H	-6.76144676657450	-6.50167924662860	-2.53560226998266
C	-5.09838115564296	-6.32261340327244	-1.16140973090817
H	-5.17153348131035	-7.35905405227726	-0.81843898493533
C	-2.42125200059094	0.11748668596478	0.43423492221192
H	-3.30000335574009	0.56450937544040	-0.04371411333853
C	-2.01975332181835	0.62249057554315	1.61068575418693
C	-2.76191678944405	1.77093167519496	2.24634220125833
H	-2.09728893311083	2.64100041673284	2.39119675723898
H	-3.14256397512281	1.49583507034317	3.24577710895362
H	-3.61528162771914	2.09128034171138	1.63139453089252
C	-0.82866673107279	0.13533488190643	2.39308149584898
H	-0.33958677858612	-0.73210020456894	1.93319918502948
H	0.88783451583508	3.07210486334416	-0.44836585082795

3c’

O	3.99762324080327	0.30064945197301	1.06315168265197
O	1.76176148899140	-5.07603247571153	-6.15390521258879
H	0.78956018630948	2.76393363601137	-3.10624866189752
O	2.69829252539307	-2.91125417415068	-4.81027534001623
O	1.21110492053140	4.12005163867734	1.09933194019296
O	5.91591889379834	1.75469660930073	2.24930212069831
O	-1.74677668495776	-1.87009590129120	-3.60461293017881
C	3.77524824180007	1.47848846519333	1.23718156139836
C	1.47568035068529	-3.10091159228462	-4.82223645320459
C	0.98050168903369	-4.34830262909711	-5.58392495783289
C	2.54968519297742	2.17774341342485	0.82530306174339
C	-0.48955643248261	-4.63464137350512	-5.59918109429963
C	3.33797935067016	4.28169085303516	2.08008098421059
C	4.86051326908274	2.28103566634188	1.99737864599815
C	1.38722241126113	0.35478459255463	-0.58440757798661
H	0.36881867621215	-0.05159054668856	-0.61565858170717
C	4.56517565613315	3.68692291226619	2.39650944903449
C	1.50781397315504	1.56915899980619	0.15125095026821
H	-0.29302823149917	2.55878252697005	-4.49327120874413
C	2.28154136989054	-0.23545011607005	-1.43491343714902
C	-1.37608053308435	-3.77907455073492	-4.93638721281317
C	0.50500492471596	-2.27098547341537	-4.20264794949195
C	1.83317159136209	-1.42582643887044	-2.22925336487580
H	1.22281501588963	-2.08831411669604	-1.59634934974135
H	2.70076634839204	-1.99271412894221	-2.59875672976058
C	2.26756656421520	3.55335942306652	1.31141760797656
C	5.53331886900368	4.40689396482522	3.10484863908362
H	6.47817108835281	3.90917662487330	3.33421940206277
C	-0.89357371652254	-2.55352125275442	-4.18651027104104
C	-0.96789048006225	-5.75774255351240	-6.27903299951921
H	-0.24046430476378	-6.39924461196843	-6.78274797063084
C	3.08914989184755	5.59849414486305	2.47913844896823
H	2.12393705894770	6.03708585158649	2.21804929111917
C	3.64107378415386	0.29119277907900	-1.72971789253199
H	3.89423510278264	0.10680251700174	-2.78254811548592
H	4.36647671154145	-0.24891691975405	-1.09684737665138
H	3.74419503331983	1.36250748036527	-1.50482442220622
C	-2.74332646620100	-4.06022627204407	-4.96214732056644
H	-3.40475095596039	-3.37097299981127	-4.43223089004184
C	5.27877167981484	5.71463549862519	3.49800957240826
H	6.03487950501679	6.27663250553483	4.05224380044155
C	4.05294971713303	6.31036386069991	3.18381643400063
H	3.85174975683795	7.33963484979188	3.49224459291203
C	0.95770565919287	-1.03465782989895	-3.45965766349135
H	0.04074970831220	-0.57193192894636	-3.06898716017593
C	-2.33192329282183	-6.03236443246303	-6.30196688833324
H	-2.70720151037348	-6.91113997127439	-6.83429425230072
C	-3.21985024809443	-5.17875802179065	-5.64110656252560
H	-4.29326803202752	-5.39067706313890	-5.65705134059444
C	1.67072193285340	-0.06163302324418	-4.36476290861426
H	2.48359289852614	-0.50950523195768	-4.94953203253352
C	1.42241998238369	1.24851384475970	-4.51318525114005
C	2.24719244346729	2.08343212313635	-5.46070267209448
H	2.74434536366140	2.91430910043812	-4.92882987366904
H	1.61608595072925	2.54335907223296	-6.24155720528265
H	3.02230393449987	1.48281705825411	-5.95820319213968
C	0.34686560660127	2.00998172181744	-3.78113848345222
H	-0.30139206330350	1.35848935545420	-3.18184660631610
H	0.57869539187177	2.15177508805659	0.21161122845727

3d'

O	-0.34610819833472	0.36773404466655	1.75871274825506
O	-6.64559095034694	6.41889670918272	3.92577477775401
H	0.42987973070001	2.88257334669958	1.32585769845780
O	-5.41711494754660	4.07621768246603	3.31942588872337
O	-1.71824216022633	-3.79639972889259	3.48379325936121
O	2.11057723191994	-0.66192713172650	1.34984891189423
O	-1.26513032301971	6.15192230597286	4.20757526695027
C	-0.13151588727153	-0.75764581050178	2.14874992039663
C	-4.70529513354484	5.03342054812570	3.64467542146341
C	-5.43741011857578	6.35436877746375	3.96600278302366
C	-1.08031970101913	-1.57810421088786	2.91720430682144
C	-4.61529371118670	7.55441314749072	4.32454899478028
C	0.40804304166934	-3.63483350788609	2.50052135559097
C	1.23481359700663	-1.37516518358226	1.77222081702904
C	-2.80885847180068	0.19129100989551	3.66966868681825
H	-3.90334776512056	0.23431670475100	3.66426169825250
C	1.41721107495940	-2.84560420016303	1.93567892667067
C	-2.27664188718190	-1.11073096507276	3.42324027924344
H	0.08164224240774	4.42448235233465	0.52555667825160
C	-2.17778340928304	1.32774155148923	4.09323698084282
C	-3.22209909306360	7.45050297349543	4.39662568437341
C	-3.29036298782616	5.01293325051293	3.76319369907098
C	-2.98342835905707	2.56468862545392	4.35455545289166
H	-2.84175606851102	2.87578022133849	5.40429611844208
H	-4.05490925770140	2.38543212540785	4.18936220875782
C	-0.88440974910433	-3.04931022541033	3.00517146765719
C	2.61179164365180	-3.43314987632531	1.50623269794380
H	3.37660918003160	-2.78485841413075	1.07275123032887
C	-2.50186716313743	6.14776158670380	4.11379429931546
C	-5.24656323781163	8.77255797677865	4.58838517373073
H	-6.33675879584478	8.80950121293637	4.52246153763344
C	0.60365972186000	-5.01352410196489	2.62624808979589
H	-0.19945207553877	-5.60244945861907	3.07383088957976
C	-0.71577894556364	1.42925132648183	4.35362734679143
H	-0.51390232288976	2.14602873705156	5.16217023580182
H	-0.23002630539451	1.80903660957107	3.43863915700536
H	-0.25371516684545	0.45897891216667	4.58768208339579
C	-2.47032196926516	8.57737992526080	4.73450496177868
H	-1.38557690641042	8.45678201358515	4.78053176645700
C	2.79759253045285	-4.80392875300651	1.63292345322004
H	3.72911278164116	-5.26274026679339	1.29187285146982
C	1.78951396664650	-5.59495734660882	2.19320594762381
H	1.93460699889675	-6.67372949445784	2.29352799841744
C	-2.53455184895033	3.74198290378615	3.44669398692049
H	-1.48573393041028	3.96906927614675	3.67929135833700
C	-4.49172548611161	9.89325192862815	4.92173771223656
H	-4.98498008232221	10.84822317887740	5.12448844722659
C	-3.09963552194075	9.79191674235255	4.99448733603471
H	-2.50195435920195	10.67038371322250	5.25623870102612
C	-2.64917455865613	3.33097660336492	1.99939450133996
H	-3.65291526680459	3.00342148699300	1.70132647721921
C	-1.66981294880479	3.30510766310814	1.08459072244898
C	-1.92318145427827	2.77232492177504	-0.30160675778010
H	-1.27187048519698	1.90266575035755	-0.49535714339339
H	-1.69375803912526	3.52758016767646	-1.07400180252946
H	-2.96813953692534	2.45419724155773	-0.42938402451624
C	-0.25121721053574	3.75135824655443	1.33384463088667
H	-0.13840135687724	4.29853293740869	2.27950450764778
H	-2.95858961727822	-1.93015984306310	3.68698124483143

3e'

O	-0.71712425854624	1.09619655233332	5.05695776800950
O	-0.45529084201959	-0.26082948400254	0.07434757601256
H	-3.78776630756716	6.01907874427234	4.77729330626995
O	-0.71960212751726	1.46685271388557	2.07795378830569
O	-0.73391366647664	-3.27056182276144	3.55690577323708
O	1.69371665374005	1.51228107426478	3.86106501802321
O	-4.75360365816690	2.91791437006764	0.07606480805649
C	-0.32094645456962	0.29608097533246	4.24650468854734
C	-1.59028770719358	1.51959733778031	1.19409909111734
C	-1.33971182791607	0.56382635192272	0.00936986139636
C	-1.06152219723049	-0.93466097494824	3.86317489416734
C	-2.21143941330925	0.67052404831007	-1.19608365894792
C	1.06763671744973	-1.90169769370709	2.86740564666972
C	1.07964226521140	0.50083788734800	3.63777094091881
C	-3.33695008475401	0.08218290750180	3.36613340131908
H	-3.77263311606861	-0.01622581898614	2.36310383697401
C	1.67285640979833	-0.63855702358277	2.87447651257319
C	-2.39466960977685	-0.99919338826136	3.60169710784002
H	-2.91842382989536	6.88205284119644	3.49892988081404
C	-3.65315422976930	1.21374521554634	4.03027562249065
C	-3.33355172184102	1.50167820004106	-1.15153916949673
C	-2.77251711504044	2.30009599710811	1.19828330142283
C	-4.29239450744827	2.33168768147768	3.24873654842547
H	-4.78969392578517	3.04024429643617	3.93217299127166
H	-5.05520748197435	1.95495463553650	2.54959541854865
C	-0.28124176155200	-2.14466457108375	3.48323821757896
C	2.89866360349161	-0.45117916298419	2.23220466546311
H	3.34586549203254	0.54448043516018	2.26529345721327
C	-3.68134390052629	2.30000157565477	0.08800432442236
C	-1.92030611595139	-0.07026740264396	-2.34381495386819
H	-1.03926655060578	-0.71668987024190	-2.33071964656701
C	1.70222913149267	-2.96932567268479	2.22598125665477
H	1.20610209068688	-3.94199176168840	2.24056207427150
C	-3.45128013040678	1.44875925930800	5.49735483434241
H	-4.44688399777404	1.58053492228820	5.95787004289573
H	-2.88274581661790	2.37394326859923	5.66815247985260
H	-2.92905058313392	0.62316314062489	5.99219597628111
C	-4.16373250999610	1.58686968083986	-2.27066295027846
H	-5.03932939231847	2.23586330492485	-2.19780227908773
C	3.51346659282319	-1.51055603587608	1.57733266239007
H	4.46597506331737	-1.35702308185490	1.06303979240504
C	2.91609093110164	-2.77480248422506	1.57902769816691
H	3.40165420373255	-3.61016227660780	1.06735639530904
C	-3.24447833882118	3.12447768157030	2.38688045231611
H	-3.83727761771986	3.94174908821933	1.94824251288720
C	-2.74344423116627	0.02944202511567	-3.46111444008734
H	-2.51489148161804	-0.54292250532733	-4.36457657814605
C	-3.86741166753132	0.85989692074094	-3.42082944679501
H	-4.51867559197577	0.93626458087359	-4.29666151242375
C	-2.15568670311068	3.70280538507038	3.25775392418800
H	-1.28442825330663	3.06132078785165	3.40895300625487
C	-2.17554946591768	4.88782218531672	3.88615915587627
C	-1.02307733643401	5.29084707238685	4.77204610702863
H	-1.36422168344771	5.50276374381377	5.80148751715841
H	-0.53979731836922	6.21426191901710	4.40524669060404
H	-0.26558305879669	4.49544537441610	4.82042451582563
C	-3.29837845764445	5.88885510194875	3.79575664119902
H	-4.07016147481872	5.59213841681317	3.07292154722420
H	-2.71782847245066	-1.98604279944800	3.24587657547771

4’

O	-1.27482956337556	0.95549998678648	3.17537203336912
O	0.04032029581919	-2.32865896075490	-0.54565677614067
H	-1.00277811966364	4.74068528964373	-1.89416166401001
O	0.20263772851133	0.15544543931387	0.44943570712579
O	-1.31327669732049	-3.72242050499037	2.61783222612701
O	1.37820945989598	0.86024058058214	3.30855180480743
O	-4.43259680236169	0.63828531329373	-0.31824514997018
C	-0.70918166052409	-0.22432219636616	3.03066494075288
C	-0.91089646281341	-0.21335507876559	0.04837601922065
C	-0.97148689364921	-1.66733060955587	-0.46869402558611
C	-1.39660561463057	-1.39167914370853	2.91703604302716
C	-2.30047983922867	-2.25397364687260	-0.81431282586378
C	0.78734107296638	-2.62828509310880	2.55360665518896
C	0.79232172310185	-0.15519334593132	3.01664534497206
C	-3.45859234068377	-0.13108588187659	2.90361052889371
H	-4.54302826091320	-0.04075804115352	2.80071469094245
C	1.49664971071373	-1.42930190937131	2.68535359040426
C	-2.85504548220289	-1.32128240955462	2.88398038694535
H	-0.24001144100006	3.24359619013647	-2.45741046088866
C	-2.70713768383163	1.17395419898664	3.06101176769767
C	-3.44154269271575	-1.44884541200223	-0.77092089275876
C	-2.11158787418170	0.53645441469732	0.06794018669126
C	-2.95601239274213	2.15205360128854	1.89809073944199
H	-2.76678756414821	3.17685410885042	2.26116667401647
H	-4.02938292808275	2.08592468763877	1.66200782088229
C	-0.70640257011372	-2.66805819636733	2.69966065094464
C	2.88215602268196	-1.41589874798683	2.53117140765490
H	3.40320911411669	-0.46231004654754	2.63823184649774
C	-3.37058298192582	-0.00163056600059	-0.34068430102095
C	-2.39696855652198	-3.60142520588923	-1.16774631246402
H	-1.48155681470568	-4.19758441528438	-1.17250620519760
C	1.47017819585729	-3.80635843521442	2.25472723952008
H	0.88635293809690	-4.72207197890204	2.14267792107066
C	-3.11421279163397	1.82763975018071	4.38415949363680
H	-4.17001909358842	2.13628119049550	4.34752610894486
H	-2.49124872007378	2.71539472273160	4.56928155500979
H	-2.98247999295078	1.12019542790605	5.21641475274058
C	-4.68082956963190	-2.00113082284868	-1.09818632553227
H	-5.55135602269929	-1.34293918654236	-1.05143193915860
C	3.55868835174033	-2.59318828871334	2.23008529103137
H	4.64352036264983	-2.57946258397889	2.09406080729390
C	2.85141884614495	-3.78958533755503	2.09104096691183
H	3.38308764342987	-4.71263918650658	1.84433612748959
C	-2.16624485960907	1.96991285847269	0.57461097505952
H	-2.79195465142763	2.50597084195340	-0.15796683329897
C	-3.63537252334974	-4.14853758958907	-1.48799114857156
H	-3.71575923112551	-5.20559486205293	-1.75668302748368
C	-4.77752396278478	-3.34326966803965	-1.45657584193063
H	-5.75261680944794	-3.77169248120788	-1.70762630286507
C	-0.83127257602965	2.67472805163680	0.64120966678897
H	-0.26245917500911	2.53613268407285	1.56540360819581
C	-0.28701042164891	3.43978070691967	-0.31430016360208
C	1.06323594765494	4.07711524256832	-0.10953041946486
H	1.02210923946372	5.17302255175984	-0.24807148390229
H	1.79463238996619	3.69209989181392	-0.84208925354095
H	1.45594290079473	3.86692664326355	0.89552433582185
C	-0.89518249088760	3.66337453160735	-1.67417659191307
H	-1.87877156863777	3.18787975575735	-1.78341901492732
H	-3.40748624573331	-2.25596882911849	2.77195704497260

5’

O	0.81225712090793	1.68688909377297	1.42556050521055
O	0.66390318616328	-1.93816130567152	-0.94786969238815
H	-4.04151187885531	4.47255558370619	-1.57703706707184
O	-0.52744924797188	0.57869117246183	-1.79644053760416
O	2.33355973711719	-2.75953103114743	1.21750613981168
O	3.27219327507716	2.37352841513937	0.55474778496061
O	-4.14628514113016	0.33418025143400	0.29602896317263
C	1.67531533859512	0.70480374903527	1.07379974793556
C	-0.81692299702732	0.02146809801547	-0.77817944154615
C	-0.34694622872056	-1.39640598940081	-0.32131156614080
C	1.35835953609443	-0.60226663550392	1.09703426577992
C	-1.65521112095422	-2.21845013195312	-0.33274476489226
C	3.48996732114326	-1.18573198827730	-0.12613695420693
C	2.96000073837487	1.20161686689172	0.51416045471005
C	-1.00827552591666	0.05506784755383	1.63192155819587
H	-1.73923942742032	-0.29536918465564	2.37787095780000
C	3.81010034078693	0.17677850797389	-0.17720942242695
C	-0.04585852782132	-1.05595624069920	1.22781674830671
H	-3.73436729468946	3.19736799597982	-2.76488466137571
C	-0.33752526866858	1.31771263789790	2.19963098074250
C	-2.91745935240808	-1.66350375587869	-0.06533403707906
C	-1.74187703249238	0.56319981346292	0.34638789614164
C	-1.37075365935090	2.40285738033309	1.92116279735658
H	-0.91708561882799	3.40355211742785	1.98291688592061
H	-2.16048823177014	2.33971509229725	2.68693360099716
C	2.33545036240935	-1.65402162432876	0.70989648759323
C	4.94001691199597	0.60040196031264	-0.88229597706734
H	5.16630004647477	1.66910494099575	-0.89879667651117
C	-3.05969785938812	-0.20146275433917	0.19706919187160
C	-1.55726356683814	-3.57653847746036	-0.64544410884659
H	-0.55247083620141	-3.93936187247694	-0.87939433843134
C	4.30636669767295	-2.11606761984627	-0.76648841668227
H	4.03746438397687	-3.17135757192979	-0.68793583708872
C	0.08429372489718	1.18759249772001	3.65745362476638
H	-0.78076765370536	0.95157361969305	4.29492243325226
H	0.53342178742547	2.13030239434010	4.00308305095213
H	0.82846273930466	0.38579558634200	3.77713502402858
C	-4.06408246088820	-2.47218410850201	-0.09567594732848
H	-5.03147586275422	-2.00276991394138	0.10028068471347
C	5.73740321016228	-0.32783177724425	-1.54249881784475
H	6.61402704468094	0.00512881626729	-2.10502559381935
C	5.41733795428490	-1.68798474245680	-1.48563493117669
H	6.04239986597835	-2.41702481467762	-2.00905211743780
C	-1.95556193488327	2.09034295014654	0.52255721591303
H	-3.04018487126396	2.24633627916461	0.55473338494005
C	-2.69340578058282	-4.37504088804741	-0.65621816441297
H	-2.60771658655751	-5.44097514191762	-0.88942505246594
C	-3.95398775836108	-3.82527845613294	-0.38017473640403
H	-4.84671993985512	-4.45670666989448	-0.40075167614094
C	-1.37016327951893	2.97240433668845	-0.54805636875749
H	-0.28099894266872	3.08906315220013	-0.51358277611726
C	-2.04886116455996	3.62289913199102	-1.50141903762660
C	-1.32878278549076	4.49973820072375	-2.49204910065640
H	-1.75332472447050	5.51928471615158	-2.50833577574530
H	-1.42859802825231	4.09490172516899	-3.51396478785535
H	-0.25664797322009	4.57475009598208	-2.26228659464383
C	-3.53469790791733	3.50213472222508	-1.72333475961807
H	-4.01126785313878	2.75806399036735	-1.07184932703259
H	-0.11194026158200	-1.97138278878040	1.83034336617045

TS 3a’ → 3b’

O	2.89716052325622	-0.22890041430310	-2.15728380345045
O	-2.85838948207387	-3.90232728613493	-2.71269367043208
H	-4.15726465560873	2.35202784130695	2.33064422693361
O	-3.16942240265868	-1.19342108292847	-2.69028958772279
O	3.74119996516990	3.06809195213732	1.14424644961597
O	5.54598555294309	-0.64720616277432	-2.32964434618149
O	-1.80984744892252	-1.17102405998999	1.82386319371006
C	3.61215521809800	0.42898575825421	-1.43539005658181
C	-2.77321771763895	-1.74792657638627	-1.65985158828601
C	-2.62719508241820	-3.28257493144288	-1.69941138334324
C	3.14673340755296	1.45895919929545	-0.49442736549754
C	-2.20895168646559	-3.98497496301228	-0.44383034059681
C	5.56586420352378	1.89366353929202	0.24394361257376
C	5.13656338584987	0.16507322774170	-1.53835593256586
C	0.66490400701252	1.27838347529241	-1.00292376851740
H	0.77955247327184	0.41052818160936	-1.65224820001322
C	6.05694413039874	0.93496555773618	-0.65098076225078
C	1.81973735578681	1.80836041280008	-0.36074099291413
H	-5.07221183398904	0.88864812249401	2.71243670499457
C	-0.58265290586876	1.81484012351551	-0.83977117721242
C	-1.92657968727834	-3.24886319445414	0.71246369177325
C	-2.43132591177017	-1.07991890797198	-0.45448064917289
C	-1.76908273416374	1.17249879852873	-1.47805996745820
H	-1.48132088209916	0.47788340369375	-2.27602691272146
H	-2.39665078522363	1.95492021768524	-1.93675902335869
C	4.09764991786473	2.20772061318492	0.35793644583602
C	7.43176169156635	0.68633055923858	-0.72741343434836
H	7.77875580265676	-0.06686856261390	-1.43812464046105
C	-2.04726619631222	-1.74023674019683	0.74664437080222
C	-2.10306702201334	-5.37783816537181	-0.43548973788803
H	-2.33257937440461	-5.91443909568837	-1.35943550110566
C	6.45928160669258	2.59570428128255	1.05840398440716
H	6.04674606042391	3.33652283662745	1.74616714505813
C	-0.89072684162058	3.04159986021669	-0.03177730537798
H	-1.82253544021201	2.88907575673903	0.53260540002637
H	-1.07310111521703	3.88283514268846	-0.72114028407640
H	-0.10086285400715	3.33832513071951	0.66836308562917
C	-1.53762095347605	-3.92123346869806	1.87211726620474
H	-1.32710991318983	-3.31243885067436	2.75431279515299
C	8.31309567375550	1.38821452388363	0.08481818408242
H	9.38668390652349	1.19243329853085	0.02348554035999
C	7.82411721470675	2.34466640218360	0.98035124868504
H	8.51603525998721	2.89853069987608	1.62031202610166
C	-2.60196526845966	0.41747899466016	-0.38507886811008
H	-2.19984717416873	0.69378072410361	0.59978821439772
C	-1.71661028566018	-6.04296272862264	0.72419978373043
H	-1.63478391197936	-7.13361245781482	0.73097916890246
C	-1.43242209590369	-5.30965790373606	1.87937466449181
H	-1.12671254914883	-5.82864624419694	2.79281070191431
C	-4.05417779772293	0.82349298821776	-0.43564256252194
H	-4.51546099126316	0.74425984123135	-1.42808449421780
C	-4.81132722326897	1.21699387293311	0.59914942088601
C	-6.25819130785487	1.59209920933926	0.39915262690892
H	-6.45430354484006	2.62344167057938	0.74380955286412
H	-6.92376134237062	0.93455113276497	0.98567388052759
H	-6.55425270093824	1.52284931568333	-0.65771976254975
C	-4.31671915284312	1.30217904955210	2.02337891768448
H	-3.38874246280951	0.73506933215690	2.18269327032290
H	1.66733337882310	2.61908674923592	0.35895054435644

TS 3b’ → 3c’

O	3.84020771928148	0.27123448221487	-0.57408543823932
O	-4.27177477672552	-2.85939652036877	-4.26914630022155
H	-1.54368563620479	1.44274329368059	2.50187590386327
O	-2.92956958534941	-0.78337896193299	-3.15308779797523
O	2.90297543897126	2.84968153804485	3.26933097567269
O	5.88012051620626	1.96588672223060	-1.15298537136766
O	-2.01014012235716	-3.52923248327682	0.58059491962467
C	4.00405264578104	1.23529291604255	0.12747949727150
C	-2.99877067856005	-1.83765104166594	-2.50926080958858
C	-3.75552649413087	-3.00186421957261	-3.18339288530580
C	3.10178377967339	1.62773589427046	1.24347068350515
C	-3.83486855835079	-4.31186998981130	-2.46179916745326
C	4.84128539464752	3.34930610620804	2.03146182586123
C	5.24436717528057	2.12277822980071	-0.14146143925001
C	1.13305050607189	0.19033729586092	0.49390090197131
H	1.18720968096624	-0.86145861228826	0.80633070714282
C	5.62724878057269	3.13187812681847	0.89053429099406
C	1.84580719480788	1.13425630447302	1.36771705535908
H	-2.95270281236700	0.46781450463589	2.94794317613944
C	0.37116153610089	0.52096342365222	-0.55800708657007
C	-3.24486867804016	-4.45093074974686	-1.20124594228912
C	-2.45658261708668	-2.05605786381810	-1.21536189375106
C	-0.43264104941452	-0.53487374538510	-1.27871019846182
H	0.17153001861154	-1.44739215271289	-1.40052492538175
H	-0.71484182449769	-0.18734588422180	-2.28422966708698
C	3.55112291824777	2.61520863788490	2.27069858316171
C	6.80714394990160	3.86119736125187	0.70557953328586
H	7.39425083872838	3.66794543984596	-0.19496346482797
C	-2.51388497640918	-3.30512809299098	-0.52899696166379
C	-4.50145602635891	-5.38838878363275	-3.05256795318061
H	-4.94790699244172	-5.23464291835312	-4.03824189297098
C	5.24756417903473	4.29526998914253	2.97803271127570
H	4.61403513991752	4.44643065485229	3.85442780115622
C	0.22999836509011	1.93372783643307	-1.05118917586308
H	-0.83618503106690	2.20382898351389	-1.10111630738463
H	0.63048604805112	2.01346044402036	-2.07514709535496
H	0.75104686277354	2.66878216498418	-0.41937956866240
C	-3.32899226891786	-5.67838141051485	-0.54114909776579
H	-2.85520088580180	-5.74928984211263	0.44064738298399
C	7.20412479496219	4.79766983251698	1.65140562082604
H	8.12686346563270	5.36478083594046	1.50513882170060
C	6.42220390267464	5.01373879476883	2.79066784924339
H	6.73401531029098	5.75081170437936	3.53507742249994
C	-1.72586731257656	-0.92868672995752	-0.51710659862599
H	-1.41542598635209	-1.33817824567586	0.45448122097249
C	-4.58254503171373	-6.60923247923335	-2.38950238707530
H	-5.10379024149228	-7.45275571743412	-2.85147954558350
C	-3.99361382312306	-6.75123130807794	-1.12970869853771
H	-4.05493505843221	-7.70930301155856	-0.60441558970968
C	-2.62231070783374	0.26483707766178	-0.29921733281525
H	-3.14087318922606	0.60082347230918	-1.20586304265127
C	-2.83298698601359	0.93834941358355	0.84210857392693
C	-3.76576559141775	2.12368978361979	0.87548350157200
H	-3.24099863101051	3.03679498407776	1.21043541703280
H	-4.59553828489154	1.96094194718393	1.58624328085302
H	-4.20006953140117	2.32252404923773	-0.11505186615046
C	-2.18833855516901	0.61086704299356	2.16448767770166
H	-1.57526729843331	-0.29792361690733	2.12065801049232
H	1.30426908088963	1.50300509311532	2.25218215567569

TS 3c’ → 3d’

O	2.63201188241672	1.51656819356772	1.54056208373321
O	-0.38077057157363	-5.92017793713990	-2.42384811528237
H	-0.80653299223737	2.19435739399467	-3.01328557185205
O	0.78612152259963	-3.64426537968965	-1.51171971648477
O	0.32570168474233	2.02024704059723	5.64242393248086
O	3.93157952745517	3.80751294513157	2.05856943377080
O	-3.16670231353813	-1.30291535924138	-2.59988378165795
C	2.31932039786656	2.11883406385338	2.54382312788078
C	-0.37020459209499	-3.56967117353573	-1.94139318830165
C	-0.98741189596189	-4.87478445636617	-2.48683882499172
C	1.28277611924891	1.69591505230836	3.49505815049819
C	-2.35486385130990	-4.81181074167975	-3.09391218108091
C	1.93842830234342	3.62501415293358	5.06661817467013
C	3.12657082063108	3.40323460328334	2.86015502179859
C	0.31137692534272	-0.38005290600502	2.29953066683571
H	0.08727928000381	-1.40097759138912	2.63091692885962
C	2.88275262118144	4.10656435579679	4.15276202534440
C	0.55354762511309	0.52655069929952	3.36822463867997
H	-0.96523984832292	1.29381495030420	-4.52659100327354
C	0.13345393103428	-0.14364887113651	0.95948734390980
C	-3.05803868938954	-3.60225012396304	-3.10813793725889
C	-1.17894518567710	-2.40162909386097	-1.94145842153694
C	-0.15712214961153	-1.33681606395429	0.08720089117133
H	-0.91237332572703	-1.95650357821756	0.59945591799617
H	0.74640775939811	-1.97340997426876	0.06451701489117
C	1.11420301512635	2.39615414986169	4.79308024707105
C	3.62194343096549	5.25707373808237	4.44803479725514
H	4.35100758445144	5.60245921123005	3.71184750161166
C	-2.47269375099630	-2.33009729904859	-2.53028700267972
C	-2.92601200323949	-5.96660752920513	-3.63396665952158
H	-2.34316485581998	-6.89046937720124	-3.59963959419738
C	1.74241386132038	4.30102970445138	6.27460586678096
H	0.99912834090288	3.90191433521374	6.96774052772523
C	0.12600191413341	1.21909496604079	0.36618384391388
H	-0.67242466707357	1.30254215090289	-0.38384906146025
H	1.08167320826012	1.38720246753937	-0.15766036430043
H	0.00985255334221	2.00502747275725	1.12407036298630
C	-4.33622966313883	-3.56326895790774	-3.66774687752782
H	-4.85412496029391	-2.60153127902762	-3.66053635837626
C	3.42214358689305	5.92336995260971	5.65026381359431
H	4.00208217827542	6.82051257238163	5.88145025674444
C	2.48055740771369	5.44230467154416	6.56561095058394
H	2.32534187992876	5.96521675096541	7.51294043311108
C	-0.61596976990709	-1.11655301601182	-1.36268047320030
H	-1.46105314273132	-0.41295778379507	-1.36384800628741
C	-4.19977963399200	-5.92098553063427	-4.19268856322458
H	-4.64630754060482	-6.82446513632100	-4.61791637432422
C	-4.90476262124481	-4.71428319637626	-4.20719487778732
H	-5.90693995632952	-4.67381260630940	-4.64451062831362
C	0.49984110278952	-0.57440605187775	-2.22664921762724
H	1.45912187130345	-1.09325148507454	-2.10581565623693
C	0.41994040782150	0.41167985032879	-3.13151277124749
C	1.63060921454818	0.83262366128893	-3.92530210392614
H	1.84640315373705	1.90616939895405	-3.77989997030821
H	1.46787979363568	0.68703696565105	-5.00775970630255
H	2.52515318068356	0.26319512435817	-3.63462091271429
C	-0.84624726588394	1.17355717423971	-3.43654567617700
H	-1.74639548503324	0.66358302189859	-3.06528416181902
H	0.08768464652339	0.23124170786812	4.31784980538192

TS 3d’ → 3e’

O	1.80017864811074	-1.63832719365751	-1.18252428369173
O	-4.45958645122422	1.82819175430836	-0.42353430452892
H	3.85252960144945	2.41746008679779	-1.61855837700965
O	-1.99403010453868	0.83058310442485	-0.81264920350770
O	-0.68568315536769	-4.40065448865492	1.76712696698071
O	2.47406275060702	-3.82888036534938	-2.56166963395860
O	-0.27828232127122	4.46155922581621	1.67268317017134
C	1.38411833196581	-2.69576762190348	-0.76751386474094
C	-2.07364445832127	1.88964450401379	-0.18236718963865
C	-3.51700102930929	2.36939735180748	0.10831618194182
C	0.51671392650000	-2.87043069357436	0.40906853821983
C	-3.72178790534715	3.49004057772582	1.07576404243085
C	0.46679709601723	-5.41168765108079	-0.00929358878442
C	1.77322240205488	-3.95343481573405	-1.58827577099511
C	0.13680851925574	-0.40903061960040	1.08678109930115
H	-0.78133593534484	0.16991849091687	1.21696692852231
C	1.28500458506528	-5.28872834041358	-1.13781687711800
C	-0.07039168597923	-1.82246313567318	1.08994974773018
H	3.14628095613311	3.55435011001806	-2.77570826564417
C	1.30335167116100	0.28963712328109	1.06411971523286
C	-2.61114402390556	4.16555897829597	1.58703123156960
C	-0.99940250193632	2.70400483705040	0.27902631064807
C	1.29055062984597	1.79789282471367	1.06340536141925
H	2.33408206486460	2.14316322728616	0.98173760201324
H	0.92214622100745	2.18160222375764	2.02962661179927
C	0.03646582654766	-4.21959585629144	0.80360760464278
C	1.65681029949441	-6.42600167877496	-1.86328307649044
H	2.29444874051538	-6.29024110893158	-2.73965312006023
C	-1.20143211183212	3.79190491528690	1.18288217490237
C	-5.01431326739252	3.86279969385699	1.45304695049943
H	-5.85371223168976	3.31107964031986	1.02236226215732
C	0.02540489604227	-6.67846431149545	0.38565019761880
H	-0.61294279685632	-6.74296662511732	1.26910792693277
C	2.65634530869095	-0.33740858088366	1.10928898599192
H	3.28717584408130	0.19481419562266	1.83964312712910
H	3.12255937659363	-0.22690805811474	0.11587778833132
H	2.63560158827395	-1.40634117478642	1.35945683787136
C	-2.80566279246341	5.21696234064902	2.48523259230131
H	-1.91464288045757	5.72426561363930	2.86204168366177
C	1.21377993475767	-7.68093089011534	-1.46562678712224
H	1.50241307105229	-8.56892071585316	-2.03397707138264
C	0.39584001779133	-7.80584422020916	-0.33779825118281
H	0.04543903123718	-8.79287174779026	-0.02449058600093
C	0.46386970015704	2.47454707885518	-0.07216867618141
H	0.88539049857538	3.49204060322744	-0.10417069898669
C	-5.20267765773295	4.90819840924247	2.35199324865612
H	-6.21299837906012	5.19986050623949	2.65310805702652
C	-4.09305262058751	5.58538131021715	2.86704089448844
H	-4.23746854526079	6.40917037589372	3.57271745620410
C	0.73243883349107	1.78852229924239	-1.39142323045376
H	-0.00349504245006	1.03494525904552	-1.68339951612006
C	1.80947925250530	1.96518644650333	-2.17076057906831
C	2.00661313365644	1.12400434345196	-3.40583654559246
H	2.91317358475628	0.50044558475046	-3.31782664527296
H	2.12976626252301	1.75043942870473	-4.30756966764494
H	1.15770527460470	0.44494307524511	-3.56330395709574
C	2.91885512212520	2.94545919995002	-1.88349407535791
H	2.67489911686166	3.63159893762760	-1.06071742288507
H	-0.90563422004372	-2.15367378378043	1.72074997012058

TS 3e’ → 4’

O	0.08650476113846	-0.41735013460922	2.63156348143627
O	1.05526846916469	-0.38886859012678	-2.88710925478508
H	-0.59591545733451	5.40447018357971	0.28803600238159
O	0.72403647143313	0.22600598729965	-0.28270587058861
O	1.24823912963775	-4.44056457042048	0.50333852383698
O	2.65061348268845	0.38438757416136	2.43987353524133
O	-3.69620787536634	1.54421724699886	-1.25860816657962
C	0.88762942414722	-1.17293869522254	2.07178966183246
C	-0.22554950982483	0.44355887384802	-1.03897457621318
C	-0.02298539271154	0.00731693029228	-2.50767674091454
C	0.52358883852360	-2.39301423471557	1.42490025022874
C	-1.19201212664993	0.06951552102028	-3.44029471605122
C	2.98343083690855	-2.93866470159924	1.05327181810073
C	2.34526726201568	-0.70649888918378	2.02740299936958
C	-1.76521907641614	-1.55828496027876	0.92998254758002
H	-2.34979723488317	-1.40415133967872	0.01808597016745
C	3.36443964787515	-1.67786549148096	1.52744127781476
C	-0.75723487138788	-2.50370647299347	0.84770798027588
H	0.13327010015989	4.37820776519265	-0.96147252279797
C	-1.95622842729432	-0.62688934504676	1.97217750321744
C	-2.40888958830300	0.59360396122706	-2.99275691394631
C	-1.46200059467687	1.06061547078584	-0.69828592547347
C	-2.50317908084577	0.73105904239705	1.64245832132947
H	-2.70388238961011	1.27797785310262	2.57806767130599
H	-3.48031237865735	0.56753162129547	1.15043685214176
C	1.53701021213175	-3.34848478012867	0.96532744418924
C	4.71470654338824	-1.32063068526659	1.55838664398432
H	4.97494517632787	-0.32638679963042	1.92808875718590
C	-2.57790234002396	1.10810361743471	-1.58031706328608
C	-1.06010826540473	-0.38612963139568	-4.75428725129777
H	-0.09107101930535	-0.78332036595791	-5.06651154179494
C	3.96023528888692	-3.83535954954447	0.61569606919051
H	3.62779227619312	-4.80730958492685	0.24507009521496
C	-2.18970384219521	-1.12047169835598	3.36936683839974
H	-3.28023285007908	-1.24907236563766	3.49323740989945
H	-1.83930691828430	-0.39279501574346	4.11316105078263
H	-1.70771187639013	-2.09178942616948	3.53982715164821
C	-3.49247640830121	0.65265174905666	-3.87081220790366
H	-4.42582813755596	1.06822091369358	-3.48413658500675
C	5.68233989762098	-2.21602424522552	1.11691519375347
H	6.73803280808158	-1.93222719450039	1.13361470077759
C	5.30350655365199	-3.47658340443155	0.64682032744854
H	6.06388721161845	-4.18052823818145	0.29779449060693
C	-1.70517989890782	1.65386215872596	0.67600775748163
H	-2.42839164488765	2.46426137796159	0.48952465223551
C	-2.14333166632207	-0.32403646292732	-5.62587363747878
H	-2.04181074816560	-0.68026874247511	-6.65494050467157
C	-3.36158498698039	0.19677233767297	-5.17999124736675
H	-4.21531749530295	0.24731557789482	-5.86257019738839
C	-0.47642623309063	2.26317080450000	1.30707766164497
H	0.05822204111403	1.65849996581462	2.04301656492810
C	0.04269340872396	3.45521916580442	0.98351572135090
C	1.32373941318920	3.92773883951976	1.62073567959211
H	1.18883221785850	4.89225685510727	2.14379551463574
H	2.10217582391794	4.08881383038577	0.85392780323787
H	1.71068339297810	3.18593563994751	2.33349747626980
C	-0.52542635089227	4.36380804436029	-0.07525746975826
H	-1.51986869981067	4.04696273309632	-0.41848602979079
H	-0.82999730351344	-3.26184602632228	0.06012902237517

TS 4’ → 5’

O	0.48516226105395	1.03485766614622	1.62866393239263
O	0.57249922645400	-2.14101439338527	-2.12417198852101
H	-0.58180383029907	5.20996687088434	-3.03041501305797
O	1.07814285499754	0.42552311684252	-1.45873116551595
O	0.65343306490724	-3.70736748942050	1.44731708138104
O	3.05748950578609	1.00090835678002	2.49913922419342
O	-3.47874692068044	1.34582002613019	-1.47127578923725
C	1.12086316200544	-0.15786650721320	1.80500531433807
C	-0.07442307744787	0.06351705097015	-1.44910286990175
C	-0.34865767063043	-1.38925333770874	-1.91851299949575
C	0.48591438010901	-1.34469545774590	1.48119837212531
C	-1.76568117540324	-1.77822953039782	-2.17083754761341
C	2.62092670947281	-2.59028166346456	2.12645500722491
C	2.49190242964066	-0.05549153881994	2.24644299847189
C	-1.48698193117818	-0.11170445690213	0.83092773921466
H	-2.58202892994454	-0.12614506400635	0.78683964137996
C	3.23182642036969	-1.36116927669897	2.40322470780523
C	-0.84122568918697	-1.35291909419615	1.00193915517404
H	-0.12399324233436	3.73375133896908	-3.89336490674619
C	-0.93762321237894	1.04462992212887	1.66467827669368
C	-2.79268502363263	-0.83707680211479	-2.03046829346465
C	-1.22364970425572	0.84226743961539	-0.95286818018249
C	-1.36294919961799	2.34896340377056	1.01376078850832
H	-0.86973657218162	3.20184642993075	1.50577092314648
H	-2.45049229389596	2.45822566720761	1.15331455768553
C	1.19462319621391	-2.64137400954207	1.66333562646792
C	4.55515791959043	-1.33435076668939	2.84218382675024
H	4.99955743038825	-0.35731459951011	3.04531171875972
C	-2.53916037309636	0.55431254825378	-1.51705220305905
C	-2.05644189280670	-3.08055228127709	-2.59234291713422
H	-1.22645906660155	-3.78384468322814	-2.69129894931168
C	3.33346765796164	-3.77850797208745	2.28805512891713
H	2.82335417868210	-4.71717877466141	2.05981681883192
C	-1.41170061925791	0.92211115858135	3.11378080407756
H	-2.51031196784387	0.94938084813639	3.17302807247332
H	-0.99873378323112	1.75116655054508	3.70802883777480
H	-1.06238205848252	-0.02820189149920	3.54366827666511
C	-4.10988990648219	-1.21198408348066	-2.31843049261658
H	-4.88713529060622	-0.45474663022477	-2.19372908039125
C	5.26531314994218	-2.52184339413016	3.00325379973554
H	6.30419396349795	-2.49408118359404	3.34449247060853
C	4.65536329406722	-3.74692359362705	2.72575955961006
H	5.21375828020557	-4.67901578038066	2.84869341841157
C	-1.04472026761005	2.29431903443793	-0.49582637088221
H	-1.84174767863791	2.85344212141707	-1.00307832498758
C	-3.36728644052538	-3.44687136918225	-2.86663749170762
H	-3.59644935237585	-4.46744885952667	-3.18509346031809
C	-4.39611674972421	-2.50696972736225	-2.73149623293132
H	-5.42917200126839	-2.79454489487049	-2.94694650459307
C	0.27700604254492	2.94810072868899	-0.81322291050866
H	1.09008590018495	2.72353000424646	-0.11617810390363
C	0.52163164354341	3.77465051709218	-1.83898449082294
C	1.88838854059693	4.38233545271184	-2.02282901931885
H	1.83791982335328	5.48526901638074	-2.06323777313730
H	2.34195992519726	4.04926975460834	-2.97306534848900
H	2.56757111782570	4.09632532737595	-1.20751560191406
C	-0.47338415718180	4.11667433804884	-2.91840923615709
H	-1.46943044721493	3.69053108122107	-2.73819200757965
H	-1.32631155257722	-2.30272666417351	0.77922919468167

8. References

1. Jung, E. J.; Park, B. H.; Lee, Y. R. *Green Chem.* **2010**, *12*, 2003.
2. Shah, Z. A.; Khan, M. R. *Rec. Nat. Prod.* **2015**, *9*, 169.
3. CrysAlisPro 1.171.42.54a (Rigaku Oxford Diffraction, 2022).
4. (a) Sheldrick, G. M. *Acta Cryst.*, **2008**, *A64*, 112. (b) Sheldrick, G. M. *Acta Cryst.*, **2015**, *A71*, 3.
5. Sheldrick, G. M. *Acta. Cryst.*, **2015**, *C71*, 3.
6. Barbour, L. J. *J. Appl. Cryst.*, **2020**, *53*, 1141.
7. Dolomanov, O.V.; Bourhis, L. J.; Gildea, R. J.; Howard J. A. K.; Puschmann, H. *J. Appl. Cryst.*, **2009**, *42*, 339.
8. (a) Neese, F. *WIREs Comput. Mol. Sci.* **2012**, *2*, 73. (b) Neese, F.; Wennmohs, F.; Becker, U.; Riplinger, C. *J. Chem. Phys.* **2020**, *152*, 224108.
9. Lin. Y.-S.; Li, G.-D.; Mao, S.-P.; Chai, J.-D. *J. Chem. Theory Comput.* **2013**, *9*, 263.
10. Weigand, F.; Ahlrichs, R. *Phys. Chem. Chem. Phys.* **2005**, *7*, 3297.
11. Rappaport, D.; Furche, F. *J. Chem. Phys.* **2010**, *133*, 134105.
12. Marenich, A. V.; Cramer, C. J.; Truhlar D. G. *J. Phys. Chem. B* **2009**, *113*, 6378.
13. Grimme, S. *J. Chem. Theory Comput.* **2019**, *15*, 2847.
14. Bannwarth, C.; Ehlert, S.; Grimme S. *J. Chem. Theory Comput.* **2019**, *15*, 1652.
15. Bannwarth, C.; Caldewyher E.; Ehlert, S.; Hansen, A.; Pracht, P.; Seibert J.; Spicher, S.; Grimmer S. *WIREs Comput. Mol. Sci.* **2020**, *11*, e1493.
16. Pracht, P.; Bohle F.; Grimme S. *Phys. Chem. Chem. Phys.* **2020**, *22*, 7169.
17. Ásgeirsson, V.; Birgisson, B. O.; Bjornsson, R.; Becker, U.; Neese, F.; Riplinger, C.; Jónsson, H. *J. Chem. Theory Comput.* **2021**, *17*, 4929.
